# Effects of education on adult mortality: a global systematic review and meta-analysis

**DOI:** 10.1016/S2468-2667(23)00306-7

**Published:** 2024-01-23

**Authors:** IHME-CHAIN Collaborators, Mirza Balaj, Claire A. Henson, Amanda Aronsson, Aleksandr Aravkin, Kathryn Beck, Claire Degail, Lorena Donadello, Kristoffer Eikemo, Joseph Friedman, Anna Giouleka, Indrit Gradeci, Simon I. Hay, Magnus Rom Jensen, Susan A. Mclaughlin, Erin C. Mullany, Erin M. O'connell, Kam Sripada, Donata Stonkute, Reed J.D. Sorensen, Solvor Solhaug, Hanne Dahl Vonen, Celine Westby, Peng Zheng, Talal Mohammad, Terje Andreas Eikemo, Emmanuela Gakidou

## Abstract

**Background:**

The positive effect of education on reducing all-cause adult mortality is known; however, the relative magnitude of this effect has not been systematically quantified. The aim of our study was to estimate the reduction in all-cause adult mortality associated with each year of schooling at a global level.

**Methods:**

In this systematic review and meta-analysis, we assessed the effect of education on all-cause adult mortality. We searched PubMed, Web of Science, Scopus, Embase, Global Health (CAB), EconLit, and Sociology Source Ultimate databases from Jan 1, 1980, to May 31, 2023. Reviewers (LD, TM, HDV, CW, IG, AG, CD, DS, KB, KE, and AA) assessed each record for individual-level data on educational attainment and mortality. Data were extracted by a single reviewer into a standard template from the Global Burden of Diseases, Injuries, and Risk Factors Study. We excluded studies that relied on case-crossover or ecological study designs to reduce the risk of bias from unlinked data and studies that did not report key measures of interest (all-cause adult mortality). Mixed-effects meta-regression models were implemented to address heterogeneity in referent and exposure measures among studies and to adjust for study-level covariates. This study was registered with PROSPERO (CRD42020183923).

**Findings:**

17 094 unique records were identified, 603 of which were eligible for analysis and included data from 70 locations in 59 countries, producing a final dataset of 10 355 observations. Education showed a dose–response relationship with all-cause adult mortality, with an average reduction in mortality risk of 1·9% (95% uncertainty interval 1·8–2·0) per additional year of education. The effect was greater in younger age groups than in older age groups, with an average reduction in mortality risk of 2·9% (2·8–3·0) associated with each additional year of education for adults aged 18–49 years, compared with a 0·8% (0·6–1·0) reduction for adults older than 70 years. We found no differential effect of education on all-cause mortality by sex or Socio-demographic Index level. We identified publication bias (p<0·0001) and identified and reported estimates of between-study heterogeneity.

**Interpretation:**

To our knowledge, this is the first systematic review and meta-analysis to quantify the importance of years of schooling in reducing adult mortality, the benefits of which extend into older age and are substantial across sexes and economic contexts. This work provides compelling evidence of the importance of education in improving life expectancy and supports calls for increased investment in education as a crucial pathway for reducing global inequities in mortality.

**Funding:**

Research Council of Norway and the Bill & Melinda Gates Foundation.

## Introduction

Worldwide all-cause mortality has declined and this decline is expected to continue.^1^, ^2^ This positive trend is attributable to improvements in a wide range of health determinants, such as health-care access and quality of care, technological advancements, reductions in poverty, access to water and sanitation, labour rights, and, crucially, access to education.^3^, ^4^

Not everyone has benefited equally from these improvements and reducing disparities in mortality rates between socioeconomic groups has become a key objective for many nations and international organisations.^5^, ^6^ An important milestone in these efforts has been the 2008 WHO commission on social determinants of health,^7^ which advocated reducing disparities in mortality by addressing the social factors leading to ill health and mortality.

The positive relationship between increased schooling and better health is well established.^8^, ^9^ The main pathways through which education can improve health are believed to include social and psychosocial, economic, and cognitive benefits.^10^, ^11^, ^12^ As such, education has been recognised as a key determinant to achieve socioeconomic development, gender and social empowerment, and social mobility,^13^ which are all necessary prerequisites to survive and to thrive.^14^ This focus on social determinants of population health is reflected in the UN Sustainable Development Goals (SDGs) in their entirety and, especially, in SDGs 4.1 and 4.3, which aim to ensure that children complete primary and secondary education, and adults have equal access to tertiary education, respectively.^15^

The global distribution of educational attainment has changed dramatically during the past five decades,^16^, ^17^ and these changes have been associated with effects on mortality.^18^, ^19^ In particular, parental education has been highlighted for its effect on child mortality rates, where each additional year of maternal education has been shown to reduce under-5 mortality by 3·0% and each year of paternal education has been shown to reduce this risk by 1·6%.^14^

**Research in context**

**Evidence before this study**

The relationship between increased schooling and better health is known; however, the magnitude of this relationship globally has not been estimated. We searched seven databases (PubMed, Web of Science, Scopus, EMBASE, Global Health [CAB], EconLit, and Sociology Source Ultimate) for publications from Jan 1, 1980, to May 31, 2023, that assessed years of schooling as an independent variable and all-cause mortality as an outcome. We updated this search on June 16, 2023. The most comprehensive existing evidence linking educational attainment and mortality risk consisted of a systematic review of 68 articles, which included a qualitative synthesis and not a meta-analysis of the education and mortality data. Many existing meta-analyses examining the association between education and mortality included data only from high-income countries, were otherwise region-restricted, did not have individual-level data, or included few countries and time periods.

**Added value of this study**

The comprehensive search strategy in our systematic review, in combination with its global scope, exceeds the scale of previous research on educational attainment and mortality. By including only studies with individual-level data, we reduced the potential for bias from unlinked data, ecological study designs, and country-level average estimates. Furthermore, we used a mixed-effects meta-regression tool, which takes into account variation across studies in controlling for age, sex, and marital status; the definition of education; and the reference category. We found that years of schooling had a significant and consistent effect on all-cause mortality risk and the protective effect of education persisted for female and male individuals, across all age groups, and all levels of Socio-demographic Index of the country where the data were collected. These findings are similar to the protective effects of a good diet and physical activity and the harms of risk factors such as smoking and alcohol. Our findings advance the understanding of education as a social determinant of health, quantifying the relationship between increased years of schooling and reductions in all-cause mortality on a global scale. The results highlight the need for increased research on the association between education and mortality in low-income and middle-income countries and reveal areas for future study.

**Implications of all the available evidence**

The Sustainable Development Goals (SDGs), adopted in 2015 by UN member states as part of the 2030 Agenda for Sustainable Development, acknowledge the importance of education in reducing social and health inequalities globally, and include goals that target inclusive and equitable quality education (SDG 4), gender equality (SDG 5), and reduction of inequalities within and among countries (SGD 10). Our findings support the universal role of education for improving health outcomes and that investments to reduce disparities in education can serve as an important driver to reduce disparities in health.

Despite decades of research and increased awareness of the strong and consistent relationship between education and population health, no previous study has attempted to systematically identify the magnitude of the effect that years of schooling have on adult mortality risk. In this study, we aimed to quantify the reduction in all-cause mortality risk associated with increased educational attainment by age and sex at the global level.

## Methods

### Search strategy and selection criteria

In this systematic review and meta-analysis, we assessed studies that estimated the relationship between adult all-cause mortality and educational attainment. We searched PubMed, Web of Science, Scopus, EMBASE, Global Health (CAB), EconLit, and Sociology Source Ultimate from Jan 1, 1980, to Dec 30, 2019. We updated the search to May 31, 2023, on June 16, 2023. We did not include any language restrictions. All languages, except Korean and Persian, were understood by at least two members of our extraction team. For Korean and Persian languages, we had a translator sit with two members of our team. Our search string combined a comprehensive list of socioeconomic-related terms and mortality-related terms (see appendix 1 [p 4] for additional information on quality testing). SS and MRJ hand searched reviews, systematic reviews, and meta-analyses captured by the database search to identify additional relevant studies. Our methods were described in the protocol established before the review and registered with PROSPERO (CRD42020183923).

Screening of titles, abstracts, and full text was done by teams of two reviewers (LD, TM, HDV, CW, IG, AG, CD, DS, KB, KE, and AA) independently using the Rayyan platform.^20^ Discrepancies during screening were resolved by consensus or referred to a third reviewer. We included studies that assessed individual-level data on years of schooling and adult mortality. We excluded studies that relied on case-crossover or ecological study designs to reduce the risk of bias from unlinked data; studies that did not report key measures of interest, such as relative risk (RR) or hazard ratios; and commentaries, editorials, and letters to the editor publication types. Only studies of adults aged 18 years and older were included; however, exceptions were made for eight studies of cohorts containing small proportions of participants (average 2·9%) below that age threshold. When possible, we ran recalculations of raw data to obtain the appropriate effect measures and to maximise the number of papers included in the systematic review. A complete list of study criteria can be found in appendix 1 (pp 4–5) and a list of the ten extraction languages employed in the full-text reading stage, along with additional details, can be found in appendix 1 (pp 4–6).

### Data analysis

Information about the study, including participants, methodology, effect size, and any study-level controls (including demographic, socioeconomic, behavioural, and health-related factors) were extracted into a standard template from the Global Burden of Diseases, Injuries, and Risk Factors Study (GBD). The extraction template and raw extraction data are included in appendix 2. Each extraction was carried out by a single reviewer. For all reviewers involved in extractions (LD, TM, HDV, CW, IG, AG, CD, DS, KB, KE, and AA), a mid-term quality review was done, selecting a 10% random sample of their extraction to ensure accuracy and provide further guidance for harmonised extraction. After extraction, studies underwent further review after being identified based on a series of quantitative checks of essential study characteristics related to effect measures, education exposures, population characteristics, and other factors such as study year, location, and age. Details of these exclusions and methods for standardising non-standard data can be found in appendix 1 (pp 5–7).

In the case of studies that did not report years of education as a numerical value, we used the UNESCO International Standard Classification of Education (ISCED) mapping to determine numerical equivalents. In the rare case that an equivalent could not be determined using ISCED, we obtained this information through alternative sources (eg, government websites or other studies). For studies that defined groups based on literacy, 1–18 years of education was used as the equivalent numerical value for literacy and no years of education was used for illiteracy. Studies that did not provide sufficient detail on key elements such as sample size, population characteristics, or variables used in adjusted models were excluded. In cases where effect measures from dose–response and discrete risk-curves were available from the same study, only the discrete estimates were used in our analysis. SEs of effect metrics that were calculated from the same population were marginally inflated to ensure that these studies, which were primarily from high-income settings, were not accorded undue weight in modelling (appendix 1 p 6).

A mixed-effects meta-regression was done using the MR-BRT package (0.1.0);^21^ technical details are provided in appendix 1 (p 7). We used a ratio model that was able to parameterise RR point estimates with different exposure and reference levels of education (described in Zheng and colleagues^21^). This process entails using exposure ranges as an independent variable in the regression model and can be used with exposure and reference ranges as well as point values. 10% of datapoints were automatically trimmed, consistent with methods employed by other global meta-analyses.^14^, ^22^ Additionally, we deemed fitting for a non-linear dose–response relationship unnecessary after testing (see appendix 1 pp 8, 14–15 for more details).

The meta-regression model incorporated study-level controls as covariates (eg, whether the effect measure reported controlled for age). We selected these study-level controls using relevant theoretical implications, such as including only covariates that did not lie in the causal pathway between education and mortality, as well as availability and distribution of input data by region. See appendix 1 (p 8) for more details. The final selection of covariates included whether the associated effect size controlled for age, sex, and marital status of the individual. We selected marital status as a covariate as numerous studies have identified it as an important determinant of health. Married individuals benefit in terms of emotional and social support, pooled economic resources, engagement in preventive care, and healthier lifestyles.^23^, ^24^ Covariates were included to allow the main effect to vary by the age of the adult. The estimated effect sizes presented here reflect adjustment for these standardised study-level covariates. 95% uncertainty intervals (UIs) are also reported.

To evaluate differences in the effect of education at different ages, we additionally predicted the relationship between education and all-cause mortality risk in distinct age groups. We identified the age associated with each observation from the midpoint of the age range of each study. We constructed the age group categories themselves to reflect commonly accepted stages of adulthood and to achieve an even distribution of datapoints in each category. In each of these predictions, we used sex and marital status as covariates in addition to the varying reference age of interest.

We explored the differential effect of education across Socio-demographic Index levels by fitting separate models on subsets of the data stratified by Socio-demographic Index. The Socio-demographic Index is a composite index that is calculated based on income per capita, average educational attainment, and fertility rates of all geographies and is expressed on a scale of 0–1. We established the Socio-demographic Index level according to the country-year of the observation, using demographic estimates from GBD.^25^ Additionally, we investigated differences between males and females in the reduction of mortality risk by running separate regressions on observations from entirely male or female populations. These sex-stratified models controlled for age only, as there is some evidence to indicate that the effects of marriage differ between sexes.^26^ Finally, we investigated whether any changes in the proportional distribution of the population across given levels of education over time or between cohorts had an undue effect on the aggregate relationship between education and mortality using time-and-cohort disaggregated models.^27^ Sensitivity analyses for the selection and inclusion of individual covariates, predictions associated with these different scenarios, and our approach to standardising non-standard data for each of the models are described in appendix 1 (pp 8–21). Analyses were completed with Python (3.10.9) and R (4.1.2). Statistical code used is publicly available online.

In the meta-analysis framework employed in this Article, we incorporated the uncertainty associated with each individual effect size into the model-fitting process to decrease the risk of bias introduced by differences across study design and quality. To additionally evaluate the presence of publication or reporting bias across studies, we generated funnel plots to visually inspect how individual study effect sizes deviate from the average fit. Each point was plotted with the residual value on the x-axis and reported SEs on the y-axis, with points falling within the funnel consistent with reported uncertainty. Random effect units represent years of education in log space. We statistically tested for publication or reporting bias using Egger's regression test^28^ applied to the residuals of the model. The model also estimates the degree of between-study heterogeneity (γ). Our predictions do not incorporate between-study heterogeneity in calculations of uncertainty but we do provide uncertainty in the estimation of between-study heterogeneity in line with other meta-analyses.^29^ More details on the estimation of γ and other model parameters are presented by Zheng and colleagues.^21^

### Role of the funding source

The funders of this study had no role in the study design, data collection, data analysis, data interpretation, or the writing of the report.

## Results

**Figure 1:**
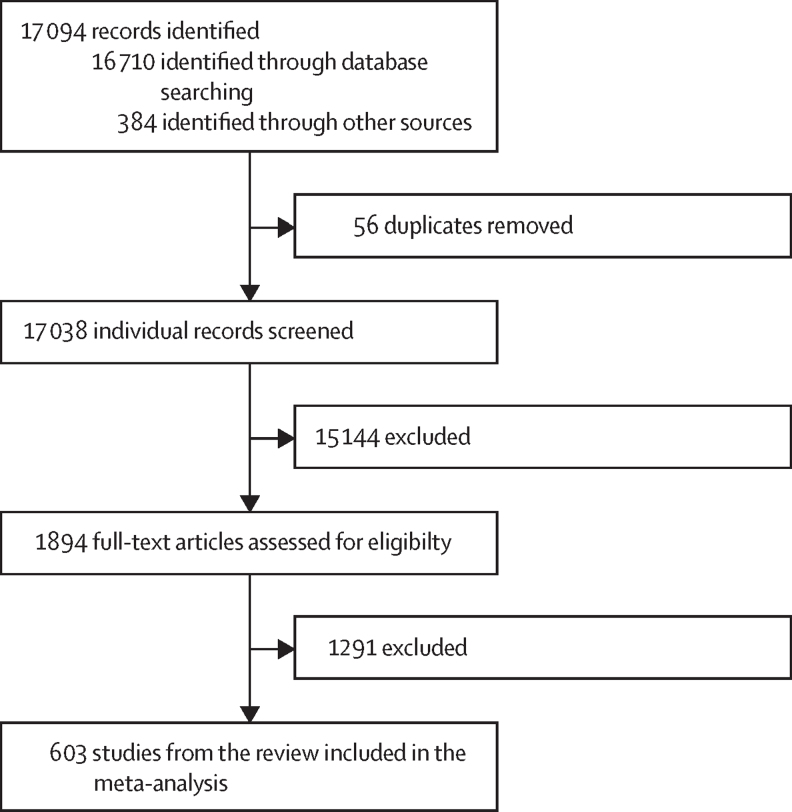
PRISMA diagram

Of the initial 17 094 records found after the systematic review search, 17 038 abstracts were screened, leading to the identification of 1894 publications for full-text screening (appendix 3). The absence of measures of all-cause mortality or any social group analyses, such as measures of socioeconomic axis, including education, but also income or marital status, were the main reasons for excluding studies in the abstract screening phase. The absence of educational attainment metrics was the main reason for exclusion in the full-text screening phase (figure 1).

**Figure 2:**
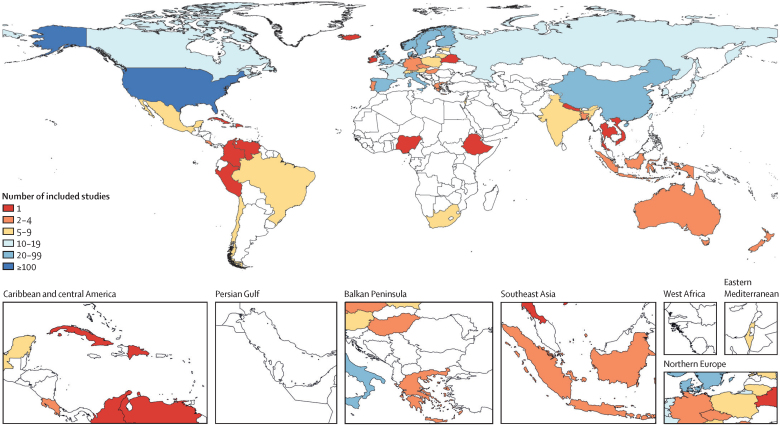
Included studies by location

After screening, 603 studies matched our inclusion criteria and data from the studies were extracted, producing a final dataset of 10 355 observations (table; appendix 3). The studies spanned 59 countries and 70 total unique locations (figure 2). Data were predominantly from studies conducted in the high-income GBD super-region (85·9% of the observations). In contrast, only 0·6% of observations came from sub-Saharan Africa and none came from north Africa and the Middle East. Studies from the high-income super-region were also more likely to report a greater number of effect sizes per study due to differing model specifications than were other regions. Of all observations, 4844 (46·8%) of 10 355 were calculated from population-representative samples, 8548 (82·5%) were collected after 1990, and 8224 (79·4%) were collected in populations with an average age older than 50 years. 7061 (68·2%) of 10 355 observations were from retrospective cohort studies. There were 78 (12·9%) of 603 studies that reported effect measures from dose–response risk curves, 62 (10·2%) of which provided only dose–response effect measures.

**Table:** Summary characteristics of studies included in the systematic review

	**Number of observations, n (%)**
**Age group**	
15–49 years	2131 (20·58%)
50–59 years	2933 (28·32%)
60–69 years	3436 (33·18%)
≥70 years	1855 (17·91%)
**Year interval midpoint**	
1890–99	8 (0·08%)
1960–69	198 (1·91%)
1970–79	215 (2·08%)
1980–89	1386 (13·38%)
1990–99	4698 (45·37%)
2000–09	3258 (31·46%)
2010–22	592 (5·72%)
**Super-region**	
Central Europe, eastern Europe, and central Asia	463 (4·47%)
High income	8893 (85·88%)
Latin America and Caribbean	126 (1·22%)
South Asia	98 (0·95%)
Southeast Asia, east Asia, and Oceania	713 (6·89%)
Sub-Saharan Africa	59 (0·57%)
**Socio-demographic Index level**	
0–0·19	8 (0·08%)
0·20–0·39	82 (0·79%)
0·40–0·59	664 (6·41%)
0·60–0·79	6916 (66·79%)
0·80–1	2685 (25·93%)
**Study population is representative of geography**	
Yes	4844 (46·78%)
No	5511 (53·22%)
**Study design**	
Case-control	20 (0·19%)
Cross-sectional	574 (5·54%)
Prospective cohort	2700 (26·07%)
Retrospective cohort	7061 (68·19%)
**Length of follow-up for retrospective cohort studies (n=6977)**	
0–4 years	1308 (18·75%)
5–9 years	2010 (28·81%)
10–14 years	1401 (20·08%)
15–19 years	653 (9·36%)
20–24 years	561 (8·04%)
≥25 years	1044 (14·96%)
**Study-level controls**	
Age	6985 (67·46%)
Sex	3556 (34·34%)
Race or ethnicity	2993 (28·90%)
Marital status	2417 (23·34%)
Income	1660 (16·03%)
Smoking	1485 (14·34%)
BMI or obesity	1016 (9·81%)
Alcohol use	939 (9·07%)
Employment status	932 (9·00%)
Occupation	790 (7·63%)

The total number of studies was 603 and the total number of effect measures was 10 355.

The most common study-level controls were age, sex, race and ethnicity, and marital status (table), although all controls had variable prevalence across world regions (see appendix 1 p 12). Only 12·1% of effect sizes fully controlled for age, sex, and marital status, and only 30·9% of observations controlled for age and sex. Studies from the southeast Asia, east Asia, and Oceania and high-income super-regions were more likely to include analyses that corrected for race and ethnicity, physical activity, diet, hypertension, diabetes, and religion than were studies from other regions. Additionally, there were marked regional patterns of the socioeconomic status variables included. For example, studies from sub-Saharan Africa and south Asia were more likely to control for wealth or use a wealth index, whereas studies from other regions were more likely to control for income in their analyses.

**Figure 3:**
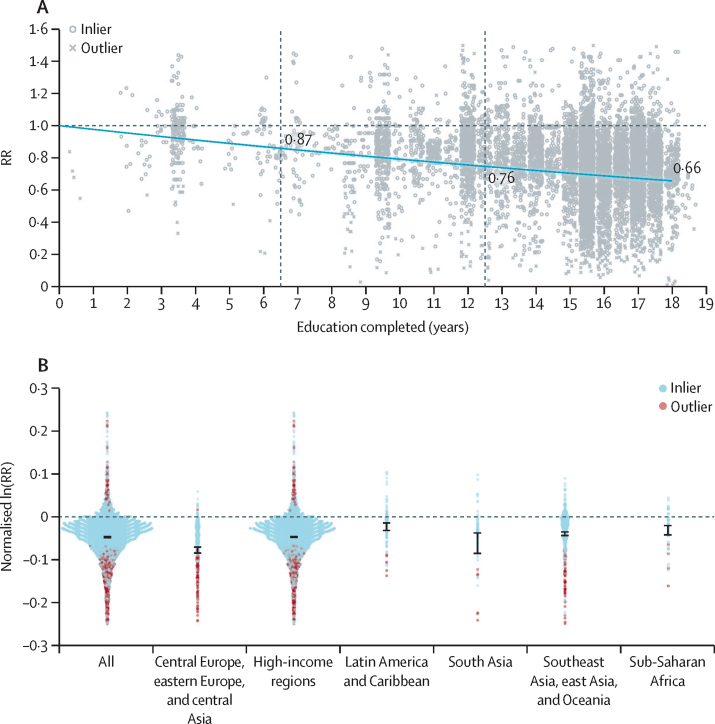
Adult all-cause mortality by education level (A) The relationship between education and all-cause adult mortality risk across the full range of 0–18 years of education, superimposed on the input data of education exposure level and effect size. (B) The distribution of input data effect sizes across super-regions. Normalised ln(RR) can be interpreted as the instantaneous slope of the RR curve implied by each study. Data are superimposed with a synthesised average effect size (shown in black). Ln=natural logarithm. RR=relative risk.

Overall, we find that, compared with no education, completing 6 years of education (roughly a primary school level in most areas of the world) was associated with a 13·1% (95% UI 12·3–14·1) reduction in mortality risk when controlling for age, sex, and marital status. The reduction in mortality risk was 24·5% (23·0–26·1) after 12 years of education (approximate secondary school completion) compared with no education and 34·3% (32·5–36·5) after 18 years of education (approximate completion of tertiary school plus 2 years of post-tertiary schooling). This finding translates to an average reduction in mortality risk of 1·9% (1·8–2·0) per year of education across the 18 years (figure 3).

We did not find evidence of non-linearity or attenuation of the effect of education on mortality risk across the full exposure range (appendix 1 pp 14–15), supporting the finding that there is a reduction in the risk of all-cause mortality for every additional year of education up to 18 years of education.

**Figure 4:**
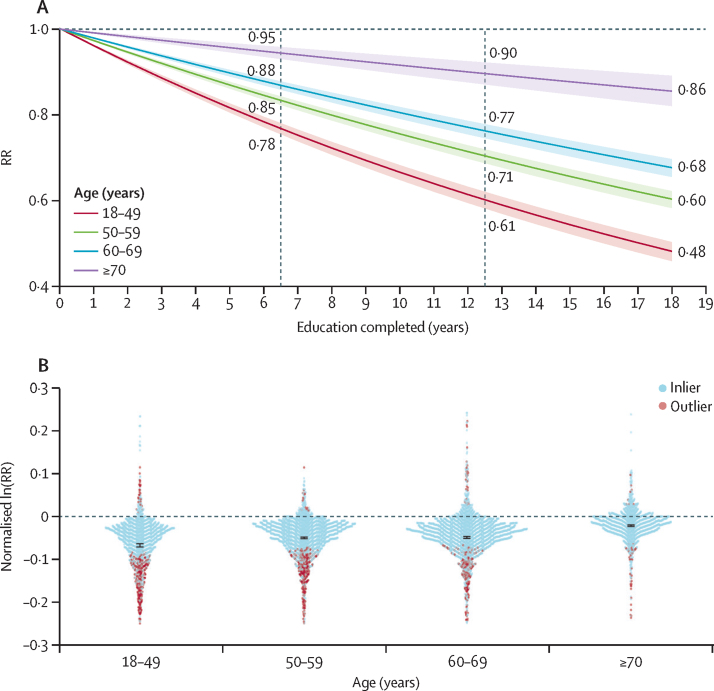
Relationship between education and adult mortality by age group (A) The relationship between education and all-cause adult mortality risk across the full range of 0–18 years of education by age group. (B) The distribution of input data across age groups. Data are superimposed with a synthesised average effect size. Ln=natural logarithm. RR=relative risk.

**Figure 5:**
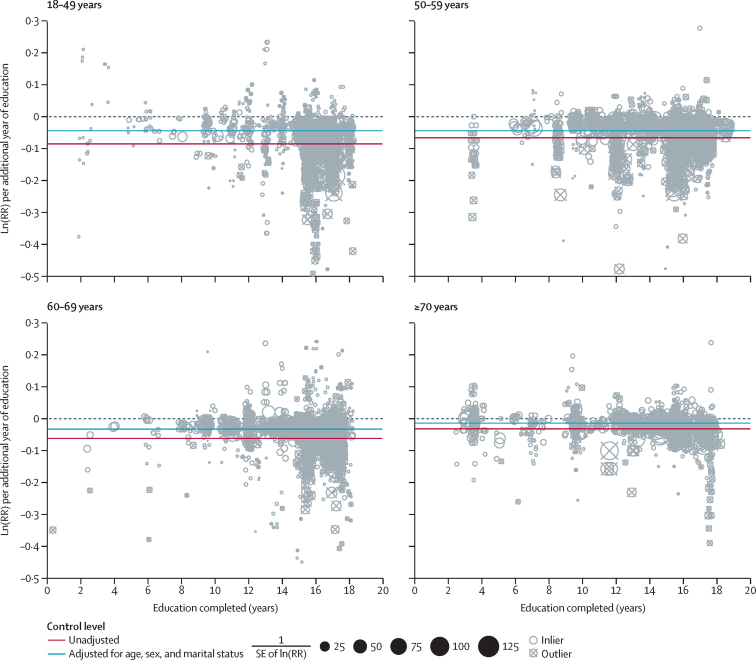
Dose–response relationship The distribution of input data effect sizes across age groups is shown across the exposure range, superimposed by the average ln(RR) of the unadjusted and optimally adjusted observations, by age group. Ln=natural logarithm. RR=relative risk.

We observed distinct effects of education on mortality risk between age groups (ages 18–49 years, 50–59 years, 60–69 years, and 70 years and older). Individuals aged 18–49 years had a 2·9% (95% UI 2·8–3·0) reduction in mortality per year of schooling compared with a 0·8% (0·6–1·0) reduction per year of schooling in adults older than 70 years (figure 4). In figure 4, the slope of the RR curve is negative across all age groups, implying more education is universally protective for mortality. Notably, even after age 70 years, education was significantly protective for mortality across all levels of exposure (figure 5; appendix 1 pp 16).

Separate regressions on observations from entirely male or female populations showed no significant differences in the protective effect of education between sexes, regardless of level of schooling, when using studies from all regions. Among only locations in high-income GBD super-regions, the protective effect of education on male populations was 2·7% (95% UI 2·5–2·8), which was greater than the 2·4% (2·3–2·5) reduction observed in female populations. These differences were not significant in a separate sensitivity analysis of data from all other world regions combined.

We examined the effects of education on mortality risk stratified by country Socio-demographic Index level (shown in appendix 1 p 17). We were unable to determine a clear or consistent difference in effect of education across Socio-demographic Index levels after conducting sensitivity analyses, although there was some evidence of an attenuation of the effect of education at high Socio-demographic Index levels relative to middle Socio-demographic Index levels. Although the Socio-demographic Index-stratified results were sensitive to model specifications, the negative correlation between education and mortality risk remained in all cases.

To investigate variations in the effect of education across our heterogeneous input data, we completed analyses of the differences in the education–mortality relationship across the dimensions of sex, Socio-demographic Index, and World Bank income regions (appendix 1 pp 14–20). We additionally used time-and-cohort disaggregated models to evaluate whether any changes over time in the proportional distribution of the population across given levels of education had an undue effect on the aggregate relationship between education and mortality. We found that variations in the effect of education on mortality across time were not significant, although inequalities in mortality might be greater in more recent cohorts. Further descriptions of the methods and results of these sensitivity analyses are provided in appendix 1 (pp 9, 20).

We identified significant publication bias in our input data (p<0·0001), which occurs when there is a relationship between an estimate's SE and the residual. Nevertheless, many of the datapoints falling outside the funnel of estimated average effect sizes were automatically trimmed in the 10% trimming process used in the meta-regression model (appendix 1 p 15). Of these, 87% were from subgroup analyses. We identified heterogeneity of effect sizes across our large geographically and temporally diverse dataset. The estimate of between-study heterogeneity, or γ, was 0·00025 (95% UI 0·00022–0·00029; appendix 1 pp 8–9).

## Discussion

This systematic review and meta-analysis presents a comprehensive summary of the dose–response relationship between education and adult all-cause mortality. Our findings suggest that a low education is a risk factor for adult mortality, after controlling for age, sex, and marital status. We find that, on average, an adult with 12 years of schooling has a 24·5% (95% UI 23·0–26·1) lower risk of mortality compared with an adult with no schooling, with an average reduction in mortality risk of 1·9% (1·8–2·0) per year of schooling. The protective effect of higher education on mortality was significant across age, sex, period, birth cohort, and Socio-demographic Index level and did not attenuate at higher levels of education.

Through this analysis, we can compare the effects of education on mortality risk with that of other high-impact social determinants of health and behavioural risk factors.^30^ For instance, the 34·3% (95% UI 32·5–36·5) reduction in all-cause mortality risk provided by 18 years of education relative to no education is similar to the reduction in risk of ischaemic heart disease associated with optimal vegetable consumption relative to no vegetable consumption (RR ∼0·77)^31^ and the reduction in all-cause mortality risk for adults meeting physical activity guidelines compared with adults not meeting guidelines for aerobics and strengthening (RR ∼0·60).^32^ The risk of all-cause mortality for an adult with no education compared with 18 years of education is similar to that of lung cancer incidence or mortality for a person who currently smokes (5 pack-years) compared with a person who has never smoked (RR ∼1·52)^33^ and all-cause mortality for a high-volume alcohol drinker compared with an occasional drinker (RR ∼1·41).^34^ These comparisons suggest that the benefits of increased investment in education on future population health are comparable to more commonly discussed public health threats, underscoring the crucial importance of increased and equitable educational attainment as a global health goal.

Notably, the effects of education on mortality risk are known to be mediated by health behaviours. For instance, lower educational attainment is correlated with higher rates of cardiovascular disease and cancer mortality.^35^ Higher education facilitates access to better employment, higher earnings, quality health care, and increased health knowledge.^12^ Moreover, individuals who are more highly educated tend to develop a larger set of social and psychological resources that shape the health and duration of their life.^10^, ^11^ Although some evidence exists that there are diminishing returns of schooling at higher levels, often attributed to the burden of non-communicable disease and some behavioural risk factors in high-income settings,^36^ we did not find evidence of diminishing benefit in these analyses (appendix 1 pp 14–15).

Disaggregation by age group indicates that the protective effect of education is significant across the entire age span, but greatest at younger ages, which is in line with previous studies (figure 4).^35^, ^37^ As an individual reaches older age, genetic disposition, daily habits, diet, or other socioeconomic predictors of mortality appear to have a greater influence on mortality risk than their level of educational attainment.^38^ However, despite these influences, educational inequalities in mortality are persistent across the entire lifespan, and the pattern remains the same across time periods and cohorts. The differences observed across the age groups captured in our dataset contribute to the heterogeneity of all observed effect measures.

Our finding that the protective effect of education did not differ significantly between sexes requires further investigation. Previous studies, particularly from high-income countries, have reported sex differences in the protective effect of education, in which a shift has been observed across time from a stronger protective effect of education among female populations to a more recent marginal advantage for male populations.^37^ This variability between sexes is also seen in lower-income countries.^39^, ^40^, ^41^, ^42^ These region-specific or period-specific trends might be obscured in our global analysis. However, it has been shown that education for female individuals has a stronger intergenerational effect than education in male individuals,^14^ and efforts targeting education in primary and secondary age girls, particularly in the regions of the world where education for girls is still behind education for boys, are still needed to reduce existing inequities in educational attainment and improve future population health.

This meta-analysis is the first to examine the association of Socio-demographic Index levels with educational inequalities in mortality. Studies have shown that increasing Socio-demographic Index levels have gone hand in hand with improvements in health outcomes,^43^ which have accrued largely to countries with a low Socio-demographic Index. We found a similar protective effect of educational attainment at every Socio-demographic Index level present in the available data, emphasising the importance of schooling across all levels of economic development. Several underlying contextual and data-driven reasons might explain this finding: since the Millennium Declaration, variation in Socio-demographic Index level between countries has been decreasing; greater increases in Socio-demographic Index levels in low-income countries than in high-income countries might have been accompanied by increased economic, educational, and gender inequalities; and our analysis is predominantly based on data from high Socio-demographic Index settings given the scarcity of data from lower Socio-demographic Index settings. This relationship should be revisited as the available literature expands and evolves. Alternatively, there might be a universal role of educational attainment in preventing mortality, which is similar between different social contexts.

The findings of this study should be interpreted while taking into account its limitations. First, most studies identified in our review were from high-income settings (figure 2). Given the heterogeneous nature of social inequalities in different income settings and communities,^44^ this limitation highlights the need for additional high-quality research as to the effect of higher education on mortality risk in low-income and middle-income countries. The scarcity of studies from sub-Saharan and north Africa should be addressed in future research and support for this research should be prioritised by relevant funders. Second, we identified variability by region in the quantity and type of study-level controls used across the input studies, which has implications for our analysis and future research (appendix 1 p 12). To minimise any bias resulting from the uneven distribution of controls, we opted to adjust for study-level confounders that we found to have a consistent effect across geographical and economic settings and were present across all regions. This approach is comparable to many other global analyses of the relationship between risk factors and health outcomes. Perhaps most notably, we did not include measures of socioeconomic status, such as wealth or income, in the analysis due to the inconsistency in the variable type and presence across input studies. We cannot control for all sources of confounding in the relationship between education and mortality risk and acknowledge that the effects of education stand behind several essential material, social, and psychosocial resources for health preservation. Thus, controlling for these potential confounders would mean controlling, to some extent, for the effect of education itself. Third, although we limited our review to studies published in 1980 or later, we did not exclude studies that were based on the underlying population years, and, therefore, our data span several decades. This length potentially blurs the influence of medical advances and the associated increasing life expectancy on the effect of educational inequalities on mortality and, although we include sensitivity analyses by time period and cohort, we are unable to make causal claims about the effect of education across the life course. Fourth, although this analysis included studies assessing the effects of education in distinct regions across the world, we are only able to report the average relationship between education and all-cause adult mortality globally and, given the data scarcity, we do not have the power to conduct separate analyses by region. We identified heterogeneity of effect sizes across our large geographically and temporally diverse dataset, which we assume captures factors including between-geography heterogeneity, differences in study population composition, variation in quality and level of adjustment, and other unmeasured differences or non-sampling error across the input studies. A strength of our modelling strategy is that it allows us to estimate a strong signal from these data while incorporating uncertainty from heterogeneity in underlying data. However, studies of more granular effects, such as those specific to region or group,^45^ are needed to increase the efficacy of interventions in education to improve population health.

There is considerable commitment from national and international organisations to reduce premature mortality and increase healthy life expectancy,^15^, ^46^ and, although education is not the only possible strategy to improve health and reduce inequalities, it is one key strategy in concerted efforts to healthier and more equitable societies. However, it is important to note that increasing educational disparities in life expectancy have been observed within high-income settings already experiencing increasing educational attainment.^47^ These disparities are seen in our analyses by birth cohort and in existing studies.^48^ Although there are benefits to increasing educational attainment broadly across populations, it is important to apply the proportionate universalism principle^49^ to investments in education to address existing and increasing health inequalities.

Additionally, the overall decline in mortality rates observed in some high-income countries (before the COVID-19 pandemic^50^) is not sufficient to meet the SDGs.^51^ SDG 4 objectives aim for all children to complete primary and secondary education. Although the world was mostly on track to attain nearly universal primary education before the COVID-19 pandemic,^16^ progress in attainment of higher education is needed to reduce the global health loss attributed to low education.

Previous reports on the magnitude and development of educational inequalities in adult mortality are predominantly available for high-income countries^52^, ^53^ or are heavily focused on infant and child mortality in low-income and middle-income countries.^14^, ^54^ Our research adds to the limited body of research on inequalities in adult all-cause mortality globally and shows that improvements in the social conditions of daily life are necessary for future population health. By increasing years of global schooling, we can help counteract growing disparities in mortality. However, progress will require international commitment and continued investments in educational institutions worldwide. Education cannot remain neglected as a social determinant of health.^30^ Viewing investments in education as investments in health can help address this neglect.

## Data sharing

Data collected during the systematic review, including the full list of articles and raw extracted data is available in appendix 2 and appendix 3 in CSV format. The code used for analysis is available on GitHub at https://github.com/ihmeuw/CHAIN_IHME_adultmortality_edu_sysreview. The data used in these analyses and corresponding results is available at https://ghdx.healthdata.org/record/ihme-data/global-education-adult-mortality.

## Declaration of interests

We declare no competing interests.
