# Supplementary material for: Effects of education on adult mortality: a global systematic review and meta-analysis
Source: Lancet Public Health. 2024 Jan 23;9(3):e155–65. doi: 10.1016/S2468-2667(23)00306-7 (PMC10901745; doi:10.1016/S2468-2667(23)00306-7)
Supplement: Supplementary appendix 1 [file mmc1.pdf]

THE LANCET
Public Health

Supplementary appendix 1

This appendix formed part of the original submission and has been peer reviewed. We post it as supplied by the authors.

Supplement to: IHME-CHAIN Collaborators. Effects of education on adult mortality: a global systematic review and meta-analysis. *Lancet Public Health* 2024; published online Jan 23. https://doi.org/10.1016/S2468-2667(23)00306-7.

Supplementary Appendix 1

Authors and Affiliations 2

Supplementary Methods 4

Search string and screening details 4
Supplementary Table 1. Inclusion and exclusion criteria for systematic review 4
Supplementary Table 2. Studies excluded during screening........ 5
Supplementary Table 3. Studies excluded post-full text screening 5
Analysis methods and displaying of data 6
Supplementary Results 8

Study exposure and reference categories........ 8
Exploration of study-level covariates 8
Impact of confounders 8
Effect monotonicity 8
Supplementary Table 4. Reduction in adult mortality by schooling level........ 8
Supplementary Table 5. Coefficients from meta-analysis........ 8
Sensitivity analyses of impacts of education on adult mortality by age, SDI, sex, time and cohort........ 9
Overview of data availability and effect sizes 10
References 10

Figures 10

Supplementary Figure 1. Distribution of study-level education and reference categories by region 11
Supplementary Figure 2. Distribution of study-level confounders. 12
Supplementary Figure 3. Impact of study-level controls 13
Supplementary Figure 4. Log-space relative risk of adult all-cause mortality per year of education by age and educational group 14
Supplementary Figure 5. Log-space relative risk of adult all-cause mortality per year of education by region and super region........ 15
Supplementary Figure 6. Funnel plots of effect sizes extracted in systematic review 15
Supplementary Figure 7. Age-group sensitivity analysis. 16
Supplementary Figure 8. SDI specification sensitivity analysis. 17
Supplementary Figure 9. Relationship between education and adult mortality by sex 19
Supplementary Figure 10. Cohort and Period sensitivity analysis. 20
Supplementary Figure 11. Normalised relative risks per one year of education, shown for all study data separately by super-region and region. 21
Checklists 22

Table 6. Guidelines on Accurate and Transparent Health Estimate Reporting (GATHER) checklist 22
Table 7. PRISMA-E 2012 Checklist 24
List of articles included in systematic review 26

Authors and Affiliations

First Name(s)	Last Name	Affiliation
Mirza	Balaj	Centre for Global Health Inequalities Research (CHAIN), Department of Sociology and Political Science, Norwegian University of Science and Technology, Trondheim, Norway
Claire A.	Henson	Institute for Health Metrics and Evaluation, University of Washington, Seattle, WA, USA
Amanda	Aronsson	Centre for Global Health Inequalities Research (CHAIN), Department of Sociology and Political Science, Norwegian University of Science and Technology, Trondheim, Norway
Aleksandr	Aravkin	Institute for Health Metrics and Evaluation, University of Washington, Seattle, WA, USA
Kathryn	Beck	Centre for Global Health Inequalities Research (CHAIN), Department of Sociology and Political Science, Norwegian University of Science and Technology, Trondheim, Norway
Claire	Degail	Centre for Global Health Inequalities Research (CHAIN), Department of Sociology and Political Science, Norwegian University of Science and Technology, Trondheim, Norway
Lorena	Donadello	Centre for Global Health Inequalities Research (CHAIN), Department of Sociology and Political Science, Norwegian University of Science and Technology, Trondheim, Norway
Kristoffer	Eikemo	Centre for Global Health Inequalities Research (CHAIN), Department of Sociology and Political Science, Norwegian University of Science and Technology, Trondheim, Norway
Joseph	Friedman	Center for Social Medicine and Humanities, University of California Los Angeles, Los Angeles, CA, USA
Anna	Giouleka	Centre for Global Health Inequalities Research (CHAIN), Department of Sociology and Political Science, Norwegian University of Science and Technology, Trondheim, Norway
Indrit	Gradeci	Centre for Global Health Inequalities Research (CHAIN), Department of Sociology and Political Science, Norwegian University of Science and Technology, Trondheim, Norway
Simon I.	Hay	Institute for Health Metrics and Evaluation, University of Washington, Seattle, WA, USA
Magnus Rom	Jensen	Library Section for Humanities, Education and Social Sciences, University Library, Norwegian University of Science & Technology, Trondheim, Norway
Susan A.	Mclaughlin	Institute for Health Metrics and Evaluation, University of Washington, Seattle, WA, USA
Erin C.	Mullany	Institute for Health Metrics and Evaluation, University of Washington, Seattle, WA, USA
Erin M.	O’connell	Institute for Health Metrics and Evaluation, University of Washington, Seattle, WA, USA
Kam	Sripada	Centre for Global Health Inequalities Research (CHAIN), Department of Sociology and Political Science, Norwegian University of Science and Technology, Trondheim, Norway
Donata	Stonkute	Centre for Global Health Inequalities Research (CHAIN), Department of Sociology and Political Science, Norwegian University of Science and Technology, Trondheim, Norway
Reed J.D.	Sorensen	Institute for Health Metrics and Evaluation, University of Washington, Seattle, WA, USA
Solvor	Solhaug	Library Section for Humanities, Education and Social Sciences, University Library, Norwegian University of Science & Technology, Trondheim, Norway
Hanne Dahl	Vonen	Centre for Global Health Inequalities Research (CHAIN), Department of Sociology and Political Science, Norwegian University of Science and Technology, Trondheim, Norway
Celine	Westby	Centre for Global Health Inequalities Research (CHAIN), Department of Sociology and Political Science, Norwegian University of Science and Technology, Trondheim, Norway
Peng	Zheng	Institute for Health Metrics and Evaluation, University of Washington, Seattle, WA, USA

First Name(s)	Last Name	Affiliation
Talal	Mohammad	Centre for Global Health Inequalities Research (CHAIN), Department of Sociology and Political Science, Norwegian University of Science and Technology, Trondheim, Norway
Terje Andreas	Eikemo	Centre for Global Health Inequalities Research (CHAIN), Department of Sociology and Political Science, Norwegian University of Science and Technology, Trondheim, Norway
Emmanuela	Gakidou	Institute for Health Metrics and Evaluation, University of Washington, Seattle, WA, USA

Supplementary Methods

Search string and screening details

Sample search string

Database: Web of Science

Date of search: initial search 06.12.2019, updated 06.13.2023 for years 2020-2023.

The following keywords were provided to the research librarians by the review coordinator and adapted to each database: Mortality, mortality rate, all-cause mortality, total mortality, death, longevity, survival, life expectancy, Education, educated, educational attainment, education level, socio-economic status, socioeconomic, socioeconomic status, social class, disparities, differences, social inequalities, income, income inequalities, occupation, occupational position, socioeconomic position, occupational inequalities, health inequalities, health equity, schooling, literate, literacy, graduation, years of school, school attendance, diploma, educational status, social status, educational, ethnicity, employment, gender, emigrants, immigrants, poverty, geography, marital status

ALL (education OR educated OR "educational attainment" OR educational OR "educational attainment" OR "education level" OR "socio-economic status" OR socioeconomic OR "socioeconomic status" OR "social class" OR disparities OR differences OR income OR occupation OR "occupational position" OR "occupational inequalities" OR "social inequalities" OR "socioeconomic position" OR "health inequalities" OR "health equity" OR inequalities OR equity OR schooling OR literate OR literacy OR graduation OR "years of school" OR "school attendance" OR diploma OR "educational status" OR "social status" OR ethnicity OR employment OR gender OR emigrant* OR immigrant* OR poverty OR geography OR "marital status") W/10 (mortality OR "mortality rate" OR "all-cause mortality" OR "all-cause mortality" OR "total mortality" OR death OR longevity OR survival OR "life expectancy") AND PUBYEAR > 1979

Search string quality

To test the quality of the search string, titles returned by the initial search were compared to a list of 31 selected articles that complied with our inclusion criteria. Approximately 80% of these test studies were present in the final database selected for systematic review

Supplementary Table 1. Inclusion and exclusion criteria for systematic review

	**Inclusion criteria**	**Exclusion criteria**
**Sample**	No limitations based on the population sample characteristics or size	Studies not providing an accurate sample size for the relevant data
**Phenomenon of interest**	Effect measures of adult mortality (individuals 18 years and older) by education level	
Outcome	All-cause mortality	Cause-specific mortality
Measure of education	Literacy status, years of education, or education level	Unclear definitions of education categories.<br>Different education exposures (eg, general vs vocational) with the same number of years or overlapping years of education
**Design**	Retrospective cohort<br>Prospective cohort<br>Cross-sectional<br>Case-control<br>Randomised controlled trial<br>Non-randomised controlled trial<br>Non-randomised trial	Case-crossover<br>Ecological
**Evaluation**	*Data:* Study utilized individual level data on education and mortality<br>*Measures:*<br>Relative risk<br>Hazard ratio<br>Odds ratio<br>Rate ratio<br>Minimum descriptive data	*Data:* Aggregated-level, country-level, rounded effect sizes or neighbourhood-level only<br>*Measures:*<br>Standardised incidence ratio only<br>Standardised mortality ratio only<br>Time-to-event ratio only<br>Incidence only<br>Risk difference only<br>Relative index of inequality

		Concentration index
**Research type**	Any academic publication (research articles, reviews, etc.) containing quantitative data	Commentaries, editorials, and letters
Criteria are grouped based on the SPIDER model (Sample, Phenomenon of Interest, Design, Evaluation and Research Type		

Languages used in full reading stage

French, Spanish, Norwegian, Swedish, Portuguese, German, English, Romanian, Korean, Japanese

Screening and exclusions

Supplementary Table 2. Studies excluded during screening

Reason for exclusion	Number of studies
**Total excluded studies at full-text screening**	1154
**No mortality**	92
**Cause-specific mortality only**	62
**No individual level**	51
**No education**	652
**Wrong effect measure**	128
**No adults**	47
**Study design**	8
**Publication type**	22
**Definition of education**	8
**Full article not found**	25
**Systematic reviews, meta-analysis, scoping review, narrative review, etc.**	74

Supplementary Table 3. Studies excluded post-full text screening

Study	Reason for exclusion
Kannan VD, Brown TM, Kunitz SJ, Chapman BP. Political parties and mortality: The role of social status and personal responsibility. Soc Sci Med. 2019 Feb; 223:1-7.	Dose–response form effect measure with underlying 12 categories of education shown, upon review were unable to determine lower reference category for standardisation.
Elstad JI, Dahl E, Hofoss D. Skjev inntektsfordeling og geografiske forskjeller i dødelighet [Skewed income distribution and geographical mortality differences]. Tidsskr Nor Laegeforen. 2005 Nov 17;125(22):3082-4. Norwegian.	Dose–response form effect measure with underlying education in five-part scale – from higher university education (code 0) to primary school (code 4), in the model '0 = high, 4 = lowest', upon review unsure about reference category and unable to standardise.
Everett, B. G., Rehkopf, D. H., & Rogers, R. G. (2013). The Nonlinear Relationship between Education and Mortality: An Examination of Cohort, Race/Ethnic, and Gender Differences. Population Research and Policy Review, 32(6)	Dose–response form effect measure with unclear reference group. Additional quality concerns related to education levels and uncertainty measures presented in table 3, unable to standardise.
Sullivan, A. R. (2010). Mortality Differentials and Religion in the U.S.: Religious Affiliation and Attendance. Journal for the Scientific Study of Religion, 49(4), 740–753.	Preprint and/or duplicate of study by same author, published later date, which is included.

Analysis methods and displaying of data

Standardising and displaying non-standard data

Select studies with ages less than 18, but greater than 15, were grouped with 18 years of age to optimise data coverage. List of studies affected:

1. Yang K, Zhang Y, Saito E, Rahman MS, Gupta PC, Sawada N, et al. Association between educational level and total and cause-specific mortality: a pooled analysis of over 694 000 individuals in the Asia Cohort Consortium. BMJ Open. 2019;9(8):e026225.
2. Chaisson RE, Keruly JC, Moore RD. Race, sex, drug use, and progression of human immunodeficiency virus disease. N Engl J Med. 1995;333(12):751-6.
3. Nikoi CA, Odimegwu C. The association between socioeconomic status and adult mortality in rural Kwazulu-Natal, South Africa. Oman Med J. 2013;28(2):102.
4. Razzaque A, Carmichael GA, Streatfield PK. Adult mortality in Matlab, Bangladesh: levels, trends, socio-demographic differentials and causes of death. Asian Population Studies. 2009;5(1):85-100
5. Bopp M, Braun J, Gutzwiller F, Faeh D, Swiss National Cohort Study G. Health risk or resource? Gradual and independent association between self-rated health and mortality persists over 30 years. PLoS One. 2012;7(2):e30795.
6. Weitoft GR, Rosén M. Is perceived nervousness and anxiety a predictor of premature mortality and severe morbidity? A longitudinal follow up of the Swedish survey of living conditions. J Epidemiol Community Health. 2005;59(9):794-8.
7. Batty GD, Shipley MJ, Mortensen LH, Boyle SH, Barefoot J, Gronbaek M, et al. IQ in late adolescence/early adulthood, risk factors in middle age and later all-cause mortality in men: the Vietnam Experience Study. J Epidemiol Community Health. 2008;62(6):522-31.
8. Wilper AP, Woolhandler S, Lasser KE, McCormick D, Bor DH, Himmelstein DU. Health insurance and mortality in US adults. Am J Public Health. 2009;99(12):2289-95.

A total of 18 studies required recalculations of effect measures from raw data. Missing or non-standard format measures of uncertainty including exact and relative p-values, non-95% confidence intervals, and otherers were transformed to standard error and 95% CIs for all effect measures. If no measure of uncertainty was given, standard errors were estimated utilising information on study sample size and other sample-size standard error relationships in the input dataset.

Education exposures or reference values that were given only as relative measures were transformed to closed categories by top-coding to 18 years of education, eg, >6 was changed to 7 to 18. Studies reporting education level as 'illiterate' were assigned 0 years of education, and 'literate' the range of 1 to 18 years of education. Any descriptions utilising standard description of degrees or school levels were transformed to numerical categories using the International Standard Classification of Education (ISCED).

Dose–response estimates (effect per year of education) were reported in 78 studies and were the only measures reported in 62 studies. The underlying exposure range, when known, was used to convert these continuous exposure measures to categorical exposures, and the effect sizes and uncertainty were adjusted accordingly.

Additional manipulations to reported uncertainty were required to ensure that effect measures calculated on the same underlying population were not over-weighted in our model. These studies tended to be from high-income populations, where authors reported effect measures from multiple model versions (for example, with varying adjustment factors). To not overly weight these various models, we calculated SE_{adj}, or the adjusted standard error using the following equation:

$$SE_{adj} = \sqrt{\frac{SE_{orig}}{Rep_n}}$$

Where:

- SE_{orig} is the original standard error of the effect measure
- Rep_n is the effective similarity of the measures, equivalent to the number of times a population was repeated across effect measures within the study

Displaying nonstandard data

For data display purposes, in some of the following figures we represent each effect size as the relative risk or log-space relative risk of adult all-cause mortality per year of education. This is necessary as the included studies had inconsistent referent exposure categories and were thus incomparable when viewed in normal, unadjusted space (i.e., one study may report a relative risk with respect to 0 years of education while another may report a relative risk with respect to completed secondary education). This is consistent with the above treatment of the data by the model. The abbreviated method for normalizing this data for visualization purposes divides the effect size by the distance between the midpoints of the referent and alternate exposure windows as follows:

$$\frac{Log(RR)}{Year\ of\ Schooling} = \frac{Log(RR)}{(ref_{lower} + ref_{upper} + alt_{lower} + alt_{upper})/4}$$

Equations

For the meta-analysis, we included random intercepts for studies so that multiple effect sizes from the same study did not drive results unnecessarily. There was enormous heterogeneity in reported relative risks for mortality across studies and exposure values. Accordingly, and consistent with other uses of this tool 10% of outliers were trimmed. The model took the following form:

$$y = \left(X_{alt} - X_{ref}\right) \times (X_{cov}\beta_{cov} + \beta + \mu)$$

Where:

- y is the log(relative risk)
- X_{alt} and X_{ref} are midpoints for alternative and reference intervals for the exposed and unexposed groups measured in the relative risk effect measure
- X_{cov} is a design matrix containing covariates we control for (age, sex, marital status)
- β_{cov} are covariate multipliers associated with X_{cov} (covariate::education interaction)
- β gives estimates of effect size (effect of unit of education on log relative risk)
- μ is a study specific random effect

Specifically, we used a random intercept in the mixed-effects model to account for the within-study correlation and used an estimated study-specific random slope with respect to the signal to capture between study heterogeneity. We utilised Fisher's information matrix[1] to estimate uncertainty of the heterogeneity, reported in supplementary table 5.

Supplementary Results

Study exposure and reference categories

Education exposure and reference categories were variable across our input data by region, mirroring the underlying educational attainment of the populations. Accordingly, studies from low socio-demographic index areas were more likely to examine the impacts of educational attainment at the primary and secondary school level, whereas high-income areas were more likely to examine the impacts of primary versus secondary or tertiary schooling [Supplementary figure 1]. This compositional bias is significant but was addressed in our modelling framework by allowing the impact of the covariates to vary by education exposure level.

Exploration of study-level covariates

We observed vast differences in the relative prevalence of different study-level controls across regions [Supplementary Figure 2]. These stark gradients reflect both the differences in theoretical mediation pathways for the effect of education on mortality risk across different contexts, and more practically the presence of high-quality linked datasets and other compositional data across regions available to researchers. Patterns of confounder availability and the underlying pathways being studied across the world have implications on our ability to model a global relationship. We opted to utilise only confounders that both have theoretically consistent impacts globally and had sufficient coverage across all regions and Socio-demographic Index (SDI) values present in our input data. Accordingly, the selected confounders included in the model were relatively prevalent across each region and SDI group.

Impact of confounders

The relative change in the predicted relationship between education and adult mortality with and without including individual study-level controls reveals the importance of correcting for age, sex, and marital status. Supplementary figure 3 shows the input data stratified by each of these measures, as well as the average RR change over entire exposure range (1−18 years) for each of these study-level controls, relative to predictions with no study-level controls. Controlling for marital status produces the strongest attenuating effect. We hypothesised a portion of the attenuation in effect of education and mortality when controlling for marital status reflects the impacts of income. Marital status and income (or wealth) have similar and overlapping pathways as mediators[2] since marriage likely increases access to resources and can contribute both a social and financial protective effect.

Effect monotonicity

Supplementary Figure 4 and 5 show the normalised effect sizes extracted from the systematic review and the midpoints of the exposure and referent categories for each extracted effect size as an approximation of the instantaneous slope of the relative risk curve implied by each extracted effect size. This visual provides evidence for the monotonicity of the dose–response relationship between education and adult mortality (ie, the slope of the aggregate relative risk curve is negative across the entire age range, different education exposure ranges, and regions). This approach is complicated by each point having been approximated with a different set of confounders; however, the majority are controlled for at minimum by age or sex.

Supplementary Table 4. Reduction in adult mortality by schooling level

Supplementary Table 4. Percent reduction in mortality across each model and level of schooling.

**Model**	**6 Yrs. Of Schooling**	**12 Yrs. Of Schooling**	**18 Yrs. Of Schooling**
Global – All age, both sexes	13.08% (12.22-13.97)	24.45% (22.93-26)	34.34% (32.34-36.35)
Age 18 to 49	21.77% (20.58-22.92)	38.74% (36.86-40.54)	51.99% (49.77-54.08)
Age 50 to 59	15.55% (14.65-16.44)	28.68% (27.14-30.18)	39.76% (37.8-41.66)
Age 60 to 69	12.24% (11.36-13.14)	22.97% (21.41-24.56)	32.39% (30.32-34.48)
Age 70+	5.19% (3.9-6.47)	10.01% (7.51-12.46)	14.49% (10.87-17.98)

Supplementary Table 5. Coefficients from meta-analysis. Education was modelled as a continuous variable, and all interactive variables are operationalised as such. All variables aside from the main exposure and age are binary variables that interact with continuous education and capture study-level qualities alone.

	Model coefficients

Exposure	
Education, years	–0·071 (–0·075 to –0·067)
Study-level covariates	
Adult sex:education	0·0027 (0·0015 to 0·0038
Adult age:education	–0·0020 (–0·0027 to –0·0013)
Adult marital status:education	0·009 (0·008 to 0·009)
Other covariates	
Study population age:education	0·00064 (0·00057 to –0·00070)
Between-study heterogeneity	
Random slope (γ) estimated from between-study random effects	0·00025 (0·00022 to 0·0003)

Sensitivity analyses of impacts of education on adult mortality by age, SDI, sex, time and cohort

Several analyses were conducted to further clarify the effect of education on mortality risk across dimensions of interest. We would expect to see consistency in the relative relationship between education and mortality risk across dimensions of interest both when fitting the model on a subset of the data by group, and by fitting the model on the entire dataset utilising covariates of interest. While the latter is shown in the main article, we include our results utilising the former here to explain the process of selecting or excluding potential covariates of interest.

Age sensitivity analysis

In contrast to the results in [main paper figure 4], Supplementary figure 7 displays results estimated by splitting the input data into four groups and running models separately for each age group, controlled for by the same standardised covariates (minus age). The results confirm the relative order and magnitude of the effects shown in the main paper indicating that age has a stable modifying effect on the outcome of interest.

Effects of education across SDI groups

We examined whether there was evidence of a differential effect of education on mortality risk across different SDI groups, as determined by the SDI level of the study location in the mean study year [Supplementary figure 8]. We were unable to reproduce consistent findings by SDI group utilising the two methodologies. We observed differential impacts of education by SDI category across both methods of analysis described above when characterising by SDI gradient (quintiles) and while utilising SDI as a binary variable indicating high/low SDI level.

The effect of education in the lower SDI group was sometimes greater and sometimes attenuated relative to high SDI, and the direction of this effect was dependent upon on the cut-off point for high/low SDI (at 0.5, 0.6, 0.66 [global mean for 2022], 0.75 [mean of our data], or 0.8). There may be several reasons driving this pattern, including the sparsity of available data in low SDI countries relative to high SDI locations and the variable education exposures available within each group. Our findings could indicate an exaggerated effect of education at low SDI, but one that attenuates quickly over mid ranges of SDI. Given that the effect of SDI was unstable and sensitive to model specification, we opted to exclude it from our models. However, this lack of clear relationship deserves careful exploration in future analyses.

Effects of education across sexes

Of the 10335 observations included in our final dataset, 29.2% and 30.1% were from entirely female or male population, respectively, when matched on location. The effect of education on each sex was not significantly different when utilising all locations; however, the effect was greater in males when looking in high-income regions only (Supplementary figure 9)

Effects of education across time and cohort

We examined whether there was evidence of a differential effect of education on mortality risk across different time periods [Supplementary figure 10 top], as determined by median year of the study (some of which spanned many years), grouped into 1893-1989,1990 to 1999, and 2000-2022 periods. The distribution of data within these categories is below. This analysis shows that there is a stable relationship between education and mortality across different time periods, as well as stable age patterns in mortality.

Further, we explored the relationship between education and mortality across different birth cohorts [Supplementary figure 10, bottom], as determined by median age group of the study subtracted from the median year of the study, grouped into

1870-1929 cohort, 1930-1949 cohort, and the 1950-1990 cohort. While it is challenging to draw clear conclusions about age-pattern and cohort due to differing distributions of ages across each cohort (no older individuals in recent cohort), the results do demonstrate that there remains stable age-patterns in mortality across all cohorts It appears that inequalities in mortality are greater for more recent cohorts compared to oldest cohorts, and this disparity is clearest in the youngest age-group of our analysis, and less clear in other groups. This increased disparity is likely influenced by changing educational attainment distributions across time, as population-level attainment increases the inequality associated with lesser education increases. However, when considering these cohort-effects alongside the lack of period-effects, and stable age patterns, we conclude that if anything, by not disaggregating or controlling for cohort we may be underestimating the effect of education for more recent cohorts.

Time group	1893-1989 period	1990 to 1999 period	2000-2020 period
Proportion of all data	0. 21	0.47	0.31
Cohort group	1870-1929 cohort	1930-1949 cohort	1950-1990 cohort
Proportion of data	0.32	0.51	0.17

Study representativeness sensitivity analysis

Of the 10 355 observations in our dataset, 46.78% are representative of the geography. Others may be representative of an age, sex, or other sub-group of the geography such as city. To ensure that non-representative observations did not affect the final analysis, we conducted the entire estimation process only with studies that are representative of the geography in which they took place and found that the results did not change significantly. In our main results we have included these studies, but we also find that studies from sub-group analyses make up the majority (87%) of the data points trimmed during the outliering phase of the MR-BRT model. For these two reasons we are confident that any studies from non-representative populations do not significantly influence the effect sizes that we report on in our analyses.

Overview of data availability and effect sizes

Supplementary Figure 11 displays normalised relative risks per one year of education, shown for all study data separately by region. Axis labels are intentionally small, and full study titles corresponding with the y-axis value are provided in supplementary appendix 3 Key. These figures serve two purposes, one of which is to simply provide an overview of data availability and effect sizes. The second is to allow curious readers to cross-reference specific studies to see how their measured effect sizes compare to other studies' effect sizes after standardisation of exposure/reference direction and confidence intervals.

References

1. Biggerstaff BJ, Tweedie RL. Incorporating variability in estimates of heterogeneity in the random effects model in meta-analysis. Stat Med. 1997 Apr 15;16(7):753–68.

2. Robards J, Evandrou M, Falkingham J, Vlachantoni A. Marital status, health and mortality. Maturitas. 2012 Dec;73(4):295–9.

Figures

Supplementary Figure 1. Distribution of study-level education and reference categories by region. Distribution of study-level education reference (purple) and exposure (green) categories, paired for each observation, by super-region. High-income and European/Asian regions typically studied the effect of education on mortality risk at higher exposure levels, eg, secondary compared to tertiary education, while other regions tended to study effect of lower exposure levels, eg, less than primary schooling compared to greater than primary-level attainment.

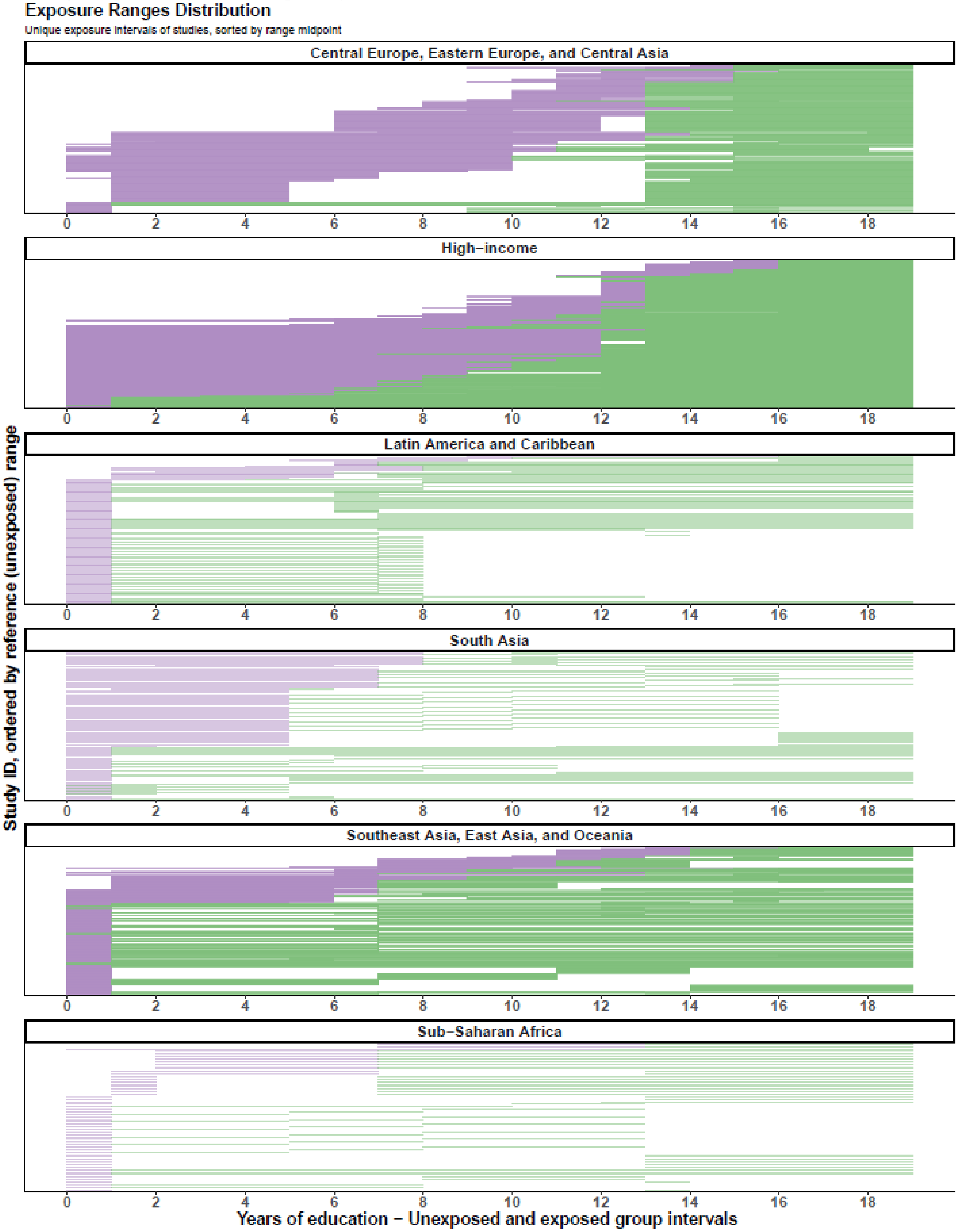

Supplementary Figure 2. Distribution of study-level confounders. Colour indicates the number of studies in region/SDI level that have confounder. Value indicates the percentage of observations that controlled for confounder in region/SDI group.

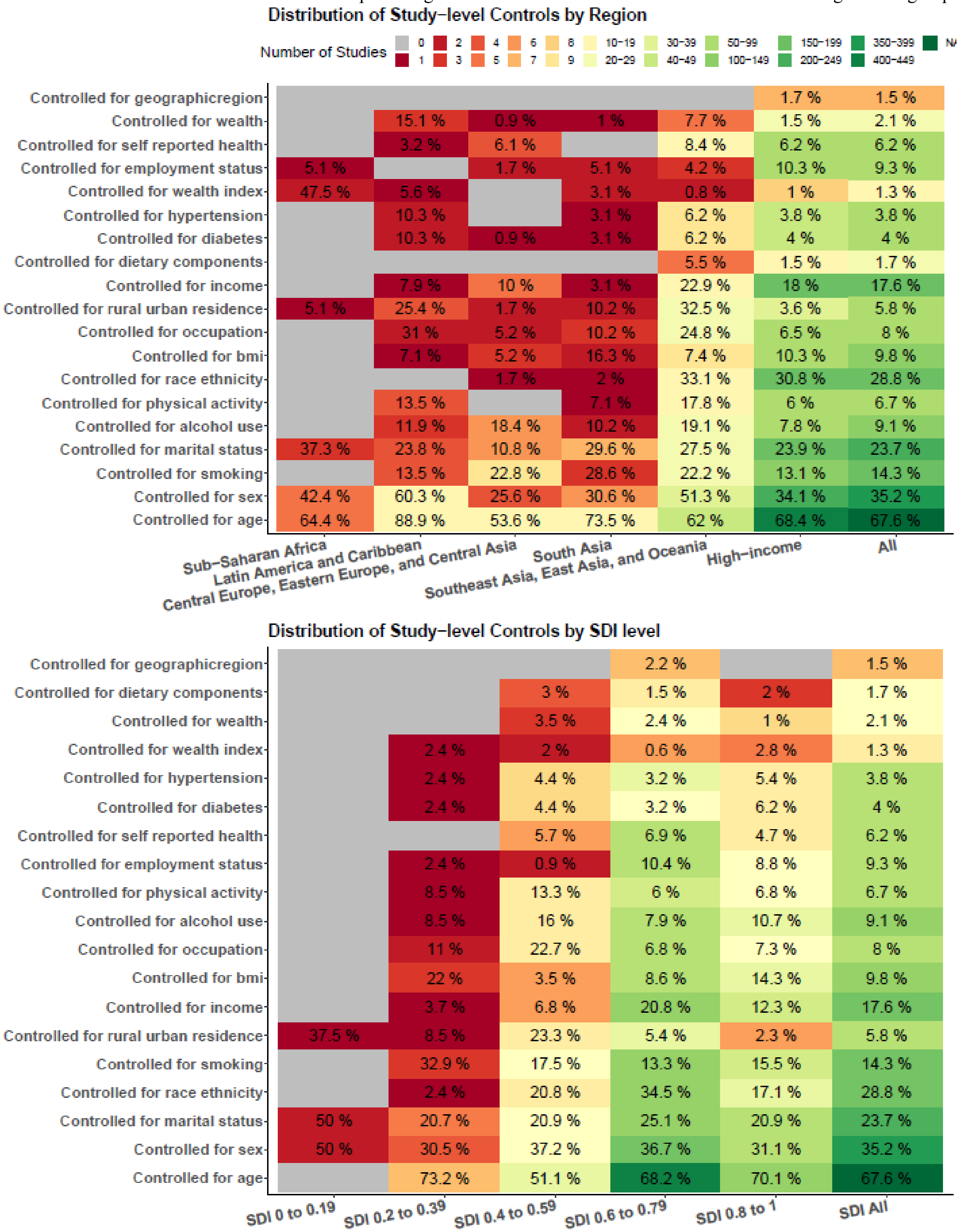

Supplementary Figure 3. Impact of study-level controls. Top) The average RR change over 1−18 years of education when controlling for each of three study-level controls, relative to predictions without adjusting for any study-level controls. Bottom) Scatter of effect measures across exposure range, coloured by study-level control presence with mean effect size.

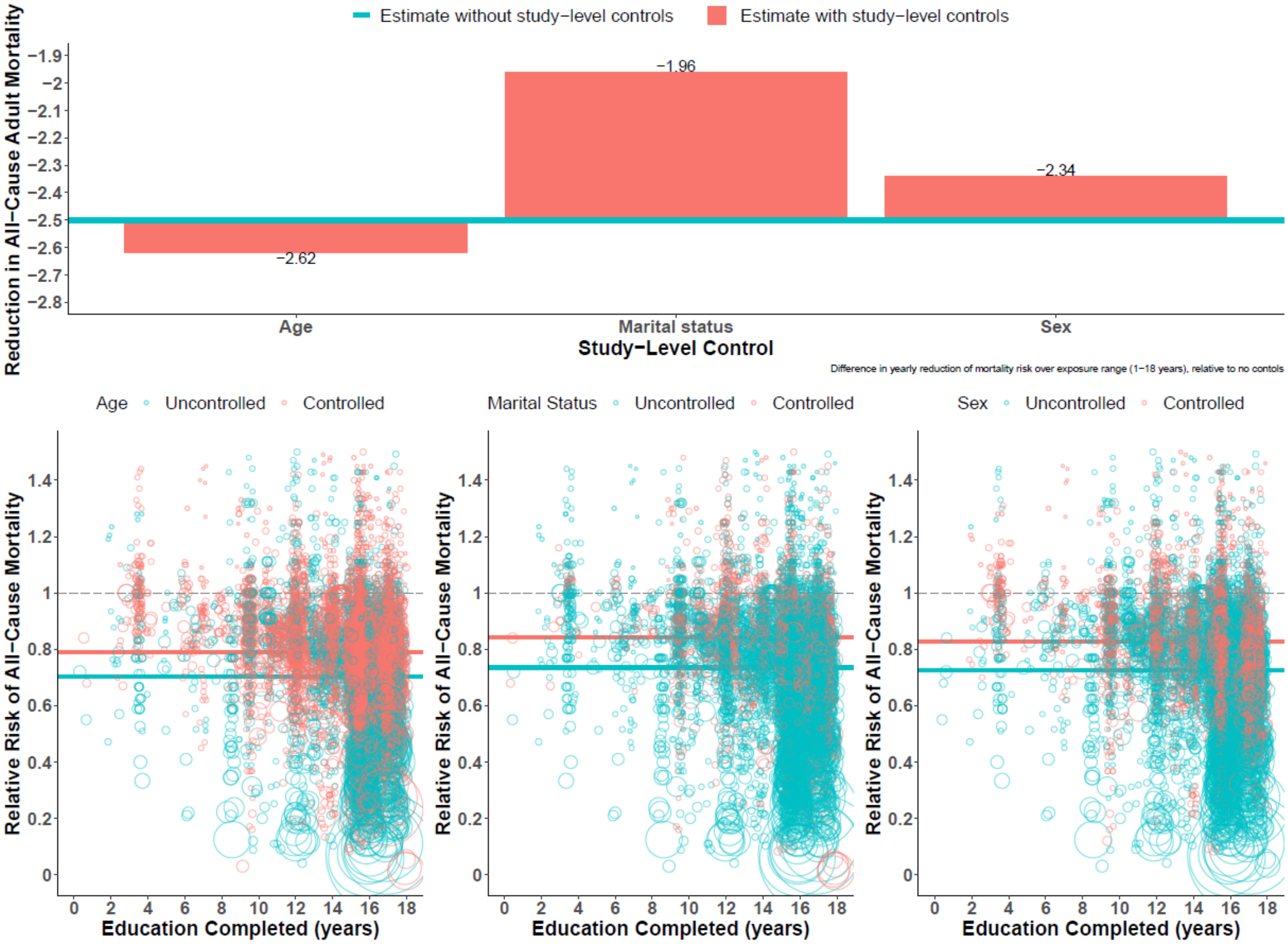

Supplementary Figure 4. Log-space relative risk of adult all-cause mortality per year of education by age and educational group. Effect sizes extracted from the systematic review and the midpoints of the exposure and referent categories for each were used to approximate the instantaneous slope of the relative risk curve implied by each extracted effect size, shown here in log space by top) 10-year age interval, and bottom) approximate level of schooling. Normalised ln(RR) can be interpreted as the instantaneous slope of the RR curve implied by each study; data are superimposed with a synthesised average effect size (shown in black).

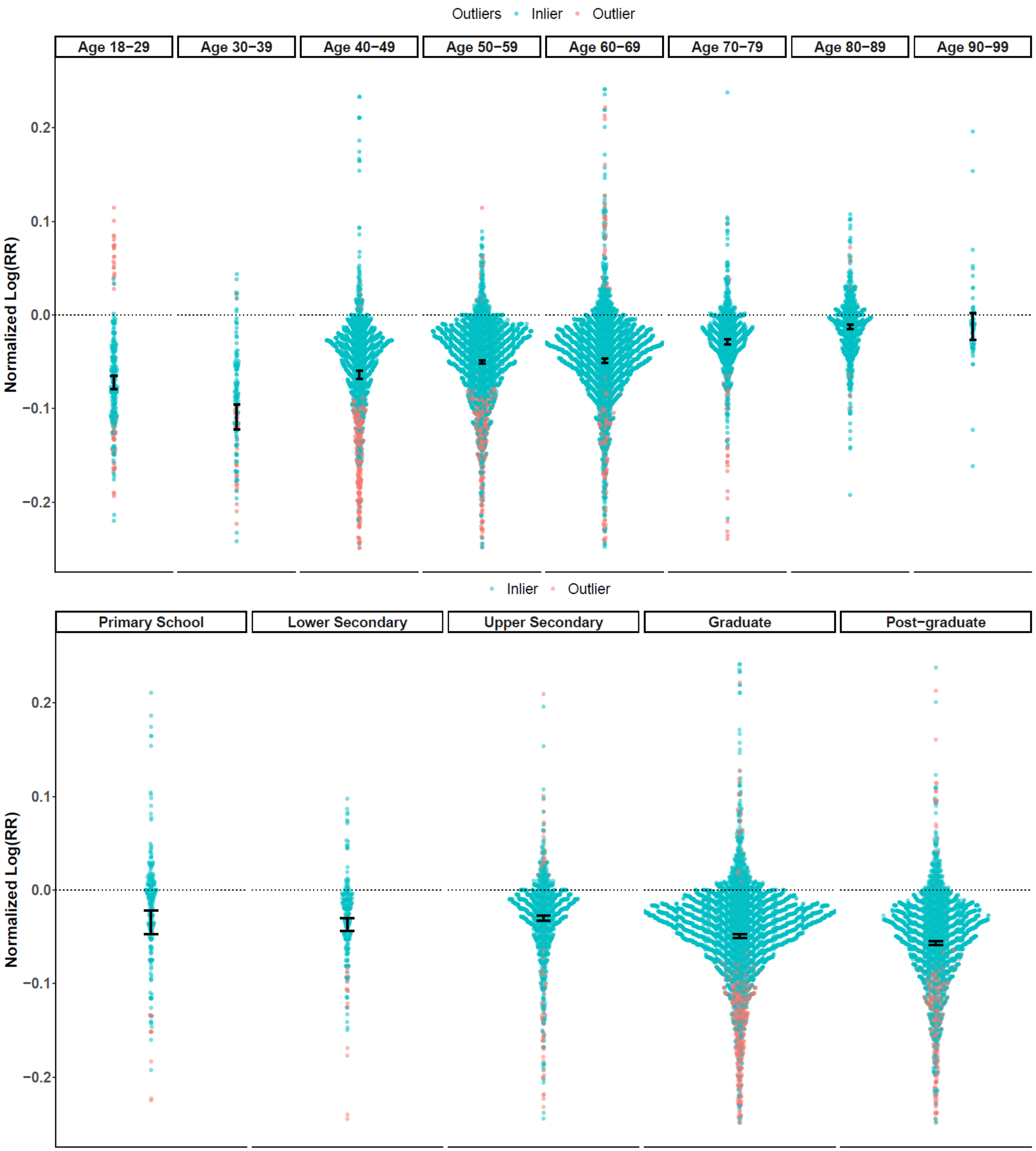

Supplementary Figure 5. Log-space relative risk of adult all-cause mortality per year of education by region and super region. Effect sizes extracted from the systematic review and the midpoints of the exposure and referent categories for each were used to approximate the instantaneous slope of the relative risk curve implied by each extracted effect size, shown here in log space by super-region and region. Normalised ln(RR) can be interpreted as the instantaneous slope of the RR curve implied by each study; data are superimposed with a synthesised average effect size (shown in black).

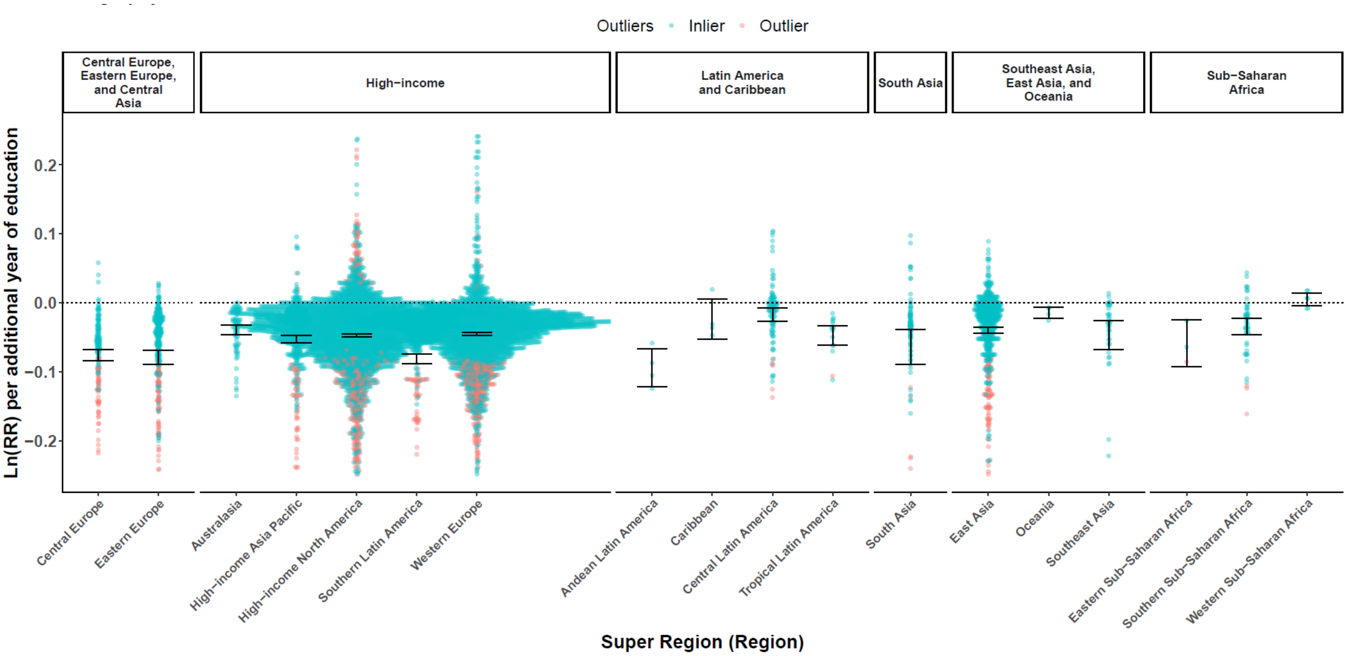

Supplementary Figure 6. Funnel plots of effect sizes extracted in systematic review. Funnel plots show how the effect sizes of RRs from individual studies systematically vary according to the SE of their observations. The residuals are defined as the normalised RR of the study minus the global model fit. Many studies outside of the funnel would indicate study-level heterogeneity and indicate more deviation from the average effect size than would be expected from chance alone. RR=relative risk.

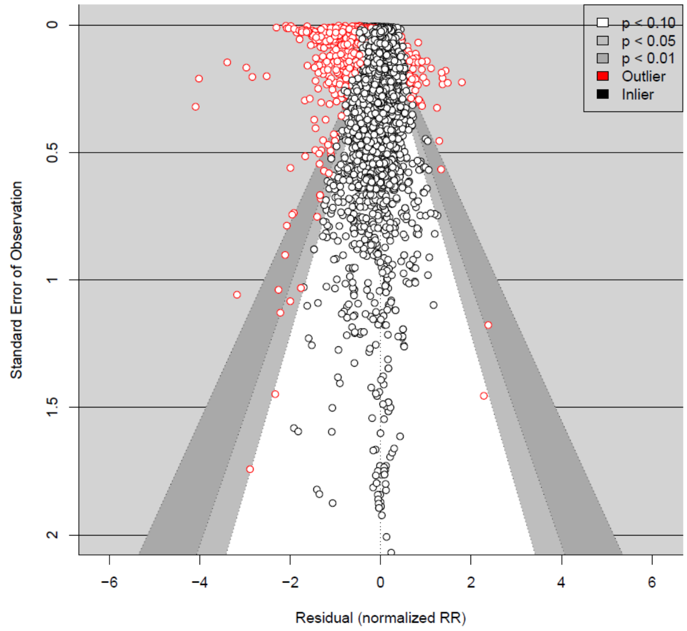

Supplementary Figure 7. Age-group sensitivity analysis. Data were subset by age group and a model was fit utilising the same standard covariates (sex, marital status) without age.

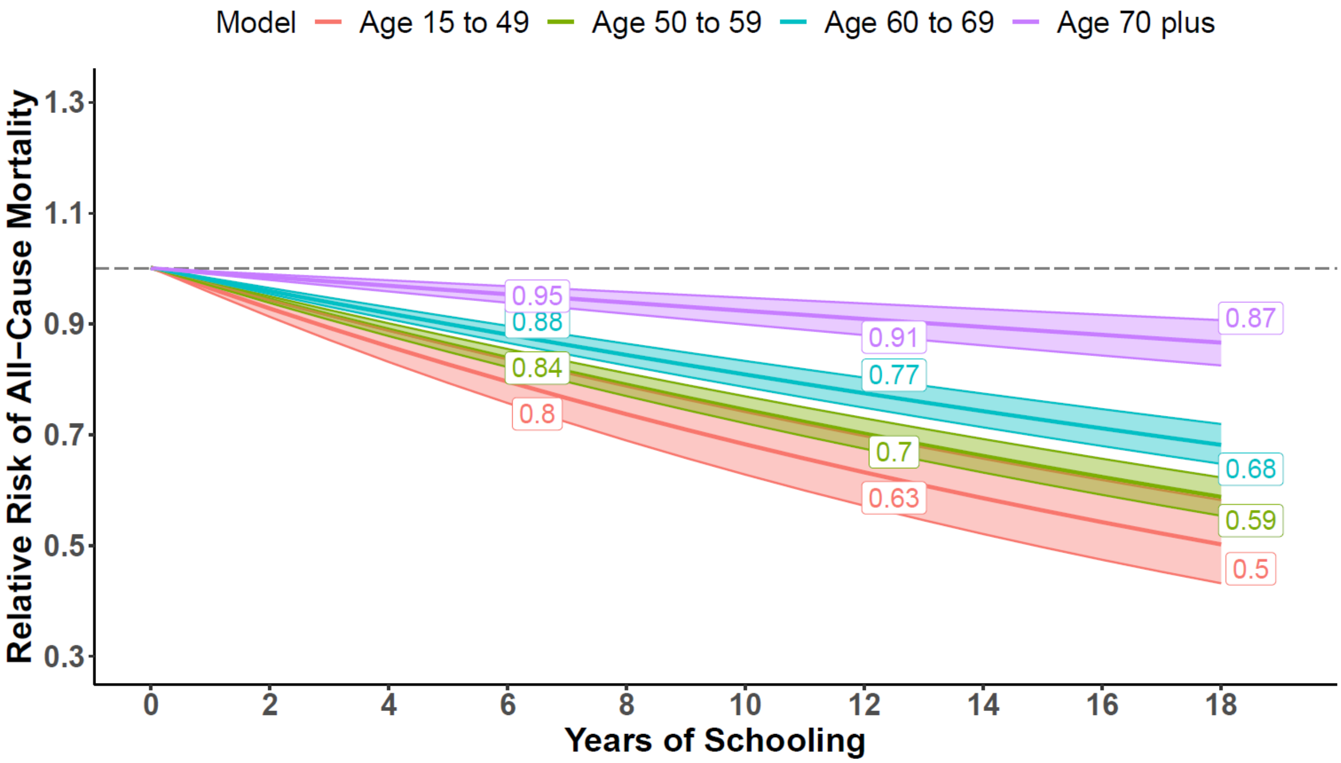

Supplementary Figure 8. SDI specification sensitivity analysis. Data were subset by SDI group and a model was fit utilising the same standard covariates (age, sex, marital status). **Top)** results from subsetting into SDI quintiles (with SDI quintile 1 and 2 combined due to data availability). **Middle)** results from subsetting the data into high/low SDI groups utilising different breakpoints. **Bottom)** results from subsetting the data into groups (1) 0 to <0.7, (2) 0.7 to <0.74, (3) 0.74 to <0.78, (4) 0.78 to <0.82, (5) 0.82 to <0.86, (6) 0.86 to <0.895.

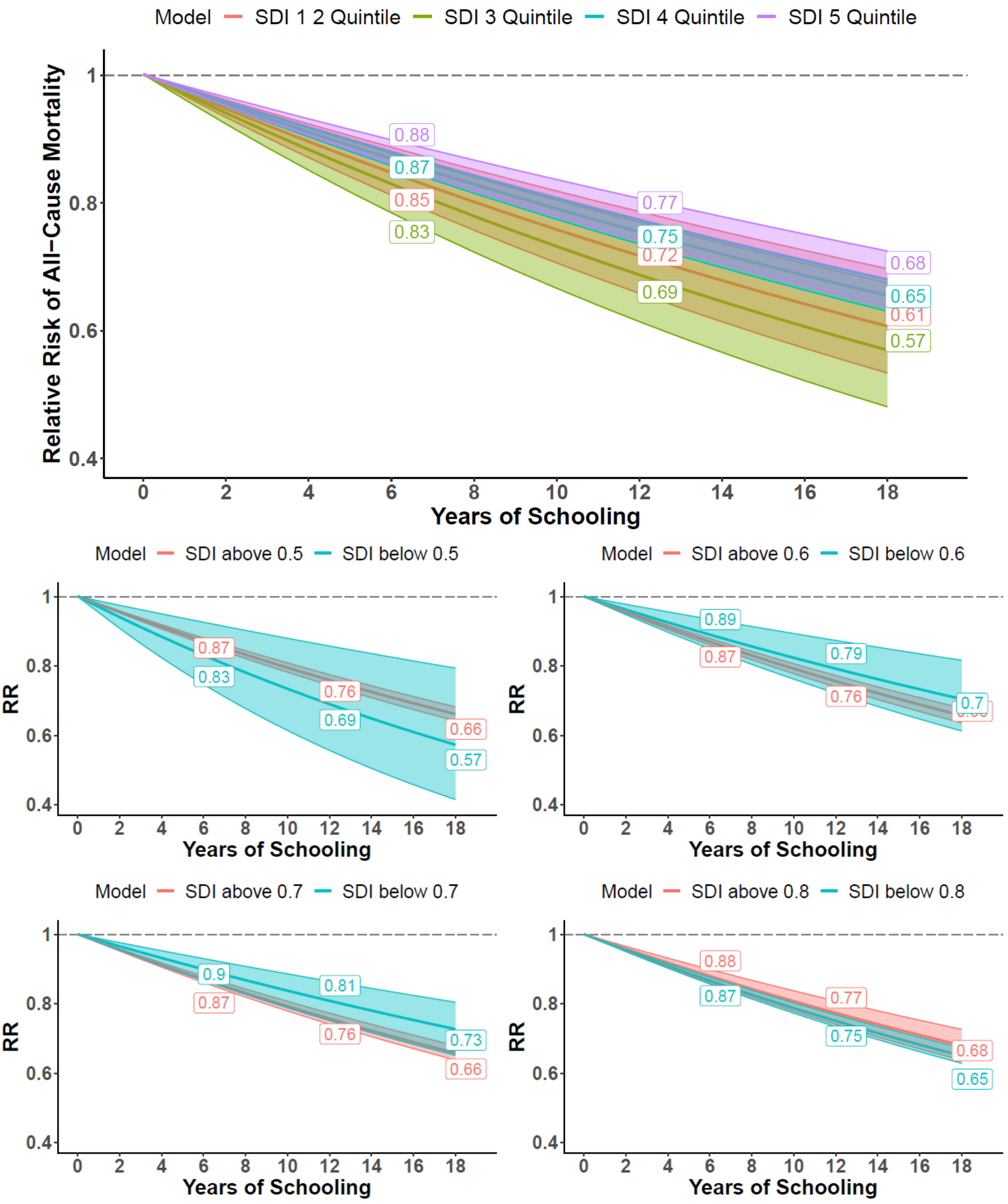

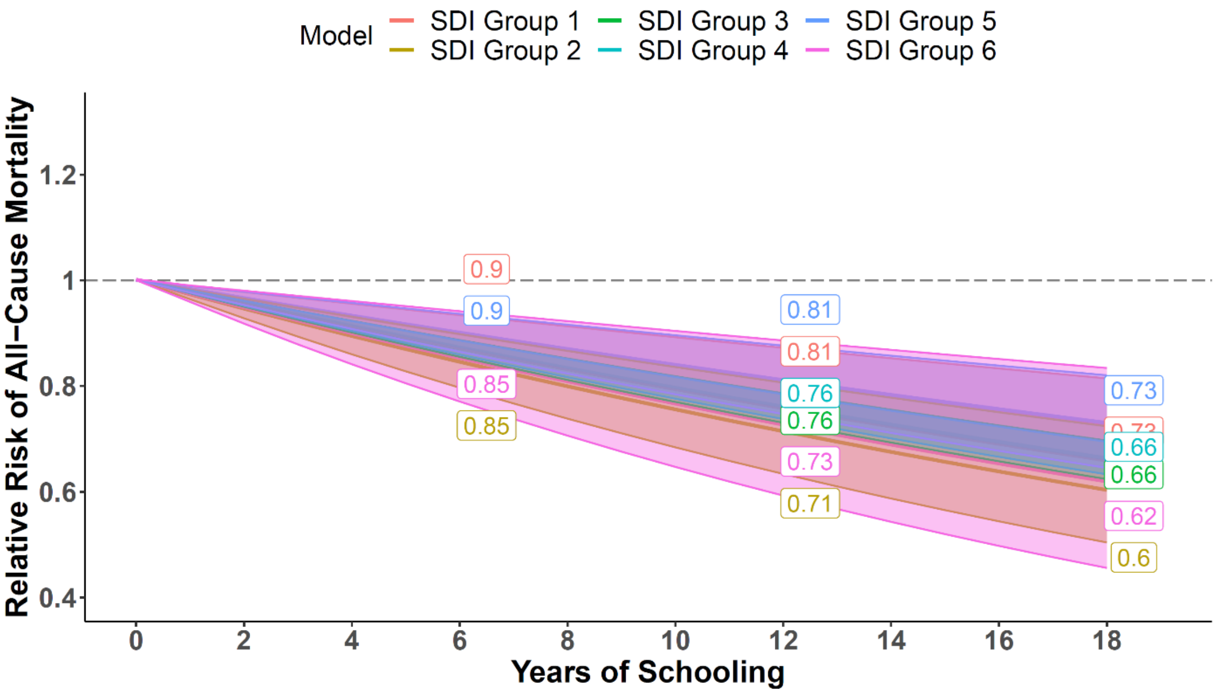
Model
SDI Group 1
SDI Group 2
SDI Group 3
SDI Group 4
SDI Group 5
SDI Group 6
Relative Risk of All−Cause Mortality
1.2
1
0.8
0.6
0.4
0.9
0.9
0.85
0.85
0.81
0.81
0.76
0.76
0.73
0.71
0.73
0.66
0.66
0.62
0.6
0
2
4
6
8
10
12
14
16
18
Years of Schooling

Supplementary Figure 9. Relationship between education and adult mortality by sex. Models were run separately by sex and are controlled for by age only (not marital status or sex). Results are shown from fitting models on entire input dataset (top), high income-region only (left) or all other regions combined (right).

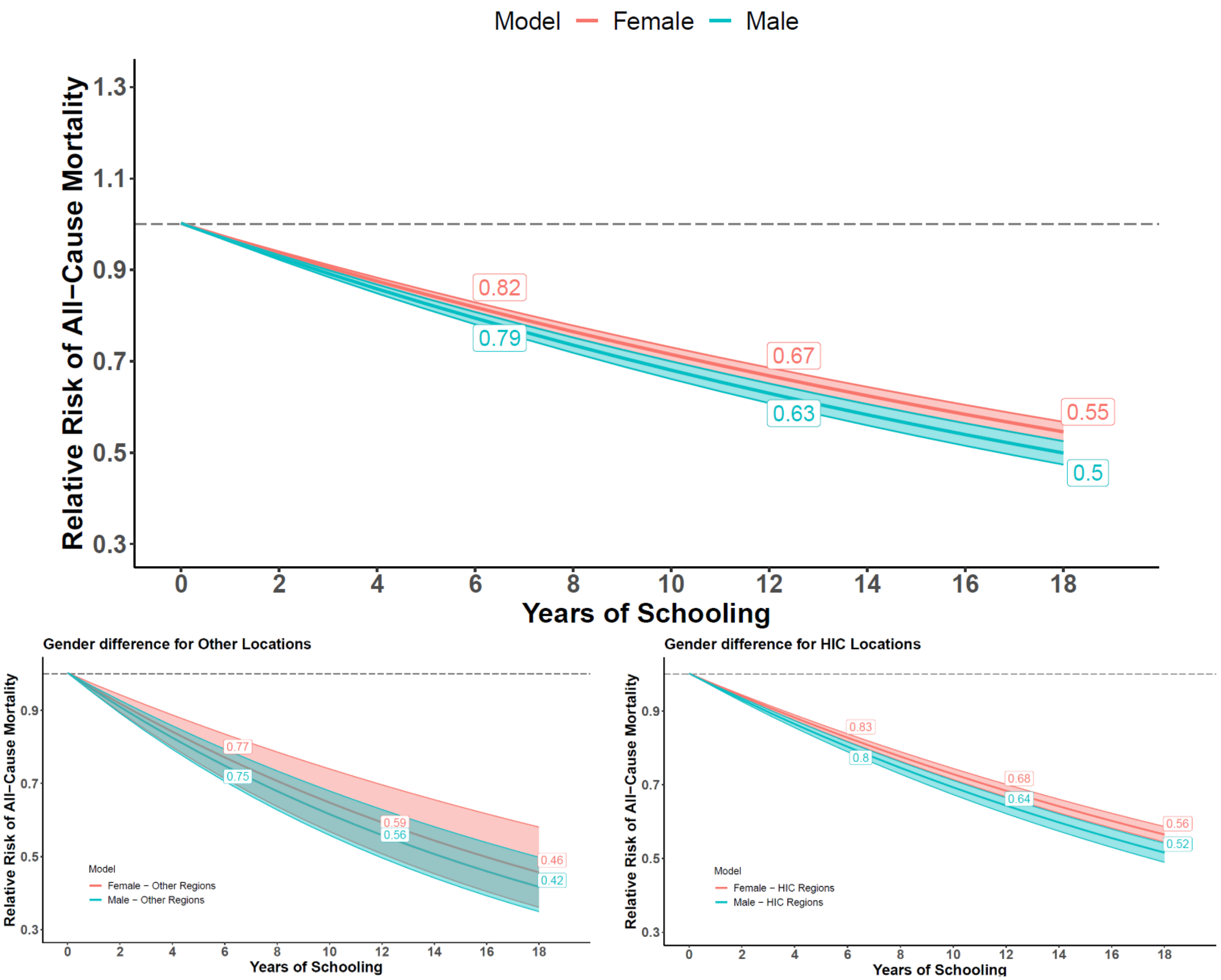

Supplementary Figure 10. Cohort and Period sensitivity analysis. Relationship between education and adult morality by time period (top) and cohort (bottom), additionally stratified by age-group. Models were run separately by time-period or cohort groups calculated from average age and study year, and are controlled for by marital status and sex.

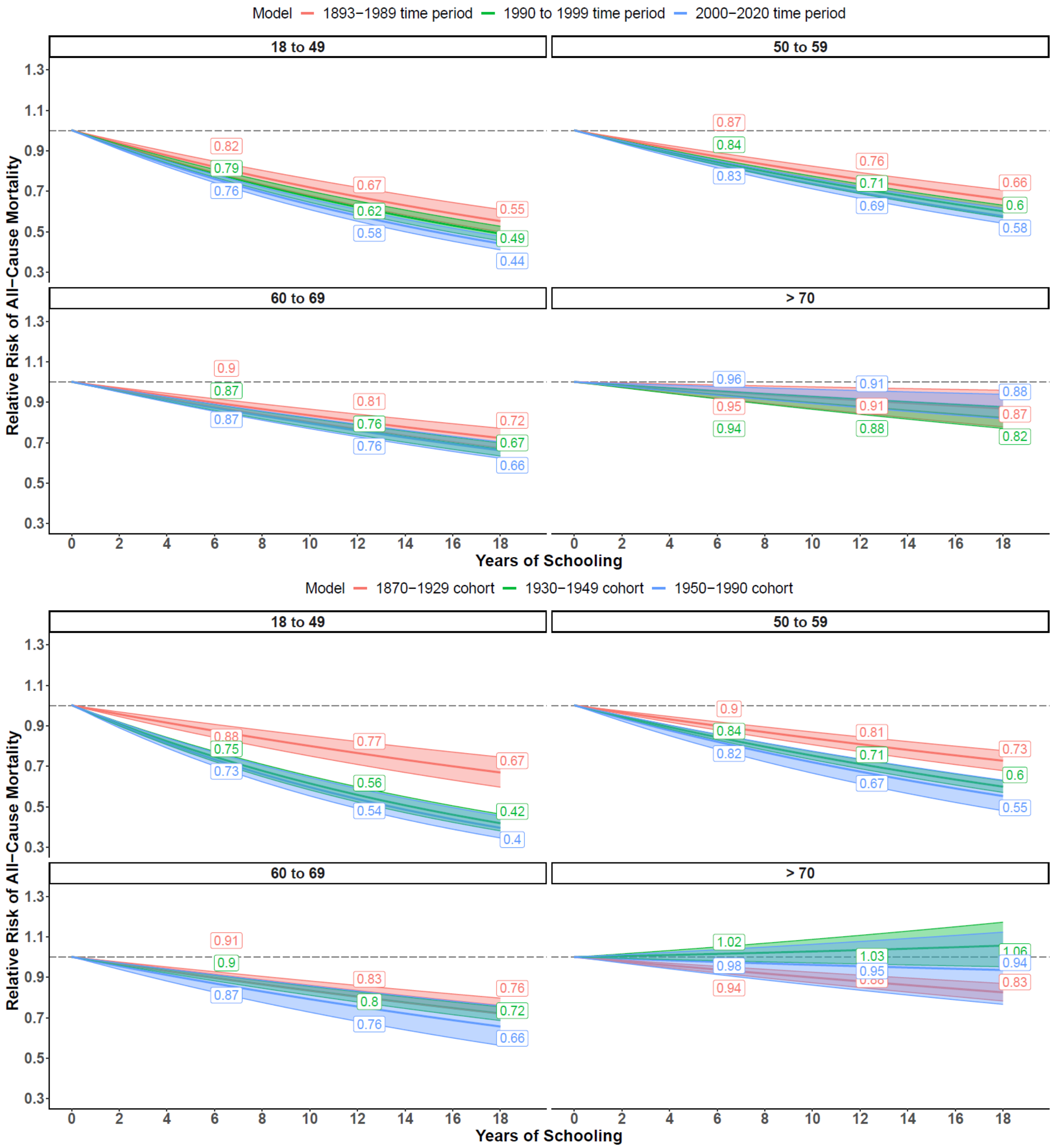

Supplementary Figure 11. Normalised relative risks per one year of education, shown for all study data separately by super-region and region. [End of Document] Normalised relative risks per one year of education, shown for all study data separately by super-region and region. Colour indicates whether the effect measure was automatically trimmed in the global model. Full study titles corresponding with axis label are provided in the supplementary Appendix 3.csv.

Checklists

Table 6. Guidelines on Accurate and Transparent Health Estimate Reporting (GATHER) checklist

Item #	Checklist item	Reporting location
Objectives and funding		
1	Define the indicator(s), populations (including age, sex, and geographic entities), and time period(s) for which estimates were made.	Main text methods, page 2-3 & appendix pp 4
2	List the funding sources for the work.	Abstract and Main text Funding section, page 10
Data Inputs		
For all data inputs from multiple sources that are synthesized as part of the study:		
3	Describe how the data were identified and how the data were accessed.	Main text Methods, pages 2-5
4	Specify the inclusion and exclusion criteria. Identify all ad-hoc exclusions.	Main text Methods includes overview of criteria on appendix page 4. Supplementary Table 2 incudes detailed inclusion and exclusion criteria. Supplementary Appendix 1 table 3 includes details on exclusions from PRISMA diagram and additional details on criteria.
5	Provide information on all included data sources and their main characteristics. For each data source used, report reference information or contact name/institution, population represented, data collection method, year(s) of data collection, sex and age range, diagnostic criteria or measurement method, and sample size, as relevant.	Main text results page 5 and 6, Supplementary table 1 pp4, supplementary appendix 2 (all raw data) and supplementary appendix 1 includes all included study citations.
6	Identify and describe any categories of input data that have potentially important biases (e.g., based on characteristics listed in item 5).	Results section pp 5-7, details in supplementary appendix 1.
For data inputs that contribute to the analysis but were not synthesized as part of the study:		
7	Describe and give sources for any other data inputs.	Sociodemographic estimates from the Institute for Health Metrics and Evaluation were utilized, citation: Wang H, Abbas KM, Abbasifard M, Abbasi-Kangevari M, Abbastabar H, Abd-Allah F, et al. Global age-sex-specific fertility, mortality, healthy life expectancy (HALE), and population estimates in 204 countries and territories, 1950–2019: a comprehensive demographic analysis for the Global Burden of Disease Study 2019. The Lancet. 2020 Oct 17;396(10258):1160–203.
For all data inputs:		
8	Provide all data inputs in a file format from which data can be efficiently extracted (e.g., a spreadsheet rather than a PDF), including all relevant meta-data listed in item 5. For any data inputs that cannot be shared because of ethical or legal reasons, such as third-party ownership, provide a contact name or the name of the institution that retains the right to the data.	Data inputs in excel format available in appendices 2-3.
Data analysis		
9	Provide a conceptual overview of the data analysis method. A diagram may be helpful.	Main text methods overview, PRISMA flow diagram.
10	Provide a detailed description of all steps of the analysis, including mathematical formulae. This description should cover, as relevant, data cleaning, data pre-processing, data adjustments and weighting of data sources, and mathematical or statistical model(s).	Main text methods, supplementary appendix 1

11	Describe how candidate models were evaluated and how the final model(s) were selected.	Main text methods, 'Meta-regression combining data from systematic review' section, appendix 1
12	Provide the results of an evaluation of model performance, if done, as well as the results of any relevant sensitivity analysis.	Main text results section 'disaggregated mortality risk by age group, sex, and Socio-demographic Index', and also appendix 1 supplementary results section.
13	Describe methods for calculating uncertainty of the estimates. State which sources of uncertainty were, and were not, accounted for in the uncertainty analysis.	Main text methods, 'Meta-regression combining data from systematic review' section, appendix 1
14	State how analytic or statistical source code used to generate estimates can be accessed.	GitHub URL provided at resubmission
Results and Discussion		
15	Provide published estimates in a file format from which data can be efficiently extracted.	Estimates are available in the main text
16	Report a quantitative measure of the uncertainty of the estimates (e.g. uncertainty intervals).	UIs given for all findings, including in the text and tables in the main text.
17	Interpret results in light of existing evidence. If updating a previous set of estimates, describe the reasons for changes in estimates.	Main text Discussion
18	Discuss limitations of the estimates. Include a discussion of any modelling assumptions or data limitations that affect interpretation of the estimates.	Main text Discussion

Table 7. PRISMA-E 2012 Checklist

Checklist of Items for Reporting Equity-Focused Systematic Reviews				
**Section**	**Item**	**Standard PRISMA Item**	**Extension for Equity-Focused Reviews**	**Pg #**
**Title**				
**Title**	1	Identify the report as a systematic review, meta-analysis, or both.	Identify equity as a focus of the review, if relevant, using the term equity	1
**Abstract**				
**Structured summary**	2	2. Provide a structured summary including, as applicable: background; objectives; data sources; study eligibility criteria, participants, and interventions; study appraisal and synthesis methods; results; limitations; conclusions and implications of key findings; systematic review registration number.	State research question(s) related to health equity.	1 and appendix table 1 and 2
	2A		Present results of health equity analyses (e.g. subgroup analyses or meta-regression).	1
	2B		Describe extent and limits of applicability to disadvantaged populations of interest.	2 (RIC)
**Introduction**				
**Rationale**	3	Describe the rationale for the review in the context of what is already known.	Describe assumptions about mechanism(s) by which the intervention is assumed to have an impact on health equity.	1-2 (intro), 7-11 (discussion)
	3A		Provide the logic model/analytical framework, if done, to show the pathways through which the intervention is assumed to affect health equity and how it was developed.	
**Objectives**	4	Provide an explicit statement of questions being addressed with reference to participants, interventions, comparisons, outcomes, and study design (PICOS).	Describe how disadvantage was defined if used as criterion in the review (e.g. for selecting studies, conducting analyses or judging applicability).	1 (intro)
	4A		State the research questions being addressed with reference to health equity	1 (intro)
**Methods**				
**Protocol and registration**	5	Indicate if a review protocol exists, if and where it can be accessed (e.g., Web address), and, if available, provide registration information including registration number.		2
**Eligibility criteria**	6	6. Specify study characteristics (e.g., PICOS, length of follow-up) and report characteristics (e.g., years considered, language, publication status) used as criteria for eligibility, giving rationale.	Describe the rationale for including particular study designs related to equity research questions.	2 (Search strategy and selection criteria), table 1, Appendix 1
	6A		Describe the rationale for including the outcomes - e.g. how these are relevant to reducing inequity.	
**Information sources**	7	Describe all information sources (e.g., databases with dates of coverage, contact with study authors to identify additional studies) in the search and date last searched.	Describe information sources (e.g. health, non-health, and grey literature sources) that were searched that are of specific relevance to address the equity questions of the review.	(Search strategy and selection criteria), Appendix 1
**Search**	8	Present full electronic search strategy for at least one database, including any limits used, such that it could be repeated.	Describe the broad search strategy and terms used to address equity questions of the review.	Appendix 1
**Study selection**	9	State the process for selecting studies (i.e., screening, eligibility, included in systematic review, and, if applicable, included in the meta-analysis).		2(Search strategy and selection criteria)
**Data collection process**	10	Describe method of data extraction from reports (e.g., piloted forms, independently, in duplicate) and any processes for obtaining and confirming data from investigators.		3 (Data extraction)
**Data items**	11	List and define all variables for which data were sought (e.g., PICOS, funding sources) and any assumptions and simplifications made.	List and define data items related to equity,where such data were sought (e.g. using PROGRESS-Plus or other criteria, context).	3 (Data extraction)

**Risk of bias in individual studies**	12	Describe methods used for assessing risk of bias of individual studies (including specification of whether this was done at the study or outcome level), and how this information is to be used in any data synthesis.		4-5 (Risk of Bias)
**Summary measures**	13	State the principal summary measures (e.g., risk ratio, difference in means).		3-5 (Meta-Regres.)
**Synthesis of results**	14	Describe the methods of handling data and combining results of studies, if done, including measures of consistency (e.g., I^2) for each meta-analysis.	Describe methods of synthesizing findings on health inequities (e.g. presenting both relative and absolute differences between groups).	3-5 (Meta-Regres.) and appendix 1
**Risk of bias across studies**	15	15. Specify any assessment of risk of bias that may affect the cumulative evidence (e.g., publication bias, selective reporting within studies).		5 (Risk of Bias)
**Additional analyses**	16	Describe methods of additional analyses (e.g., sensitivity or subgroup analyses, meta-regression), if done, indicating which were pre-specified.	Describe methods of <u>additional</u> synthesis approaches related to equity questions, if done, indicating which were pre-specified	3-5 (Meta-Regres.)
**Results**				
**Study selection**	17	Give numbers of studies screened, assessed for eligibility, and included in the review, with reasons for exclusions at each stage, ideally with a flow diagram.		4-5 (Results), Appendix 1 Figure 1 Main figure 1
**Study characteristics**	18	For each study, present characteristics for which data were extracted (e.g., study size, PICOS, follow-up period) and provide the citations.	Present the population characteristics that relate to the equity questions across the relevant PROGRESS-Plus or other factors of interest.	4-5 (Results+ Figure 2 + Appendix
**Risk of bias within studies**	19	Present data on risk of bias of each study and, if available, any outcome level assessment (see item 12).		5 (Study Heterogeneity and Bias)
**Results of individual studies**	20	For all outcomes considered (benefits or harms), present, for each study: (a) simple summary data for each intervention group (b) effect estimates and confidence intervals, ideally with a forest plot.		Supplementary Figure 8
**Synthesis of results**	21	Present results of each meta-analysis done, including confidence intervals and measures of consistency.	Present the results of synthesizing findings on inequities (see 14).	5 (Disaggregated mortality)
**Risk of bias across studies**	22	Present results of any assessment of risk of bias across studies (see Item 15).		5 (Study Heterogeneity and Bias)
**Additional analysis**	23	Give results of additional analyses, if done (e.g., sensitivity or subgroup analyses, meta-regression [see Item 16]).	Give the results of <u>additional</u> synthesis approaches related to equity objectives, if done, (see 16).	Figures 3-5, Appendix 1
**Discussion**				
**Summary of evidence**	24	Summarize the main findings including the strength of evidence for each main outcome; consider their relevance to key groups (e.g., healthcare providers, users, and policy makers).		9-10 (Discussion)
**Limitations**	25	Discuss limitations at study and outcome level (e.g., risk of bias), and at review-level (e.g., incomplete retrieval of identified research, reporting bias).		9-11 (Discussion)
**Conclusions**	26	Provide a general interpretation of the results in the context of other evidence, and implications for future research.	Present extent and limits of applicability to disadvantaged populations of interest and describe the evidence and logic underlying those judgments.	9-11 (Discussion)
	26A		Provide implications for research, practice or policy related to equity where relevant (e.g. types of research needed to address unanswered questions).	9-11(Discussion)
**Funding**				
**Funding**	27	Describe sources of funding for the systematic review and other support (e.g., supply of data); role of funders for the systematic review.		5 (Role of the funding source)

From: Source: Welch V, Petticrew M, Tugwell P, Moher D, O'Neill J, Waters E, White H, and the PRISMA-Equity Bellagio Group. (2012) PRISMA-Equity 2012 Extension: Reporting Guidelines for Systematic Reviews with a Focus on Health Equity. PLoS Med 9(10): e1001333. doi:10.1371/journal.pmed.1001333

List of articles included in systematic review

1. Akbaraly TN, Kivimaki M, Ancelin ML, Barberger-Gateau P, Mura T, Tzourio C, et al. Metabolic syndrome, its components, and mortality in the elderly. J Clin Endocrinol Metab. 2010;95(11):E327-32.
2. Akerkar R, Ebbing M, Sulo G, Ariansen I, Igland J, Tell GS, et al. Educational inequalities in mortality of patients with atrial fibrillation in Norway. Scand Cardiovasc J. 2017;51(2):82-7.
3. Alicandro G, Frova L, Sebastiani G, Boffetta P, La Vecchia C. Differences in education and premature mortality: a record linkage study of over 35 million Italians. Eur J Public Health. 2018;28(2):231-7.
4. Alicandro G, Sebastiani G, Bertuccio P, Zengarini N, Costa G, La Vecchia C, et al. The main causes of death contributing to absolute and relative socio-economic inequality in Italy. Public Health. 2018;164:39-48.
5. Allen JG, Weiss ES, Arnaoutakis GJ, Russell SD, Baumgartner WA, Shah AS, et al. Insurance and education predict long-term survival after orthotopic heart transplantation in the United States. J Heart Lung Transplant. 2012;31(1):52-60.
6. Ambugo EA, Hagen TP. A multilevel analysis of mortality following acute myocardial infarction in Norway: do municipal health services make a difference? BMJ open. 2015;5(11):e008764.
7. Andersson MA. Health returns to education by family socioeconomic origins, 1980-2008: Testing the importance of gender, cohort, and age. SSM Popul Health. 2016;2:549-60.
8. Angel RJ, Angel JL, Diaz Venegas C, Bonazzo C. Shorter stay, longer life: age at migration and mortality among the older Mexican-origin population. J Aging Health. 2010;22(7):914-31.
9. Anstey KJ, Luszcz MA. Mortality risk varies according to gender and change in depressive status in very old adults. Psychosom Med. 2002;64(6):880-8.
10. Appiah D, Baumgartner RN. The Influence of Education and Apolipoprotein epsilon4 on Mortality in Community-Dwelling Elderly Men and Women. J Aging Res. 2018;2018:6037058.
11. Arnau A, Espaulella J, Mendez T, Serrarols M, Canudas J, Formiga F, et al. Lower limb function and 10-year survival in population aged 75 years and older. Fam Pract. 2016;33(1):10-6.
12. Arias E, Johnson NJ, Vera BT. Racial disparities in mortality in the adult hispanic population. SSM Popul Health. 2020 Apr 13;11:100583.
13. Arrich J, Lalouschek W, Mullner M. Influence of socioeconomic status on mortality after stroke: retrospective cohort study. Stroke. 2005;36(2):310-4.
14. Arroyave I, Burdorf A, Cardona D, Avendano M. Socioeconomic inequalities in premature mortality in Colombia, 1998-2007: the double burden of non-communicable diseases and injuries. Prev Med. 2014;64:41-7.
15. Assari S. Perceived Neighborhood Safety Better Predicts Risk of Mortality for Whites than Blacks. J Racial Ethn Health Disparities. 2016;4(5):937-48.
16. Assari S. Number of Chronic Medical Conditions Fully Mediates the Effects of Race on Mortality; 25-Year Follow-Up of a Nationally Representative Sample of Americans. J Racial Ethn Health Disparities. 2017;4(4):623-31.
17. Assari S. Whites but Not Blacks Gain Life Expectancy from Social Contacts. Behav Sci (Basel). 2017;7(4).
18. Assari S. Depressive Symptoms Increase the Risk of Mortality for White but Not Black Older Adults. Healthcare (Basel). 2018;6(2).
19. Assari S. Life Expectancy Gain Due to Employment Status Depends on Race, Gender, Education, and Their Intersections. J Racial Ethn Health Disparities. 2018;5(2):375-86.
20. Assari S, Lankarani MM, Burgard S. Black-white difference in long-term predictive power of self-rated health on all-cause mortality in United States. Ann Epidemiol. 2016;26(2):106-14.
21. Attanasio O, Emmerson C. Differential Mortality in the UK. Rochester, NY; 2001.
22. Auger N, Hamel D, Martinez J, Ross NA. Mitigating effect of immigration on the relation between income inequality and mortality: a prospective study of 2 million Canadians. J Epidemiol Community Health. 2012;66(6):e5.
23. Avlund K, Damsgaard MT, Osler M. Social position and functional decline among non-disabled old men and women. Eur J Public Health. 2004;14(2):212-6.
24. Backlund E, Sorlie PD, Johnson NJ. A comparison of the relationships of education and income with mortality: the National Longitudinal Mortality Study. Soc Sci Med. 1999;49(10):1373-84.
25. Baevre K, Kravdal O. The effects of earlier income variation on mortality: an analysis of Norwegian register data. Popul Stud (Camb). 2014;68(1):81-94.
26. Barclay K, Kolk M. Birth order and mortality: a population-based cohort study. Demography. 2015;52(2):613-39.
27. Barger SD, Broom TW, Esposito MV, Lane TS. Is subjective well-being independently associated with mortality? A 14-year prospective cohort study in a representative sample of 25 139 US men and women. BMJ Open. 2020 Jan 1;10(1):e031776.
28. Bassuk SS, Berkman LF, Amick BC, 3rd. Socioeconomic status and mortality among the elderly: findings from four US communities. Am J Epidemiol. 2002;155(6):520-33.
29. Batty GD, Shipley MJ, Mortensen LH, Boyle SH, Barefoot J, Gronbaek M, et al. IQ in late adolescence/early

adulthood, risk factors in middle age and later all-cause mortality in men: the Vietnam Experience Study. J Epidemiol Community Health. 2008;62(6):522-31.
30. Beckett M. Converging health inequalities in later life--an artifact of mortality selection. J Health Soc Behav. 2000;41(1):106-19.
31. Behrman JR, Sickles R, Taubman P, Yazbeck A. Black-White Mortality Inequalities. Journal of Econometrics. 1991;50(1-2):183-203.
32. Beltrán-Sánchez H, Goldman N, Pebley AR, Morales JF. Calloused hands, shorter life? Occupation and older-age survival in Mexico. Demogr Res. 2020;42:875–900.
33. Bernard SL, Kincade JE, Konrad TR, Arcury TA, Rabiner DJ, Woomert A, et al. Predicting mortality from community surveys of older adults: the importance of self-rated functional ability. J Gerontol B Psychol Sci Soc Sci. 1997;52(3):S155-63.
34. Bernardes GM, Saulo H, Ferreira Santos JL, Teixeira D, Duarte YA, Bof de Andrade F. Effect of education and multimorbidity on mortality among older adults: findings from the health, well-being and ageing cohort study (SABE). Public Health. 2021 Dec 1;201:69–74.
35. Bessudnov A, McKee M, Stuckler D. Inequalities in male mortality by occupational class, perceived status and education in Russia, 1994-2006. Eur J Public Health. 2012;22(3):332-7.
36. Beydoun HA, Beydoun MA, Chen X, Chang JJ, Gamaldo AA, Eid SM, et al. Sex and age differences in the associations between sleep behaviors and all-cause mortality in older adults: results from the National Health and Nutrition Examination Surveys. Sleep Med. 2017;36:141-51.
37. Bihan H, Backholer K, Peeters A, Stevenson CE, Shaw JE, Magliano DJ. Socioeconomic Position and Premature Mortality in the AusDiab Cohort of Australian Adults. Am J Public Health. 2016;106(3):470-7.
38. Bijwaard GE, van Poppel F, Ekamper P, Lumey LH. Gains in Life Expectancy Associated with Higher Education in Men. PLoS One. 2015;10(10):e0141200.
39. Billingsley S. Intragenerational mobility and mortality in Russia: short and longer-term effects. Soc Sci Med. 2012;75(12):2326-36.
40. Blakely T, Atkinson J, Ivory V, Collings S, Wilton J, Howden-Chapman P. No association of neighbourhood volunteerism with mortality in New Zealand: a national multilevel cohort study. Int J Epidemiol. 2006;35(4):981-9.
41. Blazer DG, Hybels CF. What symptoms of depression predict mortality in community-dwelling elders? J Am Geriatr Soc. 2004;52(12):2052-6.
42. Blazer DG, Landerman LR, Hays JC, Grady TA, Havlik R, Corti MC. Blood pressure and mortality risk in older people: comparison between African Americans and whites. J Am Geriatr Soc. 2001;49(4):375-81.
43. Blazer DG, Sachs-Ericsson N, Hybels CF. Perception of unmet basic needs as a predictor of mortality among community-dwelling older adults. Am J Public Health. 2005;95(2):299-304.
44. Bobak M, Murphy M, Rose R, Marmot M. Determinants of adult mortality in Russia: estimates from sibling data. Epidemiology. 2003;14(5):603-11.
45. Bonaccio M, Di Castelnuovo A, Costanzo S, Persichillo M, Donati MB, de Gaetano G, et al. Interaction between education and income on the risk of all-cause mortality: prospective results from the MOLI-SANI study. Int J Public Health. 2016;61(7):765-76.
46. Bond SA, Krueger PM, Rogers RG, Hummer RA. Wealth, race, and mortality. Social Science Quarterly. 2003;84(3):667-84.
47. Bopp M, Braun J, Gutzwiller F, Faeh D, Swiss National Cohort Study G. Health risk or resource? Gradual and independent association between self-rated health and mortality persists over 30 years. PLoS One. 2012;7(2):e30795.
48. Borrell C, Mari-Dell'Olmo M, Rodriguez-Sanz M, Garcia-Olalla P, Cayla JA, Benach J, et al. Socioeconomic position and excess mortality during the heat wave of 2003 in Barcelona. Eur J Epidemiol. 2006;21(9):633-40.
49. Borrell C, Regidor E, Arias LC, Navarro P, Puigpinos R, Dominguez V, et al. Inequalities in mortality according to educational level in two large Southern European cities. Int J Epidemiol. 1999;28(1):58-63.
50. Bosma H, Appels A, Sturmans F, Grabauskas V, Gostautas A. Educational level of spouses and risk of mortality: the WHO Kaunas-Rotterdam Intervention Study (KRIS). Int J Epidemiol. 1995;24(1):119-26.
51. Bosma H, van de Mheen HD, Borsboom GJ, Mackenbach JP. Neighborhood socioeconomic status and all-cause mortality. Am J Epidemiol. 2001;153(4):363-71.
52. Bostock S, Steptoe A. Association between low functional health literacy and mortality in older adults: longitudinal cohort study. BMJ. 2012;344:e1602.
53. Bowling CB, Booth JN, 3rd, Safford MM, Whitson HE, Ritchie CS, Wadley VG, et al. Nondisease-specific problems and all-cause mortality in the REasons for Geographic and Racial Differences in Stroke study. J Am Geriatr Soc. 2013;61(5):739-46.
54. Brajczewski C, Rogucka E. Social class differences in rates of premature mortality among adults in the city of Wroclaw, Poland. Am J Hum Biol. 1993;5(4):461-71.
55. Breschi M, Fornasin A, Manfredini M, Mazzoni S, Pozzi L. Socioeconomic conditions, health and mortality from birth

to adulthood, Alghero 1866-1925. Explorations in Economic History. 2011;48(3):366-75.
56. Brite J. The Association Between Educational Attainment and Mortality: Examining Absolute and Relative Effects by Race/Ethnicity. Ethn Dis. 2022;32(1):1–10.
57. Brown DC, Hayward MD, Montez JK, Hummer RA, Chiu CT, Hidajat MM. The significance of education for mortality compression in the United States. Demography. 2012;49(3):819-40.
58. Brown DC, Lariscy JT, Kalousova L. Comparability of Mortality Estimates from Social Surveys and Vital Statistics Data in the United States. Popul Res Policy Rev. 2019;38(3):371-401.
59. Bucher HC, Ragland DR. Socioeconomic indicators and mortality from coronary heart disease and cancer: a 22-year follow-up of middle-aged men. Am J Public Health. 1995;85(9):1231-6.
60. Bundy JD, Mills KT, He H, LaVeist TA, Ferdinand KC, Chen J, et al. Social determinants of health and premature death among adults in the USA from 1999 to 2018: a national cohort study. Lancet Public Health. 2023 Jun;8(6):e422–31.
61. Cacciani L, Bargagli AM, Cesaroni G, Forastiere F, Agabiti N, Davoli M. Education and Mortality in the Rome Longitudinal Study. PLoS One. 2015;10(9):e0137576.
62. Cambois E. Occupational and educational differentials in mortality in French elderly people: magnitude and trends over recent decades. Demographic research. 2004;2:277-304.
63. Castagné R, Garès V, Karimi M, Chadeau-Hyam M, Vineis P, Delpierre C, et al. Allostatic load and subsequent all-cause mortality: which biological markers drive the relationship? Findings from a UK birth cohort. Eur J Epidemiol. 2018;33:441-58.
64. Cerhan JR, Wallace RB. Change in social ties and subsequent mortality in rural elders. Epidemiology. 1997;8(5):475-81.
65. Chaisson RE, Keruly JC, Moore RD. Race, sex, drug use, and progression of human immunodeficiency virus disease. N Engl J Med. 1995;333(12):751-6.
66. Chen H, Hu H. The relationship and mechanism between education and functional health status transition among older persons in China. BMC Geriatr. 2018;18(1):89.
67. Chen R, Hu Z, Wei L, Wilson K. Socioeconomic status and survival among older adults with dementia and depression. Br J Psychiatry. 2014;204(6):436-40.
68. Chiu CT, Hayward M, Saito Y. A Comparison of Educational Differences on Physical Health, Mortality, and Healthy Life Expectancy in Japan and the United States. J Aging Health. 2016;28(7):1256-78.
69. Choi AI, Weekley CC, Chen SC, Li S, Kurella Tamura M, Norris KC, et al. Association of educational attainment with chronic disease and mortality: the Kidney Early Evaluation Program (KEEP). Am J Kidney Dis. 2011;58(2):228-34.
70. Christ SL, Fleming LE, Lee DJ, Muntaner C, Muennig PA, Caban-Martinez AJ. The effects of a psychosocial dimension of socioeconomic position on survival: occupational prestige and mortality among US working adults. Sociol Health Illn. 2012;34(7):1103-17.
71. Christenson BA, Johnson NE. Educational inequality in adult mortality: an assessment with death certificate data from Michigan. Demography. 1995;32(2):215-29.
72. Christiansen SG. The association between grandparenthood and mortality. Soc Sci Med. 2014;118:89-96.
73. Chu WM, Liao WC, Li CR, Lee SH, Tang YJ, Ho HE, et al. Late-career unemployment and all-cause mortality, functional disability and depression among the older adults in Taiwan: A 12-year population-based cohort study. Arch Gerontol Geriatr. 2016;65:192-8.
74. Coady SA, Johnson NJ, Hakes JK, Sorlie PD. Individual education, area income, and mortality and recurrence of myocardial infarction in a Medicare cohort: the National Longitudinal Mortality Study. BMC Public Health. 2014;14:705.
75. Comstock GW, Tonascia JA. Education and mortality in Washington County, Maryland. J Health Soc Behav. 1978;18(1):54-61.
76. Contador I, Stern Y, Bermejo-Pareja F, Sanchez-Ferro A, Benito-Leon J. Is educational attainment associated with increased risk of mortality in people with dementia? A population-based study. Current Alzheimer Research. 2017;14(5):571-6.
77. Conway BN, Elasy TA, May ME, Blot WJ. Mortality by race among low-income adults with early-onset insulin-treated diabetes. Diabetes Care. 2013;36(10):3107-12.
78. Cunningham WE, Hays RD, Duan N, Andersen R, Nakazono TT, Bozzette SA, et al. The effect of socioeconomic status on the survival of people receiving care for HIV infection in the United States. J Health Care Poor Underserved. 2005;16(4):655-76.
79. Cutler DM, Lange F, Meara E, Richards-Shubik S, Ruhm CJ. Rising educational gradients in mortality: the role of behavioral risk factors. J Health Econ. 2011;30(6):1174-87.
80. d'Errico A, Ricceri F, Stringhini S, Carmeli C, Kivimaki M, Bartley M, et al. Socioeconomic indicators in epidemiologic research: A practical example from the LIFEPATH study. PLoS One. 2017;12(5):e0178071.
81. Dahl E, Elstad JI, Hofoss D, Martin-Mollard M. For whom is income inequality most harmful? A multi-level analysis

of income inequality and mortality in Norway. Soc Sci Med. 2006;63(10):2562-74.
82. Dalen JD, Huijts T, Krokstad S, Eikemo TA. Are there educational differences in the association between self-rated health and mortality in Norway? The HUNT Study. Scandinavian Journal of Public Health. 2012;40(7):641-7.
83. Dalsgaard EM, Skriver MV, Sandbaek A, Vestergaard M. Socioeconomic position, type 2 diabetes and long-term risk of death. PLoS One. 2015;10(5):e0124829.
84. Davila EP, Christ SL, Caban-Martinez AJ, Lee DJ, Arheart KL, LeBlanc WG, et al. Young adults, mortality, and employment. J Occup Environ Med. 2010;52(5):501-4.
85. Davis D, Cooper R, Terrera GM, Hardy R, Richards M, Kuh D. Verbal memory and search speed in early midlife are associated with mortality over 25 years' follow-up, independently of health status and early life factors: a British birth cohort study. Int J Epidemiol. 2016;45(4):1216-25.
86. De Grande H, Vandenheede H, Deboosere P. Trends in young-adult mortality between the 1990s and the 2000s in urban and non-urban areas in Belgium: the role of a changing educational composition in overall mortality decline. Health Place. 2014;30:61-9.
87. De Grande H, Vandenheede H, Deboosere P. Educational Inequalities in the Transition to Adulthood in Belgium: The Impact of Intergenerational Mobility on Young-Adult Mortality in 2001-2009. PLoS One. 2015;10(12):e0142104.
88. De Grande H, Vandenheede H, Deboosere P. Educational inequalities in young-adult mortality between the 1990s and the 2000s: regional differences in Belgium. Archives of public health. 2015;73(1):1-12.
89. De Moortel D, Hagedoorn P, Vanroelen C, Gadeyne S. Employment status and mortality in the context of high and low regional unemployment levels in Belgium (2001-2011): A test of the social norm hypothesis across educational levels. PLoS One. 2018;13(2):e0192526.
90. Deary IJ, Batty GD, Pattie A, Gale CR. More intelligent, more dependable children live longer: a 55-year longitudinal study of a representative sample of the Scottish nation. Psychol Sci. 2008;19(9):874-80.
91. Deary IJ, Der G. Reaction time explains IQ's association with death. Psychol Sci. 2005;16(1):64-9.
92. Deaton AS, Paxson C. Mortality, education, income, and inequality among American cohorts. Themes in the Economics of Aging: University of Chicago Press; 2001. p. 129-70.
93. Demakakos P, Biddulph JP, de Oliveira C, Tsakos G, Marmot MG. Subjective social status and mortality: the English Longitudinal Study of Ageing. Eur J Epidemiol. 2018;33(8):729-39.
94. Denisova I. Adult mortality in Russia. Economics of Transition. 2010;18(2):333-63.
95. Denney JT, Rogers RG, Hummer RA, Pampel FC. Education inequality in mortality: The age and gender specific mediating effects of cigarette smoking. Soc Sci Res. 2010;39(4):662-73.
96. Diaz AA, Come CE, Mannino DM, Pinto-Plata V, Divo MJ, Bigelow C, et al. Obstructive lung disease in Mexican Americans and non-Hispanic whites: an analysis of diagnosis and survival in the National Health and Nutritional Examination Survey III Follow-up Study. Chest. 2014;145(2):282-9.
97. Doblhammer G. Reproductive history and mortality later in life: a comparative study of England and Wales and Austria. Popul Stud (Camb). 2000;54(2):169-76.
98. Doblhammer G, Rau R, Kytir J. Trends in educational and occupational differentials in all-cause mortality in Austria between 1981/82 and 1991/92. Wien Klin Wochenschr. 2005;117(13-14):468-79.
99. Doblhammer-reiter G. [Social inequalities in death: on the extent of socioeconomic differences in mortality in Austria]. Demogr Inf. 1995:71-81, 162-3.
100. Dong X, Simon MA, Fulmer T, Mendes de Leon CF, Hebert LE, Beck T, et al. A prospective population-based study of differences in elder self-neglect and mortality between black and white older adults. J Gerontol A Biol Sci Med Sci. 2011;66(6):695-704.
101. Doniec K, Stefler D, Murphy M, Gugushvili A, McKee M, Marmot M, et al. Education and mortality in three Eastern European populations: findings from the PrivMort retrospective cohort study. Eur J Public Health. 2019;29(3):549-54.
102. Doornbos G, Kromhout D. Educational level and mortality in a 32-year follow-up study of 18-year-old men in The Netherlands. Int J Epidemiol. 1990;19(2):374-9.
103. Dowd JB, Zajacova A. Does the predictive power of self-rated health for subsequent mortality risk vary by socioeconomic status in the US? Int J Epidemiol. 2007;36(6):1214-21.
104. Dray-Spira R, Gary-Webb TL, Brancati FL. Educational disparities in mortality among adults with diabetes in the U.S. Diabetes Care. 2010;33(6):1200-5.
105. Dupre ME, Franzese AT, Parrado EA. Religious attendance and mortality: implications for the black-white mortality crossover. Demography. 2006;43(1):141-64.
106. Dupre ME, Liu G, Gu D. Predictors of longevity: evidence from the oldest old in China. Am J Public Health. 2008;98(7):1203-8.
107. Dupre ME, Silberberg M, Willis JM, Feinglos MN. Education, glucose control, and mortality risks among U.S. older adults with diabetes. Diabetes Res Clin Pract. 2015;107(3):392-9.
108. Edwards R. Who is hurt by procyclical mortality? Soc Sci Med. 2008;67(12):2051-8.
109. Ekamper P, van Poppel F, Stein AD, Lumey LH. Independent and additive association of prenatal famine exposure and

intermediary life conditions with adult mortality between age 18-63 years. Soc Sci Med. 2014;119:232-9.
110. Elo IT, Martikainen P, Myrskyla M. Socioeconomic status across the life course and all-cause and cause-specific mortality in Finland. Soc Sci Med. 2014;119:198-206.
111. Elo IT, Martikainen P, Smith KP. Socioeconomic differentials in mortality in Finland and the United States: the role of education and income. European Journal of Population. 2006;22(2):179-203.
112. Elo IT, Mehta N, Preston S. The Contribution of Weight Status to Black-White Differences in Mortality. Biodemography Soc Biol. 2017;63(3):206-20.
113. Elo IT, Preston SH. Educational differentials in mortality: United States, 1979–1985. Soc Sci Med. 1996;42(1):47-57.
114. Elstad JI. Does the socioeconomic context explain both mortality and income inequality? Prospective register-based study of Norwegian regions. International journal for equity in health. 2011;10(1):1-11.
115. Elstad JI, Overbye E, Dahl E. Prospective register-based study of the impact of immigration on educational inequalities in mortality in Norway. BMC Public Health. 2015;15:364.
116. Enroth L, Raitanen J, Hervonen A, Nosraty L, Jylhä M. Is socioeconomic status a predictor of mortality in nonagenarians? The vitality 90+ study. Age Ageing. 2014;44(1):123-9.
117. Ericsson M, Pedersen NL, Johansson ALV, Fors S, Dahl Aslan AK. Life-course socioeconomic differences and social mobility in preventable and non-preventable mortality: a study of Swedish twins. Int J Epidemiol. 2019;48(5):1701-9.
118. Erikson R, Torssander J. Clerics die, doctors survive: a note on death risks among highly educated professionals. Scand J Public Health. 2009;37(3):227-31.
119. Eriksson JG, Kajantie E, Lampl M, Osmond C, Barker DJ. Markers of biological fitness as predictors of all-cause mortality. Ann Med. 2013;45(2):156-61.
120. Eschbach K, Ostir GV, Patel KV, Markides KS, Goodwin JS. Neighborhood context and mortality among older Mexican Americans: is there a barrio advantage? Am J Public Health. 2004;94(10):1807-12.
121. Espinoza SE, Jung I, Hazuda H. The Hispanic paradox and predictors of mortality in an aging biethnic cohort of Mexican Americans and European Americans: the san antonio longitudinal study of aging. J Am Geriatr Soc. 2013;61(9):1522-9.
122. Evans-Polce RJ, Staff J, Maggs JL. Alcohol abstention in early adulthood and premature mortality: Do early life factors, social support, and health explain this association? Soc Sci Med. 2016;163:71-9.
123. Faeh D, Bopp M, Swiss National Cohort Study G. Educational inequalities in mortality and associated risk factors: German--versus French-speaking Switzerland. BMC Public Health. 2010;10:567.
124. Faggiano F, Lemma P, Costa G, Gnavi R, Pagnanelli F. Cancer mortality by educational level in Italy. Cancer Causes Control. 1995;6(4):311-20.
125. Fan Y, He D. Self-rated health, socioeconomic status and all-cause mortality in Chinese middle-aged and elderly adults. Sci Rep. 2022 Jun 3;12(1):9309.
126. Fantahun M, Berhane Y, Hogberg U, Wall S, Byass P. Ageing of a rural Ethiopian population: who are the survivors? Public Health. 2009;123(4):326-30.
127. Feinglass J, Lin S, Thompson J, Sudano J, Dunlop D, Song J, et al. Baseline health, socioeconomic status, and 10-year mortality among older middle-aged Americans: findings from the Health and Retirement Study, 1992 2002. J Gerontol B Psychol Sci Soc Sci. 2007;62(4):S209-17.
128. Feinstein L, Douglas CE, Stebbins RC, Pawelec G, Simanek AM, Aiello AE. Does cytomegalovirus infection contribute to socioeconomic disparities in all-cause mortality? Mech Ageing Dev. 2016;158:53-61.
129. Feldman JJ, Makuc DM, Kleinman JC, Cornoni-Huntley J. National trends in educational differentials in mortality. Am J Epidemiol. 1989;129(5):919-33.
130. Fenelon A, Chinn JJ, Anderson RN. A comprehensive analysis of the mortality experience of hispanic subgroups in the United States: Variation by age, country of origin, and nativity. SSM Popul Health. 2017;3:245-54.
131. Feng Z, Jones K, Wang WW. An exploratory discrete-time multilevel analysis of the effect of social support on the survival of elderly people in China. Soc Sci Med. 2015;130:181-9.
132. Ferri CP, Acosta D, Guerra M, Huang Y, Llibre-Rodriguez JJ, Salas A, et al. Socioeconomic factors and all cause and cause-specific mortality among older people in Latin America, India, and China: a population-based cohort study. PLoS Med. 2012;9(2):e1001179.
133. Fluharty ME, Hardy R, Ploubidis G, Pongiglione B, Bann D. Socioeconomic inequalities across life and premature mortality from 1971 to 2016: findings from three British birth cohorts born in 1946, 1958 and 1970. J Epidemiol Community Health. 2021 Feb;75(2):193–6.
134. Fors S, Modin B, Koupil I, Vagero D. Socioeconomic inequalities in circulatory and all-cause mortality after retirement: the impact of mid-life income and old-age pension. Evidence from the Uppsala Birth Cohort Study. J Epidemiol Community Health. 2012;66(7):e16.
135. Forssas E, Manderbacka K, Arffman M, Keskimaki I. Socio-economic predictors of mortality among diabetic people. Eur J Public Health. 2012;22(3):305-10.
136. Fowler M, Kenzik K, Al-Obaidi M, Harmon C, Giri S, Arora S, et al. Rural-urban disparities in mortality and geriatric

assessment among older adults with cancer: The cancer & aging resilience evaluation (CARE) registry. Journal of geriatric oncology. 2023 Apr 21;14:101505.
137. Franks P, Clancy CM, Gold MR. Health insurance and mortality. Evidence from a national cohort. JAMA. 1993;270(6):737-41.
138. Franks P, Gold MR, Fiscella K. Sociodemographics, self-rated health, and mortality in the US. Soc Sci Med. 2003;56(12):2505-14.
139. Franzon K, Byberg L, Sjogren P, Zethelius B, Cederholm T, Kilander L. Predictors of Independent Aging and Survival: A 16-Year Follow-Up Report in Octogenarian Men. J Am Geriatr Soc. 2017;65(9):1953-60.
140. Frederiksen BL, Dalton SO, Osler M, Steding-Jessen M, de Nully Brown P. Socioeconomic position, treatment, and survival of non-Hodgkin lymphoma in Denmark--a nationwide study. Br J Cancer. 2012;106(5):988-95.
141. Friedman EM, Mare RD. The schooling of offspring and the survival of parents. Demography. 2014;51(4):1271-93.
142. Fujino Y, Tamakoshi A, Iso H, Inaba Y, Kubo T, Ide R, et al. A nationwide cohort study of educational background and major causes of death among the elderly population in Japan. Prev Med. 2005;40(4):444-51.
143. Gallo V, Mackenbach JP, Ezzati M, Menvielle G, Kunst AE, Rohrmann S, et al. Social inequalities and mortality in Europe--results from a large multi-national cohort. PLoS One. 2012;7(7):e39013.
144. Geerlings MI, Deeg DJ, Penninx BW, Schmand B, Jonker C, Bouter LM, et al. Cognitive reserve and mortality in dementia: the role of cognition, functional ability and depression. Psychol Med. 1999;29(5):1219-26.
145. Gerdtham UG, Johannesson M. Absolute income, relative income, income inequality, and mortality. J Hum Resour. 2004;39(1):228-47.
146. Geyer S, Hemstrom O, Peter R, Vagero D. Education, income, and occupational class cannot be used interchangeably in social epidemiology. Empirical evidence against a common practice. J Epidemiol Community Health. 2006;60(9):804-10.
147. Gilmore DR, Whitfield KE, Thorpe Jr RJ. Is there a difference in all-cause mortality between non-Hispanic Black and non-Hispanic White men with the same level of education? Analyses using the 2000–2011 National Health Interview Surveys. American journal of men's health. 2019;13(1):1557988319827793.
148. Giordano GN, Mewes J, Miething A. Trust and all-cause mortality: a multilevel study of US General Social Survey data (1978-2010). J Epidemiol Community Health. 2019;73(1):50-5.
149. Glei DA, Lee C, Weinstein M. Assessment of Mortality Disparities by Wealth Relative to Other Measures of Socioeconomic Status Among US Adults. JAMA Netw Open. 2022 Apr 1;5(4):e226547.
150. Gnavi R, Canova C, Picariello R, Tessari R, Giorda C, Simonato L, et al. Mortality, incidence of cardiovascular diseases, and educational level among the diabetic and non-diabetic populations in two large Italian cities. Diabetes Res Clin Pract. 2011;92(2):205-12.
151. Gnavi R, Petrelli A, Demaria M, Spadea T, Carta Q, Costa G. Mortality and educational level among diabetic and non-diabetic population in the Turin Longitudinal Study: a 9-year follow-up. Int J Epidemiol. 2004;33(4):864-71.
152. Gomes MM, Turra CM, Figoli MG, Duarte YA, Lebrao ML. Past and present: conditions of life during childhood and mortality of older adults. Rev Saude Publica. 2015;49.
153. Gomez-Olive FX, Thorogood M, Kandala NB, Tigbe W, Kahn K, Tollman S, et al. Sleep problems and mortality in rural South Africa: novel evidence from a low-resource setting. Sleep Med. 2014;15(1):56-63.
154. Gonzalez-Bautista E, Manrique-Espinoza B, Avila-Funes JA, Naidoo N, Kowal P, Chatterji S, et al. Social determinants of health and frailty are associated with all-cause mortality in older adults. Salud Publica Mex. 2019;61(5):582-90.
155. Grabowski DC, Ellis JE. High body mass index does not predict mortality in older people: analysis of the Longitudinal Study of Aging. J Am Geriatr Soc. 2001;49(7):968-79.
156. Granlund D, Chuc NT, Phuc HD, Lindholm L. Inequality in mortality in Vietnam during a period of rapid transition. Soc Sci Med. 2010;70(2):232-9.
157. Griffin FR, Mode NA, Ejiogu N, Zonderman AB, Evans MK. Frailty in a racially and socioeconomically diverse sample of middle-aged Americans in Baltimore. PLoS One. 2018;13(4):e0195637.
158. Grigoriev P, Scholz R, Shkolnikov VM. Socioeconomic differences in mortality among 27 million economically active Germans: a cross-sectional analysis of the German Pension Fund data. BMJ Open. 2019;9(10):e028001.
159. Grundy EM, Tomassini C. Marital history, health and mortality among older men and women in England and Wales. BMC Public Health. 2010;10:554.
160. Green KM, Doherty EE, Bugbee BA. Can Early Disadvantage Be Overcome? A Life Course Approach to Understanding How Disadvantage, Education, and Social Integration Impact Mortality into Middle Adulthood Among a Black American Cohort. Prev Sci. 2023 Jul;24(5):829–40.
161. Gu D, Zhang Z, Zeng Y. Access to healthcare services makes a difference in healthy longevity among older Chinese adults. Soc Sci Med. 2009;68(2):210-9.
162. Guillot M, Khlat M, Wallace M. Adult mortality among second-generation immigrants in France: Results from a nationally representative record linkage study. Demogr Res. 2019;40:1603-44.

163. Guo Y, Chan KST, Chan CH, Chang Q, Lee RS, Yip PSF. Combined effects of individual and neighbourhood socioeconomic status on older adults' mortality: a retrospective follow-up study in Hong Kong. BMJ Open. 2021 Apr 27;11(4):e043192.
164. Hadden WC, Rockswold PD. Increasing differential mortality by educational attainment in adults in the United States. Int J Health Serv. 2008;38(1):47-61.
165. Hales S, Blakely T, Woodward A. Air pollution and mortality in New Zealand: cohort study. J Epidemiol Community Health. 2012;66(5):468-73.
166. Han BH, Blaum CS, Ferris RE, Min LC, Lee PG. Older Adults Reporting More Diabetes Mellitus Care Have Greater 9-Year Survival. J Am Geriatr Soc. 2015;63(12):2455-62.
167. Hardarson T, Gardarsdottir M, Gudmundsson KT, Thorgeirsson G, Sigvaldason H, Sigfusson N. The relationship between educational level and mortality. The Reykjavik Study. J Intern Med. 2001;249(6):495-502.
168. Hardy MA, Reyes AM. The Longevity Legacy of World War II: The Intersection of GI Status and Mortality. Gerontologist. 2016;56(1):104-14.
169. Hayward MD, Gorman BK. The long arm of childhood: the influence of early-life social conditions on men's mortality. Demography. 2004;41(1):87-107.
170. Hayward MD, Hummer RA, Sasson I. Trends and group differences in the association between educational attainment and U.S. adult mortality: implications for understanding education's causal influence. Soc Sci Med. 2015;127:8-18.
171. Hayward MD, Pienta AM, McLaughlin DK. Inequality in men's mortality: the socioeconomic status gradient and geographic context. J Health Soc Behav. 1997;38(4):313-30.
172. Hayward MD, Warner DF, Crimmins EM. Does longer life mean better health? Not for native-born Mexican Americans in the Health and Retirement Survey. The health of aging Hispanics: The Mexican-origin population. 2007:85-95.
173. Henretta JC. Lifetime marital history and mortality after age 50. J Aging Health. 2010;22(8):1198-212.
174. Heslop P, Smith GD, Macleod J, Hart C. The socioeconomic position of employed women, risk factors and mortality. Soc Sci Med. 2001;53(4):477-85.
175. Himmelstein KEW, Lawrence JA, Jahn JL, Ceasar JN, Morse M, Bassett MT, et al. Association Between Racial Wealth Inequities and Racial Disparities in Longevity Among US Adults and Role of Reparations Payments, 1992 to 2018. JAMA Netw Open. 2022 Nov 1;5(11):e2240519.
176. Hirokawa K, Tsutusmi A, Kayaba K. Impacts of educational level and employment status on mortality for Japanese women and men: the Jichi Medical School cohort study. Eur J Epidemiol. 2006;21(9):641-51.
177. Hirve S, Juvekar S, Sambhudas S, Lele P, Blomstedt Y, Wall S, et al. Does self-rated health predict death in adults aged 50 years and above in India? Evidence from a rural population under health and demographic surveillance. Int J Epidemiol. 2012;41(6):1719-27.
178. Ho SC. Health and social predictors of mortality in an elderly Chinese cohort. Am J Epidemiol. 1991;133(9):907-21.
179. Hoffmann R. Do socioeconomic mortality differences decrease with rising age? Demographic Research. 2005;13:35-62.
180. Hoffmann R. Socioeconomic inequalities in old-age mortality: a comparison of Denmark and the USA. Soc Sci Med. 2011;72(12):1986-92.
181. Holmes JS, Driscoll AK, Heron M. Mortality among US-born and immigrant Hispanics in the US: effects of nativity, duration of residence, and age at immigration. Int J Public Health. 2015;60(5):609-17.
182. Honjo K, Iso H, Fukuda Y, Nishi N, Nakaya T, Fujino Y, et al. Influence of municipal- and individual-level socioeconomic conditions on mortality in Japan. Int J Behav Med. 2014;21(5):737-49.
183. Honjo K, Iso H, Ikeda A, Fujino Y, Tamakoshi A, Group JS. Employment situation and risk of death among middle-aged Japanese women. J Epidemiol Community Health. 2015;69(10):1012-7.
184. Howard JT, Sparks PJ. The Effects of Allostatic Load on Racial/Ethnic Mortality Differences in the United States. Population Research and Policy Review. 2016;35(4):421-43.
185. Hsu H-C. Does social participation by the elderly reduce mortality and cognitive impairment? Aging Ment Health. 2007;11(6):699-707.
186. Huang C, Elo IT. Mortality of the oldest old Chinese: the role of early-life nutritional status, socio-economic conditions, and sibling sex-composition. Popul Stud (Camb). 2009;63(1):7-20.
187. Huie SAB, Hummer RA, Rogers RG. Individual and contextual risks of death among race and ethnic groups in the United States. J Health Soc Behav. 2002;43(3):359-81.
188. Huisman M, Kunst AE, Andersen O, Bopp M, Borgan JK, Borrell C, et al. Socioeconomic inequalities in mortality among elderly people in 11 European populations. J Epidemiol Community Health. 2004;58(6):468-75.
189. Huisman M, van Lenthe F, Mackenbach J. The predictive ability of self-assessed health for mortality in different educational groups. Int J Epidemiol. 2007;36(6):1207-13.
190. Hummer RA, Benjamins MR, Rogers RG. Racial and ethnic disparities in health and mortality among the US elderly population. Critical perspectives on racial and ethnic differences in health in late life. 2004:53-94.

191. Hummer RA, Chinn JJ. RACE/ETHNICITY AND U.S. ADULT MORTALITY: Progress, Prospects, and New Analyses. Du Bois Rev. 2011;8(1):5-24.
192. Hummer RA, Lariscy JT. Educational Attainment and Adult Mortality. In: Rogers RG, Crimmins EM, editors. International Handbook of Adult Mortality. International Handbooks of Population. Dordrecht: Springer Netherlands; 2011. p. 241-61.
193. Hummer RA, Rogers RG, Amir SH, Forbes D, Frisbie WP. Adult mortality differentials among Hispanic subgroups and non-Hispanic whites. Social Science Quarterly. 2000;81(1):459-76.
194. Hummer RA, Rogers RG, Nam CB, Ellison CG. Religious involvement and US adult mortality. Demography. 1999;36(2):273-85.
195. Hummer RA, Rogers RG, Nam CB, LeClere FB. Race/ethnicity, nativity, and US adult mortality. Social Science Quarterly. 1999;80(1):136-53.
196. Hurt LS, Ronsmans C, Saha S. Effects of education and other socioeconomic factors on middle age mortality in rural Bangladesh. J Epidemiol Community Health. 2004;58(4):315-20.
197. Hypertension Detection and Follow-up Program Cooperative Group. Educational Level and 5-Year All-Cause Mortality in the Hypertension Detection and Follow-up Program. Hypertension. 1987;9(6):641-6.
198. Idler EL, Angel RJ. Self-rated health and mortality in the NHANES-I Epidemiologic Follow-up Study. Am J Public Health. 1990;80(4):446-52.
199. Irma TE, Greg LD. Educational differences in cause-specific mortality in the United States. Finnish Yearbook of Population Research. 2002:37-54.
200. Ishizaki T, Kai I, Imanaka Y. Self-rated health and social role as predictors for 6-year total mortality among a non-disabled older Japanese population. Arch Gerontol Geriatr. 2006;42(1):91-9.
201. Ito S, Takachi R, Inoue M, Kurahashi N, Iwasaki M, Sasazuki S, et al. Education in relation to incidence of and mortality from cancer and cardiovascular disease in Japan. Eur J Public Health. 2008;18(5):466-72.
202. Iwasa H, Kawaai C, Gondo Y, Inagaki H, Suzuki T. Subjective well-being as a predictor of all-cause mortality among middle-aged and elderly people living in an urban Japanese community: A seven-year prospective cohort study. Geriatrics & Gerontology International. 2006;6(4):216-22.
203. Iwasaki M, Otani T, Ohta A, Yosiaki S, Kuroiwa M, Suzuki S. Rural-urban differences in sociodemographic, social network and lifestyle factors related to mortality of middle-aged Japanese men from the Komo-Ise cohort study. J Epidemiol. 2002;12(2):93-104.
204. Izquierdo C, Oviedo M, Ruiz L, Sintes X, Vera I, Nebot M, et al. Influence of socioeconomic status on community-acquired pneumonia outcomes in elderly patients requiring hospitalization: a multicenter observational study. BMC Public Health. 2010;10:421.
205. Jaffe DH, Eisenbach Z, Neumark YD, Manor O. Does living in a religiously affiliated neighborhood lower mortality? Ann Epidemiol. 2005;15(10):804-10.
206. Jaffe DH, Eisenbach Z, Neumark YD, Manor O. Does one's own and one's spouse's education affect overall and cause-specific mortality in the elderly? Int J Epidemiol. 2005;34(6):1409-16.
207. Jaffe DH, Neumark YD, Eisenbach Z, Manor O. Educational inequalities in mortality among Israeli Jews: changes over time in a dynamic population. Health Place. 2008;14(2):287-98.
208. Jarrin I, Lumbreras B, Ferreros I, Perez-Hoyos S, Hurtado I, Hernandez-Aguado I. Effect of education on overall and cause-specific mortality in injecting drug users, according to HIV and introduction of HAART. Int J Epidemiol. 2007;36(1):187-94.
209. Jatrana S, Blakely T. Socio-economic inequalities in mortality persist into old age in New Zealand: study of all 65 years plus, 2001-04. Ageing & Society. 2014;34(6):911-29.
210. Jatrana S, Dayal S, Richardson K, Blakely T. Socio-economic inequalities in mortality for Asian people: New Zealand Census-Mortality Study, 1996-2004. J Popul Res. 2018;35(4):417-33.
211. Jemal A, Thun MJ, Ward EE, Henley SJ, Cokkinides VE, Murray TE. Mortality from leading causes by education and race in the United States, 2001. Am J Prev Med. 2008;34(1):1-8.
212. Jemal A, Ward E, Anderson RN, Murray T, Thun MJ. Widening of socioeconomic inequalities in U.S. death rates, 1993-2001. PLoS One. 2008;3(5):e2181.
213. Jiang N. Adult Children's Education and Later-Life Health of Parents in China: The Intergenerational Effects of Human Capital Investment. Social Indicators Research. 2019;145(1):257-78.
214. John PS, Montgomery P. Does self-rated health predict death in older adults with depressive symptoms? Canadian Journal on Aging/La Revue canadienne du vieillissement. 2012;31(1):49-54.
215. Jokela M, Batty GD, Deary IJ, Gale CR, Kivimaki M. Low childhood IQ and early adult mortality: the role of explanatory factors in the 1958 British Birth Cohort. Pediatrics. 2009;124(3):e380-8.
216. Jorgensen TS, Osler M, Angquist LH, Zimmermann E, Christensen GT, Sorensen TI. The U-shaped association of body mass index with mortality: Influence of the traits height, intelligence, and education. Obesity (Silver Spring). 2016;24(10):2240-7.

217. Jotheeswaran AT, Williams JD, Prince MJ. Predictors of mortality among elderly people living in a south Indian urban community; a 10/66 Dementia Research Group prospective population-based cohort study. BMC Public Health. 2010;10:366.
218. Kalediene R, Prochorskas R, Sauliune S. Socio-economic mortality inequalities in Lithuania during 2001-2009: the record linkage study. Public Health. 2015;129(12):1645-51.
219. Kalist DE, Peng Y. Does education matter? Major League Baseball players and longevity. Death Stud. 2007;31(7):653-70.
220. Kallan J. Effects of sociodemographic variables on adult mortality in the United States: comparisons by sex, age, and cause of death. Soc Biol. 1997;44(1-2):136-47.
221. Kaplan RM, Howard VJ, Safford MM, Howard G. Educational attainment and longevity: results from the REGARDS U.S. national cohort study of blacks and whites. Ann Epidemiol. 2015;25(5):323-8.
222. Kessler M, Thumé E, Marmot M, Macinko J, Facchini LA, Nedel FB, et al. Family Health Strategy, Primary Health Care, and Social Inequalities in Mortality Among Older Adults in Bagé, Southern Brazil. Am J Public Health. 2021 May;111(5):927–36.
223. Kesztenbaum L, Rosenthal JL. The health cost of living in a city: The case of France at the end of the 19th century. Explorations in Economic History. 2011;48(2):207-25.
224. Keyes CL, Simoes EJ. To flourish or not: positive mental health and all-cause mortality. Am J Public Health. 2012;102(11):2164-72.
225. Khan N, Javed Z, Acquah I, Hagan K, Khan M, Valero-Elizondo J, et al. Low educational attainment is associated with higher all-cause and cardiovascular mortality in the United States adult population. BMC Public Health. 2023 May 16;23(1):900.
226. Khang YH. Relationship between childhood socio-economic position and mortality risk in adult males of the Korea Labour and Income Panel Study (KLIPS). Public Health. 2006;120(8):724-31.
227. Khang YH, Kim HR. Explaining socioeconomic inequality in mortality among South Koreans: an examination of multiple pathways in a nationally representative longitudinal study. Int J Epidemiol. 2005;34(3):630-7.
228. Khang YH, Kim HR. Relationship of education, occupation, and income with mortality in a representative longitudinal study of South Korea. Eur J Epidemiol. 2005;20(3):217-20.
229. Khang YH, Kim HR. [Socioeconomic mortality inequality in Korea: mortality follow-up of the 1998 National Health and Nutrition Examination Survey (NHANES) data]. J Prev Med Public Health. 2006;39(2):115-22.
230. Khang YH, Kim HR. Socioeconomic Inequality in mortality using 12-year follow-up data from nationally representative surveys in South Korea. Int J Equity Health. 2016;15:51.
231. Khang YH, Lynch JW, Kaplan GA. Health inequalities in Korea: age- and sex-specific educational differences in the 10 leading causes of death. Int J Epidemiol. 2004;33(2):299-308.
232. Khang YH, Lynch JW, Yang S, Harper S, Yun SC, Jung-Choi K, et al. The contribution of material, psychosocial, and behavioral factors in explaining educational and occupational mortality inequalities in a nationally representative sample of South Koreans: relative and absolute perspectives. Soc Sci Med. 2009;68(5):858-66.
233. Khlat M, Wallace M, Guillot M. Divergent mortality patterns for second generation men of North-African and South-European origin in France: Role of labour force participation. SSM Popul Health. 2019;9:100447.
234. Kilander L, Berglund L, Boberg M, Vessby B, Lithell H. Education, lifestyle factors and mortality from cardiovascular disease and cancer. A 25-year follow-up of Swedish 50-year-old men. Int J Epidemiol. 2001;30(5):1119-26.
235. Kim GR, Jee SH, Pikhart H. Role of allostatic load and health behaviours in explaining socioeconomic disparities in mortality: a structural equation modelling approach. J Epidemiol Community Health. 2018;72(6):545-51.
236. Kim J, Smith TW, Kang JH. Religious Affiliation, Religious Service Attendance, and Mortality. J Relig Health. 2015;54(6):2052-72.
237. Kim JM, Son NH, Park EC, Nam CM, Kim TH, Cho WH. The relationship between changes in employment status and mortality risk based on the Korea Labor and Income Panel Study (2003-2008). Asia Pac J Public Health. 2015;27(2):NP993-1001.
238. Kimbro LB, Mangione CM, Steers WN, Duru OK, McEwen L, Karter A, et al. Depression and all-cause mortality in persons with diabetes mellitus: Are older adults at higher risk? Results from the translating research into action for diabetes study. J Am Geriatr Soc. 2014;62(6):1017-22.
239. Kitagawa EM, Hauser PM. Differential Mortality in the United States: A Study in Socioeconomic Epidemiology. In: Differential Mortality in the United States. Harvard University Press; 2013.
240. Kiuila O, Mieszkowski P. The effects of income, education and age on health. Health Econ. 2007;16(8):781-98.
241. Kivimaki M, Gunnell D, Lawlor DA, Davey Smith G, Pentti J, Virtanen M, et al. Social inequalities in antidepressant treatment and mortality: a longitudinal register study. Psychol Med. 2007;37(3):373-82.
242. Klinthall M, Lindstrom M. Migration and health: a study of effects of early life experiences and current socio-economic situation on mortality of immigrants in Sweden. Ethn Health. 2011;16(6):601-23.
243. Koch E, Romero T, Romero C, Akel C, Manriquez L, Paredes M, et al. Impact of education, income and chronic

disease risk factors on mortality of adults: does ‘a pauper-rich paradox’exist in Latin American societies? Public Health. 2010;124(1):39-48.
244. Koch E, Romero T, Romero CX, Aguilera H, Paredes M, Vargas M, et al. Early life and adult socioeconomic influences on mortality risk: preliminary report of a 'pauper rich' paradox in a Chilean adult cohort. Ann Epidemiol. 2010;20(6):487-92.
245. Koch K, Norgaard M, Schonheyder HC, Thomsen RW, Sogaard M, Danish Collaborative Bacteremia N. Effect of socioeconomic status on mortality after bacteremia in working-age patients. A Danish population-based cohort study. PLoS One. 2013;8(7):e70082.
246. Komura T, Kondo N, Bhatt K, Inoue K. Association Between Educational Status and Mortality According to Diabetes Status Among US Adults. Mayo Clin Proc Innov Qual Outcomes. 2023 Aug;7(4):203–11.
247. Kolodziej H, Lopuszanska M, Bielicki T, Jankowska EA. Social inequality in premature mortality among Polish urban adults during economic transition. Am J Hum Biol. 2007;19(6):878-85.
248. Konrath S, Fuhrel-Forbis A, Lou A, Brown S. Motives for volunteering are associated with mortality risk in older adults. Health Psychol. 2012;31(1):87-96.
249. Koskinen S, Martelin T. Why are socioeconomic mortality differences smaller among women than among men? Soc Sci Med. 1994;38(10):1385-96.
250. Kposowa AJ. Race/Ethnicity and Income Disparities in US Adult Mortality. Br J Med Med Res. 2015;5(2):191.
251. Kposowa AJ, Bideshi D. Reassessing the Sources of Racial and Ethnic Disparities in US Adult Mortality. Western Journal of Black Studies. 2006;30(1).
252. Krause JS, Saunders LL. Socioeconomic and behavioral risk factors for mortality: do risk factors observed after spinal cord injury parallel those from the general USA population? Spinal Cord. 2012;50(8):609-13.
253. Krause N, Hayward RD. Self-Forgiveness and Mortality in Late Life. Social Indicators Research. 2013;111(1):361-73.
254. Kravdal O. A cancer survival model that takes sociodemographic variations in "normal" mortality into account: comparison with other models. J Epidemiol Community Health. 2002;56(4):309-18.
255. Kravdal O. A fixed-effects multilevel analysis of how community family structure affects individual mortality in Norway. Demography. 2007;44(3):519-37.
256. Kravdal O. Mortality effects of average education in current and earlier municipality of residence among internal migrants, net of their own education. Soc Sci Med. 2009;69(10):1484-92.
257. Kravdal O. Mortality effects of average education: a multilevel study of small neighbourhoods in rural and urban areas in Norway. Int J Equity Health. 2009;8:41.
258. Kravdal O. Large and Growing Social Inequality in Mortality in Norway: The Combined Importance of Marital Status and Own and Spouse's Education. Population and Development Review. 2017;43(4):645-+.
259. Kravdal Ø. A broader perspective on education and mortality: are we influenced by other people's education? Soc Sci Med. 2008;66(3):620-36.
260. Kravdal Ø, Grundy E, Keenan K. The increasing mortality advantage of the married: The role played by education. Demographic research. 2018;38:471-512.
261. Kroger H, Hoffmann R, Tarkiainen L, Martikainen P. Comparing Observed and Unobserved Components of Childhood: Evidence From Finnish Register Data on Midlife Mortality From Siblings and Their Parents. Demography. 2018;55(1):295-318.
262. Krueger PM, Chang VW. Being poor and coping with stress: health behaviors and the risk of death. Am J Public Health. 2008;98(5):889-96.
263. Krueger PM, Dehry IA, Chang VW. The Economic Value of Education for Longer Lives and Reduced Disability. Milbank Q. 2019;97(1):48-73.
264. Krueger PM, Rogers RG, Hummer RA, LeClere FB, Huie SAB. Socioeconomic status and age: The effect of income sources and portfolios on US adult mortality. Sociological Forum. 2003;18(3):465-82.
265. Krueger PM, Tran MK, Hummer RA, Chang VW. Mortality Attributable to Low Levels of Education in the United States. PLoS One. 2015;10(7):e0131809.
266. Kruger DJ, Nesse RM. An evolutionary life-history framework for understanding sex differences in human mortality rates. Hum Nat. 2006;17(1):74-97.
267. Kuh D, Shah I, Richards M, Mishra G, Wadsworth M, Hardy R. Do childhood cognitive ability or smoking behaviour explain the influence of lifetime socio-economic conditions on premature adult mortality in a British post war birth cohort? Soc Sci Med. 2009;68(9):1565-73.
268. Kulhanova I, Hoffmann R, Eikemo TA, Menvielle G, Mackenbach JP. Educational inequalities in mortality by cause of death: first national data for the Netherlands. Int J Public Health. 2014;59(5):687-96.
269. Kulhanova I, Hoffmann R, Judge K, Looman CW, Eikemo TA, Bopp M, et al. Assessing the potential impact of increased participation in higher education on mortality: evidence from 21 European populations. Soc Sci Med. 2014;117:142-9.
270. Kunst AE, Bos V, Mackenbach JP. Monitoring socio-economic inequalities in health in the European Union: guidelines

and illustrations. The Netherlands: Department of Public Health, Erasmus University Rotterdam. 2001.
271. Kunst AE, Groenhof F, Mackenbach JP. Mortality by occupational class among men 30-64 years in 11 European countries. EU Working Group on Socioeconomic Inequalities in Health. Soc Sci Med. 1998;46(11):1459-76.
272. Lallo C, Raitano M. Life expectancy inequalities in the elderly by socioeconomic status: evidence from Italy. Popul Health Metr. 2018;16(1):7.
273. Landes SD. The influence of SES on the mortality differential between adults with and without intellectual disability. Conference Papers -- American Sociological Association. 2014:1-28.
274. Landes SD. The Association between Education and Mortality for Adults with Intellectual Disability. J Health Soc Behav. 2017;58(1):70-85.
275. Landman GWD, Kleefstra N, van Hateren KJJ, Gans ROB, Bilo HJG, Groenier KH. Educational disparities in mortality among patients with type 2 diabetes in the Netherlands (ZODIAC-23). Neth J Med. 2013;71(2):76-80.
276. Lantz PM, Golberstein E, House JS, Morenoff J. Socioeconomic and behavioral risk factors for mortality in a national 19-year prospective study of U.S. adults. Soc Sci Med. 2010;70(10):1558-66.
277. Lantz PM, House JS, Lepkowski JM, Williams DR, Mero RP, Chen J. Socioeconomic factors, health behaviors, and mortality: results from a nationally representative prospective study of US adults. JAMA. 1998;279(21):1703-8.
278. Lara E, Moreno-Agostino D, Martín-María N, Miret M, Rico-Uribe LA, Olaya B, et al. Exploring the effect of loneliness on all-cause mortality: Are there differences between older adults and younger and middle-aged adults? Soc Sci Med. 2020 Aug;258:113087.
279. Lariscy JT, Hummer RA, Hayward MD. Hispanic older adult mortality in the United States: new estimates and an assessment of factors shaping the Hispanic paradox. Demography. 2015;52(1):1-14.
280. Lawrence EM, Rogers RG, Zajacova A. Educational Attainment and Mortality in the United States: Effects of Degrees, Years of Schooling, and Certification. Popul Res Policy Rev. 2016;35(4):501-25.
281. Lawrence EM, Rogers RG, Zajacova A, Wadsworth T. Marital Happiness, Marital Status, Health, and Longevity. Journal of Happiness Studies. 2019;20(5):1539-61.
282. LeClere FB, Rogers RG, Peters KD. Ethnicity and mortality in the United States: Individual and community correlates. Soc Forces. 1997;76(1):169-98.
283. Lee H, Singh GK. Contributions of Socioeconomic, Demographic, and Behavioral Risk Factors to All-Cause Mortality Disparities by Psychological Distress in the United States: A Blinder-Oaxaca Decomposition Analysis of Longitudinal Data. Int J MCH AIDS. 2022;11(1):e533.
284. Lee MC, Huang N. Changes in self-perceived economic satisfaction and mortality at old ages: evidence from a survey of middle-aged and elderly adults in Taiwan. Soc Sci Med. 2015;130:1-8.
285. Leinsalu M, Stirbu I, Vagero D, Kalediene R, Kovacs K, Wojtyniak B, et al. Educational inequalities in mortality in four Eastern European countries: divergence in trends during the post-communist transition from 1990 to 2000. Int J Epidemiol. 2009;38(2):512-25.
286. Leinsalu M, Vagero D, Kunst AE. Estonia 1989-2000: enormous increase in mortality differences by education. Int J Epidemiol. 2003;32(6):1081-7.
287. Levchenko Y, Fenelon A. How Tooth Loss Disrupts the Education Gradient in Mortality Risk among US-Born and Foreign-Born Adults. Popul Res Policy Rev. 2022 Jun 1;41(3):811–23.
288. Lewden C, Raffi F, Cuzin L, Cailleton V, Vilde JL, Chene G, et al. Factors associated with mortality in human immunodeficiency virus type 1-infected adults initiating protease inhibitor-containing therapy: role of education level and of early transaminase level elevation (APROCO-ANRS EP11 study). The Antiproteases Cohorte Agence Nationale de Recherches sur le SIDA EP 11 study. J Infect Dis. 2002;186(5):710-4.
289. Liang J, Bennett J, Krause N, Kobayashi E, Kim H, Brown JW, et al. Old age mortality in Japan: does the socioeconomic gradient interact with gender and age? The Journals of Gerontology Series B: Psychological Sciences and Social Sciences. 2002;57(5):S294-S307.
290. Liang J, McCarthy JF, Jain A, Krause N, Bennett JM, Gu S. Socioeconomic gradient in old age mortality in Wuhan, China. J Gerontol B Psychol Sci Soc Sci. 2000;55(4):S222-33.
291. Liao MY, Yeh CJ, Lee SH, Liao CC, Lee MC. Association of providing/receiving support on the mortality of older adults with different living arrangements in Taiwan: a longitudinal study on ageing. Ageing Soc. 2018;38(10):2082-96.
292. Lim D, Kong KA, Lee HA, Lee WK, Park SH, Baik SJ, et al. The population attributable fraction of low education for mortality in South Korea with improvement in educational attainment and no improvement in mortality inequalities. BMC Public Health. 2015;15:313.
293. Liu H, Jiang Q, Feldman MW. Widowhood and mortality risk of older people in rural China: do gender and living arrangement make a difference? Ageing & Society. 2020;40(9):1939-55.
294. Liu K, Cedres LB, Stamler J, Dyer A, Stamler R, Nanas S, et al. Relationship of education to major risk factors and death from coronary heart disease, cardiovascular diseases and all causes, Findings of three Chicago epidemiologic studies. Circulation. 1982;66(6):1308-14.
295. Liu X, Hermalin AI, Chuang YL. The effect of education on mortality among older Taiwanese and its pathways. J

Gerontol B Psychol Sci Soc Sci. 1998;53(2):S71-82.
296. Long JA, Ickovics JR, Gill TM, Horwitz RI. Social class and mortality in older women. J Clin Epidemiol. 2002;55(10):952-8.
297. Lund Jensen N, Pedersen HS, Vestergaard M, Mercer SW, Glumer C, Prior A. The impact of socioeconomic status and multimorbidity on mortality: a population-based cohort study. Clin Epidemiol. 2017;9:279-89.
298. Lundborg P, Lyttkens CH, Nystedt P. The Effect of Schooling on Mortality: New Evidence From 50,000 Swedish Twins. Demography. 2016;53(4):1135-68.
299. Lundström H, Nilsson Å, Qvist J. Dödlighet efter utbildning, boende och civilstånd : perioden 1986 - 2003: Stockholm : Statistiska Centralbyrån; 2004.
300. Luo J, Hendryx M, Wang F. Mortality disparities between Black and White Americans mediated by income and health behaviors. SSM Popul Health. 2021 Dec 29;17:101019.
301. Luo W, Xie Y. Socio-economic disparities in mortality among the elderly in China. Popul Stud (Camb). 2014;68(3):305-20.
302. Luo Y, Zhang Z, Gu D. Education and mortality among older adults in China. Soc Sci Med. 2015;127:134-42.
303. Laaksonen M, Martikainen P, Nihtila E, Rahkonen O, Lahelma E. Home ownership and mortality: a register-based follow-up study of 300,000 Finns. J Epidemiol Community Health. 2008;62(4):293-7.
304. Laaksonen M, Talala K, Martelin T, Rahkonen O, Roos E, Helakorpi S, et al. Health behaviours as explanations for educational level differences in cardiovascular and all-cause mortality: a follow-up of 60 000 men and women over 23 years. Eur J Public Health. 2008;18(1):38-43.
305. Laaksonen M, Tarkiainen L, Martikainen P. Housing wealth and mortality: A register linkage study of the Finnish population. Soc Sci Med. 2009;69(5):754-60.
306. Ma J, Xu J, Anderson RN, Jemal A. Widening educational disparities in premature death rates in twenty six states in the United States, 1993-2007. PLoS One. 2012;7(7):e41560.
307. Ma S, Cutter J, Tan CE, Chew SK, Tai ES. Associations of diabetes mellitus and ethnicity with mortality in a multiethnic Asian population: data from the 1992 Singapore National Health Survey. Am J Epidemiol. 2003;158(6):543-52.
308. Mackenbach JP. Inequalities in health in The Netherlands according to age, gender, marital status, level of education, degree of urbanization, and region. The European Journal of Public Health. 1993;3(2):112-8.
309. Mackenbach JP, Bos V, Andersen O, Cardano M, Costa G, Harding S, et al. Widening socioeconomic inequalities in mortality in six Western European countries. Int J Epidemiol. 2003;32(5):830-7.
310. Mackenbach JP, Kunst AE, Groenhof F, Borgan JK, Costa G, Faggiano F, et al. Socioeconomic inequalities in mortality among women and among men: an international study. Am J Public Health. 1999;89(12):1800-6.
311. Macleod J, Smith GD, Metcalfe C, Hart C. Is subjective social status a more important determinant of health than objective social status? Evidence from a prospective observational study of Scottish men. Soc Sci Med. 2005;61(9):1916-29.
312. MacNeil-Vroomen J, Schulz R, Doyle M, Murphy TE, Ives DG, Monin JK. Time-varying social support and time to death in the cardiovascular health study. Health Psychol. 2018;37(11):1000-5.
313. Madsen M, Andersen A-MN, Christensen K, Andersen PK, Osler M. Does educational status impact adult mortality in Denmark? A twin approach. Am J Epidemiol. 2010;172(2):225-34.
314. Majer IM, Nusselder WJ, Mackenbach JP, Kunst AE. Socioeconomic inequalities in life and health expectancies around official retirement age in 10 Western-European countries. J Epidemiol Community Health. 2011;65(11):972-9.
315. Malyutina S, Bobak M, Simonova G, Gafarov V, Nikitin Y, Marmot M. Education, marital status, and total and cardiovascular mortality in Novosibirsk, Russia: a prospective cohort study. Ann Epidemiol. 2004;14(4):244-9.
316. Manor O, Eisenbach Z. Mortality after spousal loss: are there socio-demographic differences? Soc Sci Med. 2003;56(2):405-13.
317. Manor O, Eisenbach Z, Israeli A, Friedlander Y. Mortality differentials among women: the Israel Longitudinal Mortality Study. Soc Sci Med. 2000;51(8):1175-88.
318. Manor O, Eisenbach Z, Peritz E, Friedlander Y. Mortality differentials among Israeli men. Am J Public Health. 1999;89(12):1807-13.
319. Mare RD. Socioeconomic careers and differential mortality among older men in the United States: Center for Demography and Ecology; 1987.
320. Marinacci C, Grippo F, Pappagallo M, Sebastiani G, Demaria M, Vittori P, et al. Social inequalities in total and cause-specific mortality of a sample of the Italian population, from 1999 to 2007. Eur J Public Health. 2013;23(4):582-7.
321. Marlow EC, Jemal A, Thomson B, Wiese D, Zhao J, Siegel RL, et al. Mortality by Education Before and During the COVID-19 Pandemic, U.S., 2017–2020. Am J Prev Med. 2023 Jan;64(1):105–16.
322. Martelin T. Mortality by indicators of socioeconomic status among the Finnish elderly. Soc Sci Med. 1994;38(9):1257-78.
323. Martelin T, Koskinen S, Valkonen T. Sociodemographic mortality differences among the oldest old in Finland. J

Gerontol B Psychol Sci Soc Sci. 1998;53(2):S83-90.
324. Martikainen P. Socioeconomic mortality differentials in men and women according to own and spouse's characteristics in Finland. Sociol Health Illn. 1995;17(3):353-75.
325. Martikainen P, Blomgren J, Valkonen T. Change in the total and independent effects of education and occupational social class on mortality: analyses of all Finnish men and women in the period 1971-2000. J Epidemiol Community Health. 2007;61(6):499-505.
326. Martikainen P, Kauppinen TM, Valkonen T. Effects of the characteristics of neighbourhoods and the characteristics of people on cause specific mortality: a register based follow up study of 252,000 men. J Epidemiol Community Health. 2003;57(3):210-7.
327. Martikainen P, Nihtila E, Moustgaard H. The effects of socioeconomic status and health on transitions in living arrangements and mortality: a longitudinal analysis of elderly Finnish men and women from 1997 to 2002. J Gerontol B Psychol Sci Soc Sci. 2008;63(2):S99-109.
328. Martin Rdos S, Martin LC, Franco RJ, Barretti P, Caramori JC, Castro JH, et al. Ventricular hypertrophy and cardiovascular mortality in hemodialysis patients with low educational level. Arq Bras Cardiol. 2012;98(1):52-61.
329. Martinez C, Regidor E, Sanchez E, Pascual C, de la Fuente L. Heterogeneity by age in educational inequalities in cause-specific mortality in women in the Region of Madrid. J Epidemiol Community Health. 2009;63(10):832-8.
330. McDonough P, Duncan GJ, Williams D, House J. Income dynamics and adult mortality in the United States, 1972 through 1989. Am J Public Health. 1997;87(9):1476-83.
331. McDonough P, Williams DR, House JS, Duncan GJ. Gender and the socioeconomic gradient in mortality. J Health Soc Behav. 1999;40(1):17-31.
332. McFadden E, Luben R, Wareham N, Bingham S, Khaw KT. Occupational social class, educational level, smoking and body mass index, and cause-specific mortality in men and women: a prospective study in the European Prospective Investigation of Cancer and Nutrition in Norfolk (EPIC-Norfolk) cohort. Eur J Epidemiol. 2008;23(8):511-22.
333. McKinnon SA, Hummer RA. Education and mortality risk among Hispanic adults in the United States. The health of aging Hispanics: The Mexican-origin population. 2007:65-84.
334. Meara ER, Richards S, Cutler DM. The gap gets bigger: changes in mortality and life expectancy, by education, 1981-2000. Health Aff (Millwood). 2008;27(2):350-60.
335. Meghir C, Palme M, Simeonova E. Education, health and mortality: Evidence from a social experiment. NBER Working Paper Series. 2012:17932.
336. Meghir C, Palme M, Simeonova E. Education and Mortality: Evidence from a Social Experiment. American Economic Journal-Applied Economics. 2018;10(2):234-56.
337. Mehta N, Preston S. Are major behavioral and sociodemographic risk factors for mortality additive or multiplicative in their effects? Soc Sci Med. 2016;154:93-9.
338. Mehta NK, House JS, Elliott MR. Dynamics of health behaviours and socioeconomic differences in mortality in the USA. J Epidemiol Community Health. 2015;69(5):416-22.
339. Menchik PL. Economic status as a determinant of mortality among black and white older men: does poverty kill? Population Studies. 1993;47(3):427-36.
340. Menvielle G, Chastang JF, Luce D, Leclerc A, Groupe E. [Changing social disparities and mortality in France (1968-1996): cause of death analysis by educational level]. Rev Epidemiol Sante Publique. 2007;55(2):97-105.
341. Mercado C, Beckles G, Cheng Y, Bullard KM, Saydah S, Gregg E, et al. Trends and socioeconomic disparities in all-cause mortality among adults with diagnosed diabetes by race/ethnicity: a population-based cohort study - USA, 1997-2015. BMJ Open. 2021 May 4;11(5):e044158.
342. Miething A. A matter of perception: exploring the role of income satisfaction in the income-mortality relationship in German survey data 1995-2010. Soc Sci Med. 2013;99:72-9.
343. Min-Ah L, Kim J, Jeong-han K. Is living with both parents in childhood beneficial? Effects on mortality by later marital status. Conference Papers -- American Sociological Association. 2015:1-32.
344. Minagawa Y, Saito Y. Active social participation and mortality risk among older people in Japan: results from a nationally representative sample. Res Aging. 2015;37(5):481-99.
345. Miqueleiz E, Lostao L, Reques L, Santos JM, Calle ME, Regidor E. [Inequalities in Total Mortality and by Cause of Death According to the Level of Education in Navarra: findings from a Longitudinal Study from 2001 to 2008]. Rev Esp Salud Publica. 2015;89(3):295-306.
346. Moe JO, Steingrimsdottir OA, Strand BH, Groholt EK, Naess O. Trends in educational inequalities in old age mortality in Norway 1961-2009: a prospective register based population study. BMC Public Health. 2012;12:911.
347. Molla MT, Madans JH, Wagener DK. Differentials in adult mortality and activity limitation by years of education in the United States at the end of the 1990s. Population and Development Review. 2004;30(4):625-+.
348. Montez JK, Barnes K. The benefits of educational attainment for US adult mortality: Are they contingent on the broader environment? Population Research and Policy Review. 2016;35:73-100.
349. Montez JK, Berkman LF. Trends in the educational gradient of mortality among US adults aged 45 to 84 years:

bringing regional context into the explanation. Am J Public Health. 2014;104(1):e82-90.
350. Montez JK, Hayward MD. Cumulative childhood adversity, educational attainment, and active life expectancy among U.S. adults. Demography. 2014;51(2):413-35.
351. Montez JK, Hayward MD, Brown DC, Hummer RA. Why is the educational gradient of mortality steeper for men? Journals of Gerontology Series B: Psychological Sciences and Social Sciences. 2009;64(5):625-34.
352. Montez JK, Martikainen P, Remes H, Avendano M. Work-Family Context and the Longevity Disadvantage of US Women. Soc Forces. 2015;93(4):1567-97.
353. Montez JK, Zajacova A. Explaining the widening education gap in mortality among U.S. white women. J Health Soc Behav. 2013;54(2):166-82.
354. Montez JK, Zajacova A. Trends in mortality risk by education level and cause of death among US White women from 1986 to 2006. Am J Public Health. 2013;103(3):473-9.
355. Montez JK, Zajacova A, Hayward MD. Explaining inequalities in women's mortality between US States. SSM-population health. 2016;2:561-71.
356. Montez JK, Zajacova A, Hayward MD, Woolf SH, Chapman D, Beckfield J. Educational disparities in adult mortality across US states: How do they differ, and have they changed since the mid-1980s? Demography. 2019;56(2):621-44.
357. Moreno X, Albala C, Lera L, Sanchez H, Fuentes-Garcia A, Dangour AD. The role of gender in the association between self-rated health and mortality among older adults in Santiago, Chile: A cohort study. PLoS One. 2017;12(7):e0181317.
358. Mortensen LH, Torssander J. Family of origin and educational inequalities in mortality: Results from 1.7 million Swedish siblings. SSM Popul Health. 2017;3:192-200.
359. Morton PM, Ferraro KF. Early Social Origins of Biological Risks for Men and Women in Later Life. J Health Soc Behav. 2020 Dec;61(4):503–22.
360. Mostafa G, van Ginneken JK. Trends in and determinants of mortality in the elderly population of Matlab, Bangladesh. Soc Sci Med. 2000;50(6):763-71.
361. Moulton BE, Sherkat DE. Specifying the Effects of Religious Participation and Educational Attainment on Mortality Risk for Us Adults. Sociological Spectrum. 2012;32(1):1-19.
362. Muhuri PK, Gfroerer JC. Mortality associated with illegal drug use among adults in the United States. Am J Drug Alcohol Abuse. 2011;37(3):155-64.
363. Murphy M, Bobak M, Nicholson A, Rose R, Marmot M. The widening gap in mortality by educational level in the Russian Federation, 1980-2001. Am J Public Health. 2006;96(7):1293-9.
364. Mustard CA, Bielecky A, Etches J, Wilkins R, Tjepkema M, Amick BC, et al. Mortality following unemployment in Canada, 1991-2001. BMC Public Health. 2013;13:441.
365. Myrskyla M, Fenelon A. Maternal age and offspring adult health: evidence from the health and retirement study. Demography. 2012;49(4):1231-57.
366. Naess O, Claussen B, Thelle DS, Smith GD. Four indicators of socioeconomic position: relative ranking across causes of death. Scand J Public Health. 2005;33(3):215-21.
367. Naess O, Hoff DA, Lawlor D, Mortensen LH. Education and adult cause-specific mortality--examining the impact of family factors shared by 871 367 Norwegian siblings. Int J Epidemiol. 2012;41(6):1683-91; author reply 91-3.
368. Naess O, Piro FN, Nafstad P, Smith GD, Leyland AH. Air pollution, social deprivation, and mortality: a multilevel cohort study. Epidemiology. 2007;18(6):686-94.
369. Nandakumar A, Anantha N, Venugopal TC, Sankaranarayanan R, Thimmasetty K, Dhar M. Survival in breast cancer: a population-based study in Bangalore, India. Int J Cancer. 1995;60(5):593-6.
370. Nandi A, Glymour MM, Subramanian SV. Association among socioeconomic status, health behaviors, and all-cause mortality in the United States. Epidemiology. 2014;25(2):170-7.
371. Ng N, Hakimi M, Santosa A, Byass P, Wilopo SA, Wall S. Is self-rated health an independent index for mortality among older people in Indonesia? PLoS One. 2012;7(4):e35308.
372. Ng-Mak DS, Dohrenwend BP, Abraido-Lanza AF, Turner JB. A further analysis of race differences in the National Longitudinal Mortality Study. Am J Public Health. 1999;89(11):1748-51.
373. Nikoi CA, Odimegwu C. The association between socioeconomic status and adult mortality in rural Kwazulu-Natal, South Africa. Oman Med J. 2013;28(2):102.
374. Nishi A, Kawachi I, Shirai K, Hirai H, Jeong S, Kondo K. Sex/gender and socioeconomic differences in the predictive ability of self-rated health for mortality. PLoS One. 2012;7(1):e30179.
375. Noale M, Minicuci N, Bardage C, Gindin J, Nikula S, Pluijm S, et al. Predictors of mortality: an international comparison of socio-demographic and health characteristics from six longitudinal studies on aging: the CLESA project. Exp Gerontol. 2005;40(1-2):89-99.
376. Nogimura A, Noguchi T, Otani T, Kamiji K, Yasuoka M, Watanabe R, et al. Chronic obstructive pulmonary disease and the mortality risk in male older adults: Role of socioeconomic factors. Arch Gerontol Geriatr. 2022;102:104741.
377. Notara V, Panagiotakos DB, Kogias Y, Stravopodis P, Antonoulas A, Zombolos S, et al. The impact of educational

status on 10-year (2004-2014) cardiovascular disease prognosis and all-cause mortality among acute coronary syndrome patients in the Greek acute coronary syndrome (GREECS) longitudinal study. Journal of Preventive Medicine and Public Health. 2016;49(4):220.
378. Nybo H, Petersen HC, Gaist D, Jeune B, Andersen K, McGue M, et al. Predictors of mortality in 2,249 nonagenarians--the Danish 1905-Cohort Survey. J Am Geriatr Soc. 2003;51(10):1365-73.
379. Oh HJ. An exploration of the influence of household poverty spells on mortality risk. Journal of Marriage and Family. 2001;63(1):224-34.
380. Ojagbemi A, Bello T, Luo Z, Gureje O. Living Conditions, Low Socioeconomic Position, and Mortality in the Ibadan Study of Aging. J Gerontol B Psychol Sci Soc Sci. 2017;72(4):646-55.
381. Okely JA, Weiss A, Gale CR. The interaction between stress and positive affect in predicting mortality. J Psychosom Res. 2017;100:53-60.
382. Osler M, Prescott E. Educational level as a contextual and proximate determinant of all cause mortality in Danish adults. J Epidemiol Community Health. 2003;57(4):266-9.
383. Ostergren O. Growing gaps: The importance of income and family for educational inequalities in mortality among Swedish men and women 1990-2009. Scand J Public Health. 2015;43(6):563-70.
384. Ostergren O. Educational inequalities in mortality are larger at low levels of income: A register-based study on premature mortality among 2.3 million Swedes, 2006-2009. SSM Popul Health. 2018;5:122-8.
385. Oude Groeniger J, Kamphuis CB, Mackenbach JP, van Lenthe FJ. Repeatedly measured material and behavioral factors changed the explanation of socioeconomic inequalities in all-cause mortality. J Clin Epidemiol. 2017;91:137-45.
386. Pacheco AG, Veloso VG, Nunes EP, Ribeiro S, Guimaraes MR, Lourenco MC, et al. Tuberculosis is associated with non-tuberculosis-related deaths among HIV/AIDS patients in Rio de Janeiro. Int J Tuberc Lung Dis. 2014;18(12):1473-8.
387. Palloni A, Arias E. Paradox lost: explaining the Hispanic adult mortality advantage. Demography. 2004;41(3):385-415.
388. Pampel FC, Rogers RG. Socioeconomic status, smoking, and health: a test of competing theories of cumulative advantage. J Health Soc Behav. 2004;45(3):306-21.
389. Pantell M, Rehkopf D, Jutte D, Syme SL, Balmes J, Adler N. Social isolation: a predictor of mortality comparable to traditional clinical risk factors. Am J Public Health. 2013;103(11):2056-62.
390. Pappas G, Queen S, Hadden W, Fisher G. The increasing disparity in mortality between socioeconomic groups in the United States, 1960 and 1986. N Engl J Med. 1993;329(2):103-9.
391. Patterson AC. Does the mortality risk of social isolation depend upon socioeconomic factors? J Health Psychol. 2016;21(10):2420-33.
392. Paul R, Rashmi. Risk factors and clustering of mortality among older adults in the India Human Development Survey. Sci Rep. 2022 Apr 22;12(1):6644.
393. Pednekar MS, Gupta R, Gupta PC. Illiteracy, low educational status, and cardiovascular mortality in India. BMC Public Health. 2011;11:567.
394. Pensola T, Martikainen P. Life-course experiences and mortality by adult social class among young men. Soc Sci Med. 2004;58(11):2149-70.
395. Perak AM, Ning H, Khan SS, Bundy JD, Allen NB, Lewis CE, et al. Associations of Late Adolescent or Young Adult Cardiovascular Health With Premature Cardiovascular Disease and Mortality. J Am Coll Cardiol. 2020 Dec 8;76(23):2695–707.
396. Perlman F, Bobak M. Socioeconomic and behavioral determinants of mortality in posttransition Russia: a prospective population study. Ann Epidemiol. 2008;18(2):92-100.
397. Perlman F, Bobak M. Determinants of self rated health and mortality in Russia–are they the same? International journal for equity in health. 2008;7:1-8.
398. Pikala M, Burzyn Ska M, Pikala R, Bryla M, Maniecka-Bryla I. Educational inequalities in premature mortality in Poland, 2002-2011: a population-based cross-sectional study. BMJ Open. 2016;6(9):e011501.
399. Pinto JM, Wroblewski KE, Huisingh-Scheetz M, Correia C, Lopez KJ, Chen RC, et al. Global Sensory Impairment Predicts Morbidity and Mortality in Older U.S. Adults. J Am Geriatr Soc. 2017;65(12):2587-95.
400. Pratipanawatr T, Rawdaree P, Chetthakul T, Bunnag P, Ngarmukos C, Benjasuratwong Y, et al. Differences in Mortality by Education Level among Patients in Diabetic Registry for Thailand. Southeast Asian J Trop Med Public Health. 2015;46(1):125-32.
401. Preston SH, Hill ME, Drevenstedt GL. Childhood conditions that predict survival to advanced ages among African-Americans. Soc Sci Med. 1998;47(9):1231-46.
402. Pridemore WA, Tomkins S, Eckhardt K, Kiryanov N, Saburova L. A case-control analysis of socio-economic and marital status differentials in alcohol- and non-alcohol-related mortality among working-age Russian males. Eur J Public Health. 2010;20(5):569-75.
403. Protopopescu C, Raffi F, Spire B, Hardel L, Michelet C, Cheneau C, et al. Twelve-year mortality in HIV-infected patients receiving antiretroviral therapy: the role of social vulnerability. The ANRS CO8 APROCO-COPILOTE cohort.

Antivir Ther. 2015;20(7):763-72.
404. Pu C, Tang GJ, Huang N, Chou YJ. Predictive power of self-rated health for subsequent mortality risk during old age: analysis of data from a nationally representative survey of elderly adults in Taiwan. J Epidemiol. 2011;21(4):278-84.
405. Pudaric S, Sundquist J, Johansson SE. Country of birth, instrumental activities of daily living, self-rated health and mortality: a Swedish population-based survey of people aged 55-74. Soc Sci Med. 2003;56(12):2493-503.
406. Puts MT, Lips P, Deeg DJ. Sex differences in the risk of frailty for mortality independent of disability and chronic diseases. J Am Geriatr Soc. 2005;53(1):40-7.
407. Qureshi AI, Suri MF, Saad M, Hopkins LN. Educational attainment and risk of stroke and myocardial infarction. Med Sci Monit. 2003;9(11):CR466-73.
408. Rahman MM, Howard G, Qian J, Garza K, Abebe A, Hansen R. Disparities in all-cause mortality with potentially inappropriate medication use: Analysis of the REasons for Geographic And Racial Differences in Stroke (REGARDS) study. J Am Pharm Assoc (2003). 2021;61(1):44–52.
409. Rask K, O'Malley E, Druss B. Impact of socioeconomic, behavioral and clinical risk factors on mortality. J Public Health (Oxf). 2009;31(2):231-8.
410. Rasmussen JN, Rasmussen S, Gislason GH, Buch P, Abildstrom SZ, Kober L, et al. Mortality after acute myocardial infarction according to income and education. J Epidemiol Community Health. 2006;60(4):351-6.
411. Rau R, Doblhammer G, Canudas-Romo V, Zhen Z. Cause-of-Death Contributions to Educational Inequalities in Mortality in Austria between 1981/1982 and 1991/1992: Les contributions des causes de deces aux inegalites de mortalite par niveau d'education en Autriche entre 1981/1982 et 1991/1992. Eur J Popul. 2008;24(3):265-86.
412. Rawshani A, Svensson AM, Rosengren A, Eliasson B, Gudbjornsdottir S. Impact of Socioeconomic Status on Cardiovascular Disease and Mortality in 24,947 Individuals With Type 1 Diabetes. Diabetes Care. 2015;38(8):1518-27.
413. Rawshani A, Svensson AM, Zethelius B, Eliasson B, Rosengren A, Gudbjornsdottir S. Association Between Socioeconomic Status and Mortality, Cardiovascular Disease, and Cancer in Patients With Type 2 Diabetes. JAMA Intern Med. 2016;176(8):1146-54.
414. Razzaque A, Carmichael GA, Streatfield PK. Adult mortality in Matlab, Bangladesh: levels, trends, socio-demographic differentials and causes of death. Asian Population Studies. 2009;5(1):85-100.
415. Regidor E, Calle ME, Domínguez V, Navarro P. Mortalidad según características sociales y económicas: Estudio de Mortalidad de la Comunidad Autónoma de Madrid. Med Clin (Barc). 2001;116(19):726-31.
416. Regidor E, de la Fuente L, Calle ME, Navarro P, Dominguez V. Unusually strong association between education and mortality in young adults in a community with a high rate of injection-drug users. Eur J Public Health. 2003;13(4):334-9.
417. Regidor E, Guallar-Castillon P, Gutierrez-Fisac JL, Banegas JR, Rodriguez-Artalejo F. Socioeconomic variation in the magnitude of the association between self-rated health and mortality. Ann Epidemiol. 2010;20(5):395-400.
418. Regidor E, Kunst AE, Rodriguez-Artalejo F, Mackenbach JP. Small socio-economic differences in mortality in Spanish older people. Eur J Public Health. 2012;22(1):80-5.
419. Regidor E, Reques L, Belza MJ, Kunst AE, Mackenbach JP, de la Fuente L. Education and mortality in Spain: a national study supports local findings. Int J Public Health. 2016;61(1):139-45.
420. Regidor E, Ronda E, Martinez D, Calle ME, Navarro P, Dominguez V. Occupational social class and mortality in a population of men economically active: the contribution of education and employment situation. Eur J Epidemiol. 2005;20(6):501-8.
421. Regidor E, Vallejo F, Giraldez-Garcia C, Ortega P, Santos JM, Astasio P, et al. Low mortality in the poorest areas of Spain: adults residing in provinces with lower per capita income have the lowest mortality. Eur J Epidemiol. 2015;30(8):637-48.
422. Regidor E, Vallejo F, Reques L, Cea L, Miqueleiz E, Barrio G. Area-level socioeconomic context, total mortality and cause-specific mortality in Spain: Heterogeneous findings depending on the level of geographic aggregation. Soc Sci Med. 2015;141:142-50.
423. Rehkopf DH, Haughton LT, Chen JT, Waterman PD, Subramanian SV, Krieger N. Monitoring socioeconomic disparities in death: comparing individual-level education and area-based socioeconomic measures. Am J Public Health. 2006;96(12):2135-8.
424. Reile R, Leinsalu M. Ethnic variation in self-rated health-mortality association: Results from a 17-year follow-up study in Estonia. Medicina (Kaunas). 2017;53(2):114-21.
425. Reither EN, Peppard PE, Remington PL, Kindig DA. Increasing educational disparities in premature adult mortality, Wisconsin, 1990-2000. WMJ. 2006;105(7):38-41.
426. Renard F, Devleesschauwer B, Gadeyne S, Tafforeau J, Deboosere P. Educational inequalities in premature mortality by region in the Belgian population in the 2000s. Arch Public Health. 2017;75:44.
427. Rendall MS, Weden MM, Favreault MM, Waldron H. The protective effect of marriage for survival: a review and update. Demography. 2011;48(2):481-506.
428. Reques L, Giraldez-Garcia C, Miqueleiz E, Belza MJ, Regidor E. Educational differences in mortality and the relative

importance of different causes of death: a 7-year follow-up study of Spanish adults. J Epidemiol Community Health. 2014;68(12):1151-60.
429. Reques L, Santos JM, Belza MJ, Martinez D, Regidor E. Inequalities in mortality at older ages decline with indicators of material wealth but persist with educational level. Eur J Public Health. 2015;25(6):990-5.
430. Riosmena F, Everett BG, Rogers RG, Dennis JA. Negative acculturation and nothing more? Cumulative disadvantage and mortality during the immigrant adaptation process among Latinos in the United States. International Migration Review. 2015;49(2):443-78.
431. Rogers NT, Power C, Pinto Pereira SM. Child maltreatment, early life socioeconomic disadvantage and all-cause mortality in mid-adulthood: findings from a prospective British birth cohort. BMJ Open. 2021 Sep 22;11(9):e050914.
432. Rogers RG, Everett BG, Onge JM, Krueger PM. Social, behavioral, and biological factors, and sex differences in mortality. Demography. 2010;47(3):555-78.
433. Rogers RG, Everett BG, Zajacova A, Hummer RA. Educational degrees and adult mortality risk in the United States. Biodemography Soc Biol. 2010;56(1):80-99.
434. Rogers RG, Hummer RA, Everett BG. Educational differentials in US adult mortality: An examination of mediating factors. Soc Sci Res. 2013;42(2):465-81.
435. Rogers RG, Hummer RA, Nam CB. Cigarette Smoking and Mortality. In: Rogers RG, Hummer RA, Nam CB, editors. Living and Dying in the USA: Behavioral, Health, and Social Differentials of Adult Mortality. San Diego: San Diego: Elsevier Science; 1999. p. xx+354-xx+.
436. Rogers RG, Hummer RA, Nam Cb. Perceived Health Status and Mortality. Living and Dying in the USA: Academic Press; 2000. p. 175-95.
437. Rogers RG, Hummer RA, Nam Cb. Mental and Addictive Disorders and Mortality. Living and Dying in the USA: Academic Press; 2000. p. 219-41.
438. Rogers RG, Hummer RA, Nam Cb. Alcohol Consumption and Mortality. Living and Dying in the USA: Academic Press; 2000. p. 259-70.
439. Rogers RG, Hummer RA, Nam Cb. Exercise and Mortality. Living and Dying in the USA: Academic Press; 2000. p. 271-84.
440. Rogers RG, Hummer RA, Nam Cb. The Influence of Other Health Behaviors on Mortality. Living and Dying in the USA: Academic Press; 2000. p. 285-300.
441. Rogers RG, Hummer RA, Nam Cb. The Sex Differential in Mortality. Living and Dying in the USA: Academic Press; 2000. p. 31-51.
442. Rogers RG, Hummer RA, Nam Cb. Religious Attendance, Social Participation, and adult Mortality. Living and Dying in the USA: Academic Press; 2000. p. 95-112.
443. Rogers RG, Hummer RA, Nam Cb. The Effects of Basic Socioeconomic Factors on Mortality. Living and Dying in the USA: Academic Press; 2000. p. 115-39.
444. Rogers RG, Hummer RA, Nam Cb. The Effect of Occupational Status on Mortality. Living and Dying in the USA: Academic Press; 2000. p. 141-58.
445. Rogers RG, Hummer RA, Nam Cb. Health Insurance Coverage and Mortality. Living and Dying in the USA: Academic Press; 2000. p. 159-71.
446. Rogers RG, Hummer RA, Nam Cb. Functional Limitations and Mortality. Living and Dying in the USA: Academic Press; 2000. p. 197-218.
447. Rogers RG, Krueger PM, Miech R, Lawrence EM, Kemp R. Nondrinker Mortality Risk in the United States. Popul Res Policy Rev. 2013;32(3):325-52.
448. Rogers RG, Lawrence EM, Montez JK. Alcohol's Collateral Damage: Childhood Exposure to Problem Drinkers and Subsequent Adult Mortality Risk. Soc Forces. 2016;95(2):809-36.
449. Rognerud MA, Zahl PH. Social inequalities in mortality: changes in the relative importance of income, education and household size over a 27-year period. Eur J Public Health. 2006;16(1):62-8.
450. Rogot E. A mortality study of 1.3 million persons by demographic, social and economic factors: 1979-1985 follow-up: US National Longitudinal Mortality Study: National Institutes of Health, National Heart, Lung, and Blood Institute; 1992.
451. Roos LL, Magoon J, Gupta S, Chateau D, Veugelers PJ. Socioeconomic determinants of mortality in two Canadian provinces: multilevel modelling and neighborhood context. Soc Sci Med. 2004;59(7):1435-47.
452. Rosengren A, Smyth A, Rangarajan S, Ramasundarahettige C, Bangdiwala SI, AlHabib KF, et al. Socioeconomic status and risk of cardiovascular disease in 20 low-income, middle-income, and high-income countries: the Prospective Urban Rural Epidemiologic (PURE) study. Lancet Glob Health. 2019;7(6):e748-e60.
453. Rosero-Bixby L, Dow WH. Surprising SES Gradients in mortality, health, and biomarkers in a Latin American population of adults. J Gerontol B Psychol Sci Soc Sci. 2009;64(1):105-17.
454. Ross CE, Masters RK, Hummer RA. Education and the gender gaps in health and mortality. Demography. 2012;49(4):1157-83.

455. Rostad B, Schei B, Lund Nilsen TI. Social inequalities in mortality in older women cannot be explained by biological and health behavioural factors -- results from a Norwegian health survey (the HUNT Study). Scand J Public Health. 2009;37(4):401-8.
456. Roy B, Kiefe CI, Jacobs DR, Goff DC, Lloyd-Jones D, Shikany JM, et al. Education, Race/Ethnicity, and Causes of Premature Mortality Among Middle-Aged Adults in 4 US Urban Communities: Results From CARDIA, 1985-2017. Am J Public Health. 2020 Apr;110(4):530–6.
457. Ruel E, Robert SA. A Model of Racial Residential History and Its Association with Self-Rated Health and Mortality Among Black and White Adults in the United States. Sociol Spectrum. 2009;29(4):443-66.
458. Rushing B, Ritter C, Burton RP. Race differences in the effects of multiple roles on health: longitudinal evidence from a national sample of older men. J Health Soc Behav. 1992;33(2):126-39.
459. Rutledge T, Matthews K, Lui LY, Stone KL, Cauley JA. Social networks and marital status predict mortality in older women: prospective evidence from the Study of Osteoporotic Fractures (SOF). Psychosom Med. 2003;65(4):688-94.
460. Sabanayagam C, Shankar A. Income is a stronger predictor of mortality than education in a national sample of US adults. J Health Popul Nutr. 2012;30(1):82-6.
461. Saenz JL, Wong R. A life course approach to mortality in Mexico. Salud Publica Mex. 2015;57 Suppl 1(0 1):S46-53.
462. Saikia N, Bora JK, Luy M. Socioeconomic disparity in adult mortality in India: estimations using the orphanhood method. Genus. 2019;75(1):1-14.
463. Saito T, Oksanen T, Shirai K, Fujiwara T, Pentti J, Vahtera J. Combined Effect of Marriage and Education on Mortality: A Cross-national Study of Older Japanese and Finnish Men and Women. J Epidemiol. 2020 Oct 5;30(10):442–9
464. Sandoval MH, Turra CM. El gradiente educativo en la mortalidad adulta en Chile. Revista Latinoamericana de Población. 2015;9(17):7-35.
465. Sandoval MH, Turra CM, Luz L. The Importance of Education for Middle- and Old-Age Mortality in Chile: Estimates From Panel Data Linked to Death Records. J Aging Health. 2022 Jan;34(1):71–7.
466. Sandoval MH, Alvear Portaccio ME. Marital Status, Living Arrangements and Mortality at Older Ages in Chile, 2004–2016. Int J Environ Res Public Health. 2022 Oct 22;19(21):13733.
467. Sartorius B. Modelling determinants, impact, and space-time risk of age-specific mortality in rural South Africa: integrating methods to enhance policy relevance. Glob Health Action. 2013;6(1):19239.
468. Saurel-Cubizolles MJ, Chastang JF, Menvielle G, Leclerc A, Luce D, group E. Social inequalities in mortality by cause among men and women in France. J Epidemiol Community Health. 2009;63(3):197-202.
469. Sautter JM, Thomas PA, Dupre ME, George LK. Socioeconomic status and the Black-White mortality crossover. Am J Public Health. 2012;102(8):1566-71.
470. Sauvaget C, Ramadas K, Fayette JM, Thomas G, Thara S, Sankaranarayanan R. Socio-economic factors & longevity in a cohort of Kerala State, India. Indian J Med Res. 2011;133(5):479-86.
471. Saydah SH, Imperatore G, Beckles GL. Socioeconomic status and mortality: contribution of health care access and psychological distress among U.S. adults with diagnosed diabetes. Diabetes Care. 2013;36(1):49-55.
472. Schafer MH, Upenieks L, MacNeil A. Disorderly Households, Self-Presentation, and Mortality: Evidence From a National Study of Older Adults. Res Aging. 2018;40(8):762-90.
473. Schnohr C, Højbjerre L, Riegels M, Ledet L, Larsen T, Schultz-Larsen K, et al. Does educational level influence the effects of smoking, alcohol, physical activity, and obesity on mortality? A prospective population study. Scandinavian journal of public health. 2004;32(4):250-6.
474. Schulz R, Beach SR, Ives DG, Martire LM, Ariyo AA, Kop WJ. Association between depression and mortality in older adults: the Cardiovascular Health Study. Arch Intern Med. 2000;160(12):1761-8.
475. Schulz R, Beach SR, Friedman EM. Caregiving Factors as Predictors of Care Recipient Mortality. Am J Geriatr Psychiatry. 2021 Mar;29(3):295–303.
476. Schwarz F. Widening educational disparities in all-cause mortality: An analysis of Austrian data with international comparisons. Vienna Yearbook of Population Research. 2007:93-105.
477. Seeman TE, Crimmins E, Huang MH, Singer B, Bucur A, Gruenewald T, et al. Cumulative biological risk and socio-economic differences in mortality: MacArthur studies of successful aging. Soc Sci Med. 2004;58(10):1985-97.
478. Seidelin UH, Ibfelt E, Andersen I, Steding-Jessen M, Hogdall C, Kjaer SK, et al. Does stage of cancer, comorbidity or lifestyle factors explain educational differences in survival after endometrial cancer? A cohort study among Danish women diagnosed 2005-2009. Acta Oncol. 2016;55(6):680-5.
479. Shariff-Marco S, Yang J, John EM, Kurian AW, Cheng I, Leung R, et al. Intersection of Race/Ethnicity and Socioeconomic Status in Mortality After Breast Cancer. J Community Health. 2015;40(6):1287-99.
480. Shariff-Marco S, Yang J, John EM, Sangaramoorthy M, Hertz A, Koo J, et al. Impact of neighborhood and individual socioeconomic status on survival after breast cancer varies by race/ethnicity: the Neighborhood and Breast Cancer Study. Cancer Epidemiol Biomarkers Prev. 2014;23(5):793-811.
481. Sheehan CM, Hayward MD. Black/white differences in mortality among veteran and non-veteran males. Soc Sci Res.

2019;79:101-14.
482. Shega JW, Andrew M, Kotwal A, Lau DT, Herr K, Ersek M, et al. Relationship between persistent pain and 5-year mortality: a population-based prospective cohort study. J Am Geriatr Soc. 2013;61(12):2135-41.
483. Shippee TP, Ferraro KF, Thorpe RJ. Racial disparity in access to cardiac intensive care over 20 years. Ethn Health. 2011;16(2):145-65.
484. Shkolnikov VM, Andreev EM, Jdanov DA, Jasilionis D, Kravdal O, Vagero D, et al. Increasing absolute mortality disparities by education in Finland, Norway and Sweden, 1971-2000. J Epidemiol Community Health. 2012;66(4):372-8.
485. Shkolnikov VM, Jasilionis D, Andreev EM, Jdanov DA, Stankuniene V, Ambrozaitiene D. Linked versus unlinked estimates of mortality and length of life by education and marital status: evidence from the first record linkage study in Lithuania. Soc Sci Med. 2007;64(7):1392-406.
486. Shkolnikov VM, Leon DA, Adamets S, Andreev E, Deev A. Educational level and adult mortality in Russia: an analysis of routine data 1979 to 1994. Soc Sci Med. 1998;47(3):357-69.
487. Signorello LB, Cohen SS, Williams DR, Munro HM, Hargreaves MK, Blot WJ. Socioeconomic status, race, and mortality: a prospective cohort study. Am J Public Health. 2014;104(12):e98-e107.
488. Singh GK, Siahpush M. All-cause and cause-specific mortality of immigrants and native born in the United States. Am J Public Health. 2001;91(3):392-9.
489. Singh GK, Yu SM. Trends and differentials in adolescent and young adult mortality in the United States, 1950 through 1993. Am J Public Health. 1996;86(4):560-4.
490. Skalicka V, Ringdal K, Witvliet MI. Socioeconomic inequalities in mortality and repeated measurement of explanatory risk factors in a 25 years follow-up. PLoS One. 2015;10(4):e0124690.
491. Skalicka V, van Lenthe F, Bambra C, Krokstad S, Mackenbach J. Material, psychosocial, behavioural and biomedical factors in the explanation of relative socio-economic inequalities in mortality: evidence from the HUNT study. Int J Epidemiol. 2009;38(5):1272-84.
492. Smith BT, Smith PM, Etches J, Mustard CA. Overqualification and risk of all-cause and cardiovascular mortality: evidence from the Canadian Census Mortality Follow-up Study (1991-2001). Can J Public Health. 2012;103(4):e297-302.
493. Smith SG, Jackson SE, Kobayashi LC, Steptoe A. Social isolation, health literacy, and mortality risk: Findings from the English Longitudinal Study of Ageing. Health Psychol. 2018;37(2):160-9.
494. Smith-Greenaway E, Brauner-Otto S, Axinn W. Offspring education and parental mortality: Evidence from South Asia. Soc Sci Res. 2018;76:157-68.
495. Snowdon DA, Ostwald SK, Kane RL. Education, survival, and independence in elderly Catholic sisters, 1936-1988. Am J Epidemiol. 1989;130(5):999-1012.
496. Socio-economic I, Euro-Coord HIVWGfCoOHERiEi. Inequalities by educational level in response to combination antiretroviral treatment and survival in HIV-positive men and women in Europe. AIDS. 2017;31(2):253-62.
497. Son M, Armstrong B, Choi JM, Yoon TY. Relation of occupational class and education with mortality in Korea. J Epidemiol Community Health. 2002;56(10):798-9.
498. Sorberg Wallin A, Allebeck P, Gustafsson JE, Hemmingsson T. Childhood IQ and mortality during 53 years' follow-up of Swedish men and women. J Epidemiol Community Health. 2018;72(10):926-32.
499. Sorlie PD, Backlund E, Keller JB. US mortality by economic, demographic, and social characteristics: the National Longitudinal Mortality Study. Am J Public Health. 1995;85(7):949-56.
500. Spoerri A, Schmidlin K, Richter M, Egger M, Clough-Gorr KM, Swiss National C. Individual and spousal education, mortality and life expectancy in Switzerland: a national cohort study. J Epidemiol Community Health. 2014;68(9):804-10.
501. Stratmann M, Björkenstam E, Dorner TE, Chen L, Helgesson M, Cullen AE, et al. Differences in all-cause and cause-specific mortality due to external causes and suicide between young adult refugees, non-refugee immigrants and Swedish-born young adults: The role of education and migration-related factors. PLoS One. 2022;17(12):e0279096.
502. Steenland K, Henley J, Thun M. All-cause and cause-specific death rates by educational status for two million people in two American Cancer Society cohorts, 1959-1996. Am J Epidemiol. 2002;156(1):11-21.
503. Stessman J, Cohen A, Hammerman-Rozenberg R, Bursztyn M, Azoulay D, Maaravi Y, et al. Holocaust survivors in old age: the Jerusalem Longitudinal Study. J Am Geriatr Soc. 2008;56(3):470-7.
504. Stewart QT. The shape of inequality: racial disparities in age-specific mortality. Biodemography Soc Biol. 2008;54(2):152-82.
505. Stringhini S, Dugravot A, Kivimaki M, Shipley M, Zins M, Goldberg M, et al. Do different measures of early life socioeconomic circumstances predict adult mortality? Evidence from the British Whitehall II and French GAZEL studies. J Epidemiol Community Health. 2011;65(12):1097-103.
506. Stringhini S, Dugravot A, Shipley M, Goldberg M, Zins M, Kivimaki M, et al. Health behaviours, socioeconomic status, and mortality: further analyses of the British Whitehall II and the French GAZEL prospective cohorts. PLoS

Med. 2011;8(2):e1000419.
507. Sudharsanan N, Zhang Y, Payne CF, Dow W, Crimmins E. Education and adult mortality in middle-income countries: Surprising gradients in six nationally-representative longitudinal surveys. SSM Popul Health. 2020 Dec;12:100649.
508. Sudore RL, Yaffe K, Satterfield S, Harris TB, Mehta KM, Simonsick EM, et al. Limited literacy and mortality in the elderly: the health, aging, and body composition study. J Gen Intern Med. 2006;21(8):806-12.
509. Sugisawa H, Liang J, Liu X. Social networks, social support, and mortality among older people in Japan. J Gerontol. 1994;49(1):S3-13.
510. SUH GH. Predictors of mortality in an aging community-based cohort in Korea. Psychogeriatrics. 2006;6(1):10-8.
511. Sullivan AR. Mortality Differentials and Religion in the U.S.: Religious Affiliation and Attendance. J Sci Study Relig. 2010;49(4):740-53.
512. Sullivan AR, Fenelon A. Patterns of widowhood mortality. J Gerontol B Psychol Sci Soc Sci. 2014;69(1):53-62.
513. Sunder M. Toward generation XL: anthropometrics of longevity in late 20th-century United States. Econ Hum Biol. 2005;3(2):271-95.
514. Sundquist J, Johansson SE. Indicators of socio-economic position and their relation to mortality in Sweden. Soc Sci Med. 1997;45(12):1757-66.
515. Sundquist J, Johansson SE. Self reported poor health and low educational level predictors for mortality: a population based follow up study of 39,156 people in Sweden. J Epidemiol Community Health. 1997;51(1):35-40.
516. Sundquist J, Johansson SE. The influence of country of birth on mortality from all causes and cardiovascular disease in Sweden 1979-1993. Int J Epidemiol. 1997;26(2):279-87.
517. Suresh S, Sabanayagam C, Shankar A. Socioeconomic status, self-rated health, and mortality in a multiethnic sample of US adults. J Epidemiol. 2011;21(5):337-45.
518. Syse A, Strand BH, Naess O, Steingrimsdottir OA, Kumar BN. Differences in all-cause mortality: A comparison between immigrants and the host population in Norway 1990-2012. Demographic Research. 2016;34:615-55.
519. Søndergaard G, Mortensen LH, Nybo Andersen A-M, Andersen PK, Dalton SO, Madsen M, et al. Does shared family background influence the impact of educational differences on early mortality? Am J Epidemiol. 2012;176(8):675-83.
520. Saarela J, Finnas F. Mortality inequality in two native population groups. Popul Stud (Camb). 2005;59(3):313-20.
521. Saarela J, Finnas F. Geographic Ancestry and Cause-specific Mortality in a National Population. Population Research and Policy Review. 2009;28(2):169-94.
522. Tamayo-Fonseca N, Quesada JA, Nolasco A, Melchor I, Moncho J, Pereyra-Zamora P, et al. Self-rated health and mortality: a follow-up study of a Spanish population. Public Health. 2013;127(12):1097-104.
523. Tani Y, Kondo N, Nagamine Y, Shinozaki T, Kondo K, Kawachi I, et al. Childhood socioeconomic disadvantage is associated with lower mortality in older Japanese men: the JAGES cohort study. Int J Epidemiol. 2016;45(4):1226-35.
524. Tanner NT, Gebregziabher M, Hughes Halbert C, Payne E, Egede LE, Silvestri GA. Racial Differences in Outcomes within the National Lung Screening Trial. Implications for Widespread Implementation. Am J Respir Crit Care Med. 2015;192(2):200-8.
525. Tenconi MT, Devoti G, Comelli M. Role of socioeconomic indicators in the prediction of all causes and coronary heart disease mortality in over 12,000 men--The Italian RIFLE pooling project. Eur J Epidemiol. 2000;16(6):565-71.
526. Thinggaard M, McGue M, Jeune B, Osler M, Vaupel JW, Christensen K. Survival Prognosis in Very Old Adults. J Am Geriatr Soc. 2016;64(1):81-8.
527. Tjepkema M, Wilkins R, Long A. Cause-specific mortality by education in Canada: a 16-year follow-up study. Health Rep. 2012;23(3):23-31.
528. Tjepkema M, Wilkins R, Long A. Socio-economic inequalities in cause-specific mortality: a 16-year follow-up study. Can J Public Health. 2013;104(7):e472-8.
529. Tjepkema M, Wilkins R, Senecal S, Guimond E, Penney C. Mortality of Metis and registered Indian adults in Canada: an 11-year follow-up study. Health Rep. 2009;20(4):31-51.
530. Tjepkema M, Wilkins R, Senecal S, Guimond E, Penney C. Mortality of urban Aboriginal adults in Canada, 1991-2001. Chronic Dis Can. 2010;31(1):4-21.
531. Tobiasz-Adamczyk B, Bartoszewska E, Brzyski P, Kopacz M. Long-term consequences of education, working conditions, and health-related behaviors on mortality patterns in older age. A 17-year observational study in Krakow, Poland. Int J Occup Med Environ Health. 2007;20(3):247-56.
532. Todd MA, Shkolnikov VM, Goldman N. Why are well-educated Muscovites more likely to survive? Understanding the biological pathways. Soc Sci Med. 2016;157:138-47.
533. Torssander J. From child to parent? The significance of children's education for their parents' longevity. Demography. 2013;50(2):637-59.
534. Torssander J, Erikson R. Marital partner and mortality: the effects of the social positions of both spouses. J Epidemiol Community Health. 2009;63(12):992-8.
535. Torssander J, Erikson R. Stratification and Mortality-A Comparison of Education, Class, Status, and Income. European Sociological Review. 2010;26(4):465-74.

536. Tottenborg SS, Lange P, Johnsen SP, Nielsen H, Ingebrigtsen TS, Thomsen RW. Socioeconomic inequalities in adherence to inhaled maintenance medications and clinical prognosis of COPD. Respir Med. 2016;119:160-7.
537. Trevisan C, Veronese N, Maggi S, Baggio G, Toffanello ED, Zambon S, et al. Factors Influencing Transitions Between Frailty States in Elderly Adults: The Progetto Veneto Anziani Longitudinal Study. J Am Geriatr Soc. 2017;65(1):179-84.
538. Turiano NA, Spiro A, 3rd, Mroczek DK. Openness to experience and mortality in men: analysis of trait and facets. J Aging Health. 2012;24(4):654-72.
539. Ullits LR, Ejlskov L, Mortensen RN, Hansen SM, Kraemer SR, Vardinghus-Nielsen H, et al. Socioeconomic inequality and mortality--a regional Danish cohort study. BMC Public Health. 2015;15:490.
540. Valkonen T, Martelin T, Rimpelä A. Socio-economic mortality differences in Finland 1971-1985: Central Statistical Office of Finland; 1990.
541. Van Doorslaer E, Gerdtham U-G. Does inequality in self-assessed health predict inequality in survival by income? Evidence from Swedish data. Soc Sci Med. 2003;57(9):1621-9.
542. van Hedel K, Avendano M, Berkman LF, Bopp M, Deboosere P, Lundberg O, et al. The contribution of national disparities to international differences in mortality between the United States and 7 European countries. Am J Public Health. 2015;105(4):e112-9.
543. van Hedel K, van Lenthe FJ, Oude Groeniger J, Mackenbach JP. What's the difference? A gender perspective on understanding educational inequalities in all-cause and cause-specific mortality. BMC Public Health. 2018;18(1):1105.
544. Van Rossum C. Inequalities in health. Socioeconomic status and mortality in Dutch elderly people. The Rotterdam Study. The European Journal of Public Health. 2000;10(4):255-61.
545. van Zwieten A, Wong G, Ruospo M, Palmer SC, Teixeira-Pinto A, Barulli MR, et al. Associations of Cognitive Function and Education Level With All-Cause Mortality in Adults on Hemodialysis: Findings From the COGNITIVE-HD Study. Am J Kidney Dis. 2019;74(4):452-62.
546. Vanthomme K, Gadeyne S. Unemployment and cause-specific mortality among the Belgian working-age population: The role of social context and gender. PLoS One. 2019;14(5):e0216145.
547. Vanthomme K, Vandenheede H. Migrant mortality differences in the 2000s in Belgium: interaction with gender and the role of socioeconomic position. Int J Equity Health. 2019;18(1):96.
548. Vazquez-Castillo P. Better alone? The impact of living arrangements on mortality of Costa Rican older adults. Rev bras estud popul. 2023 Apr 14;40:e0236.
549. Veenstra M, Løset GK, Daatland SO. Socioeconomic Inequalities in Mortality After Age 67: The Contribution of Psychological Factors. Front Psychol. 2021;12:717959.
550. Vescio MF, Smith GD, Giampaoli S, Group MR. Socio-economic-position overall and cause-specific mortality in an Italian rural population. Eur J Epidemiol. 2003;18(11):1051-8.
551. Vierboom YC. The contribution of differences in adiposity to educational disparities in mortality in the United States. Demogr Res. 2017;37:1735-60.
552. Vierboom YC. Trends in alcohol-related mortality by educational attainment in the U.S., 2000–2017. Popul Res Policy Rev. 2020 Feb;39(1):77–97.
553. Wallace M. Adult mortality among the descendants of immigrants in England and Wales: does a migrant mortality advantage persist beyond the first generation? Journal of Ethnic and Migration Studies. 2016;42(9):1558-77.
554. Wallace M, Khlat M, Guillot M. Mortality advantage among migrants according to duration of stay in France, 2004-2014. BMC Public Health. 2019;19(1):327.
555. Wallace M, Kulu H. Can the salmon bias effect explain the migrant mortality advantage in England and Wales? Population, Space and Place. 2018;24(8):e2146.
556. Wandell P, Carlsson AC, Gasevic D, Holzmann MJ, Arnlov J, Sundquist J, et al. Socioeconomic factors and mortality in patients with atrial fibrillation-a cohort study in Swedish primary care. Eur J Public Health. 2018;28(6):1103-9.
557. Wang J, Lin W, Chang LH. The linear relationship between the Vulnerable Elders Survey-13 score and mortality in an Asian population of community-dwelling older persons. Arch Gerontol Geriatr. 2018;74:32-8.
558. Wang L, Yi Z. Marital status and all-cause mortality rate in older adults: a population-based prospective cohort study. BMC Geriatr. 2023 Apr 4;23(1):214.
559. Wang T, Zhao Z, Yu X, Zeng T, Xu M, Xu Y, et al. Age-specific modifiable risk factor profiles for cardiovascular disease and all-cause mortality: a nationwide, population-based, prospective cohort study. Lancet Reg Health West Pac. 2021 Dec;17:100277.
560. Wang R, Feng Q, Dupre ME, Guo A, Qiu L, Hao L, et al. Objective and subjective financial status and mortality among older adults in China. Arch Gerontol Geriatr. 2019;81:182-91.
561. Warren JR, Muller C, Hummer RA, Grodsky E, Humphries M. Which Aspects of Education Matter for Early Adult Mortality? Evidence from the High School and Beyond Cohort. Socius. 2020;6:10.1177/2378023120918082.
562. Wei M, Valdez RA, Mitchell BD, Haffner SM, Stern MP, Hazuda HP. Migration status, socioeconomic status, and mortality rates in Mexican Americans and non-Hispanic whites: the San Antonio Heart Study. Ann Epidemiol.

1996;6(4):307-13.
563. Weiss A, Costa PT, Jr. Domain and facet personality predictors of all-cause mortality among Medicare patients aged 65 to 100. Psychosom Med. 2005;67(5):724-33.
564. Weitoft GR, Rosén M. Is perceived nervousness and anxiety a predictor of premature mortality and severe morbidity? A longitudinal follow up of the Swedish survey of living conditions. J Epidemiol Community Health. 2005;59(9):794-8.
565. Welaga P. The impact of migration on adult mortality in rural South Africa: Do people migrate into rural areas to die? 2007.
566. Welon Z, Bielicki T, Rogucka E, Malina RM. Effect of education and marital status on premature mortality among urban adults in Poland, 1988-1989. Am J Hum Biol. 1999;11(3):397-403.
567. Welsh C, Welsh P, Mark PB, Celis-Morales CA, Lewsey J, Gray SR, et al. Association of Total and Differential Leukocyte Counts With Cardiovascular Disease and Mortality in the UK Biobank. Arterioscler Thromb Vasc Biol. 2018;38(6):1415-23.
568. Whisman MA, Gilmour AL, Salinger JM. Marital satisfaction and mortality in the United States adult population. Health Psychol. 2018;37(11):1041-4.
569. Wight RG, Cummings JR, Karlamangla AS, Aneshensel CS. Urban neighborhood context and mortality in late life. J Aging Health. 2010;22(2):197-218.
570. Wilkins R, Tjepkema M, Mustard C, Choinière R. The Canadian census mortality follow-up study, 1991 through 2001. Health Rep. 2008;19(3):25.
571. Wilper AP, Woolhandler S, Lasser KE, McCormick D, Bor DH, Himmelstein DU. Health insurance and mortality in US adults. Am J Public Health. 2009;99(12):2289-95.
572. Winkleby MA, Cubbin C. Influence of individual and neighbourhood socioeconomic status on mortality among black, Mexican-American, and white women and men in the United States. J Epidemiol Community Health. 2003;57(6):444-52.
573. Wolfe J, Bauldry S, Hardy M, Pavalko EK. Multigenerational socioeconomic attainments and mortality among older men: An adjacent generations approach. Demographic Research. 2018;39:719-52.
574. Woo H, Zajacova A. Predictive strength of self-rated health for mortality risk among older adults in the United States: does it differ by race and ethnicity? Res Aging. 2017;39(7):879-905.
575. Woodside J, Yarnell J, Patterson C, Arveiler D, Amouyel P, Ferrières J, et al. Do lifestyle behaviours explain socioeconomic differences in all-cause mortality, and fatal and non-fatal cardiovascular events? Evidence from middle aged men in France and Northern Ireland in the PRIME Study. Prev Med. 2012;54(3-4):247-53.
576. Woodward M, Peters SA, Batty GD, Ueshima H, Woo J, Giles GG, et al. Socioeconomic status in relation to cardiovascular disease and cause-specific mortality: a comparison of Asian and Australasian populations in a pooled analysis. BMJ Open. 2015;5(3):e006408.
577. Xie W. Mortality differential for various levels of education in China. Chinese journal of population science. 1996;8(1):41-9.
578. Yahirun JJ, Sheehan CM, Hayward MD. Adult children's education and changes to parents' physical health in Mexico. Soc Sci Med. 2017;181:93-101.
579. Yang K, Zhang Y, Saito E, Rahman MS, Gupta PC, Sawada N, et al. Association between educational level and total and cause-specific mortality: a pooled analysis of over 694 000 individuals in the Asia Cohort Consortium. BMJ Open. 2019;9(8):e026225.
580. Yang L, Martikainen P, Silventoinen K. Effects of Individual, Spousal, and Offspring Socioeconomic Status on Mortality Among Elderly People in China. J Epidemiol. 2016;26(11):602-9.
581. Yang W-S, Shih Y-C, Li Y-T. Living arrangements, coresidence preference, and mortality risk among older Taiwanese. Asian Education and Development Studies. 2017.
582. Yao L, Robert SA. The contributions of race, individual socioeconomic status, and Neighborhood socioeconomic context on the self-rated health trajectories and mortality of older adults. Res Aging. 2008;30(2):251-73.
583. Yao L, Robert SA. Examining the racial crossover in mortality between African American and white older adults: a multilevel survival analysis of race, individual socioeconomic status, and neighborhood socioeconomic context. J Aging Res. 2011;2011.
584. Yeung WJ, Xu ZH. Economic Stress, Quality of Life, and Mortality for the Oldest-Old in China. Social Indicators Research. 2012;108(1):131-52.
585. Yi Z, Gu D, Land KC. The association of childhood socioeconomic conditions with healthy longevity at the oldest-old ages in China. Demography. 2007;44(3):497-518.
586. Yu ES, Kean YM, Slymen DJ, Liu WT, Zhang M, Katzman R. Self-perceived health and 5-year mortality risks among the elderly in Shanghai, China. Am J Epidemiol. 1998;147(9):880-90.
587. Yu S, Guo X, Li G, Yang H, Zheng L, Sun Y. Low educational status correlates with a high incidence of mortality among hypertensive subjects from Northeast Rural China. Front Public Health. 2022 Aug 25;10:951930.

588. Yu Y, Booth H. The changing contribution of smoking to educational inequality in female mortality. J Popul Res. 2014;31:97-110.
589. Zajacova A. Education, gender, and mortality: Does schooling have the same effect on mortality for men and women in the US? Soc Sci Med. 2006;63(8):2176-90.
590. Zajacova A, Hummer RA. Gender differences in education effects on all-cause mortality for white and black adults in the United States. Soc Sci Med. 2009;69(4):529-37.
591. Zarulli V. Post-War Migration Flows and Disparities in Mortality from Age 50 Years Onwards: the Case of Turin in Italy. Population Space and Place. 2016;22(1):54-67.
592. Zarulli V, Marinacci C, Costa G, Caselli G. Mortality by education level at late-adult ages in Turin: a survival analysis using frailty models with period and cohort approaches. BMJ Open. 2013;3(7).
593. Zeng Y, Gu D, Purser J, Hoenig H, Christakis N. Associations of environmental factors with elderly health and mortality in China. Am J Public Health. 2010;100(2):298-305.
594. Zhang W, Li S, Silverstein M. The effects of inter-generational support on the mortality of older people in rural China. Asian Population Studies. 2005;1(3):325-38.
595. Zhang X, Dupre ME, Qiu L, Zhou W, Zhao Y, Gu D. Urban-rural differences in the association between access to healthcare and health outcomes among older adults in China. BMC Geriatr. 2017;17(1):151.
596. Zhang YB, Li Y, Geng TT, Pan XF, Zhou YF, Liu G, et al. Overall lifestyles and socioeconomic inequity in mortality and life expectancy in China: the China health and nutrition survey. Age Ageing. 2022 Jul 1;51(7):afac167.
597. Zheng H, Yang Y. Population heterogeneity in the impact of body weight on mortality. Soc Sci Med. 2012;75(6):990-6.
598. Zhou G, Liu X, Xu G, Liu X, Zhang R, Zhu W. The effect of socioeconomic status on three-year mortality after first-ever ischemic stroke in Nanjing, China. BMC Public Health. 2006;6:227.
599. Zhao Y, Xu X, Dupre ME, Xie Q, Qiu L, Gu D. Individual-level factors attributable to urban-rural disparity in mortality among older adults in China. BMC Public Health. 2020 Sep 29;20(1):1472.
600. Zhu HY, Xie Y. Socioeconomic differentials in mortality among the oldest old in China. Res Aging. 2007;29(2):125-43.
601. Zimmer Z, Kaneda T, Spess L. An examination of urban versus rural mortality in China using community and individual data. J Gerontol B Psychol Sci Soc Sci. 2007;62(5):S349-57.
602. Zimmer Z, Martin LG, Ofstedal MB, Chuang YL. Education of adult children and mortality of their elderly parents in Taiwan. Demography. 2007;44(2):289-305.
603. Zou S, Wang Z, Tang K. Social inequalities in all-cause mortality among adults with multimorbidity: a 10-year prospective study of 0.5 million Chinese adults. Int Health. 2023 Mar 1;15(2):123–33.

Supplementary Figure 11. Normalised relative risks per one year of education, shown for all study data separately by super-region and region. Colour indicates whether the effect measure was automatically trimmed in the global model. Full study titles corresponding with axis label are provided in the supplementary Appendix 3.csv.
